# *Nannochloropsis* Genomes Reveal Evolution of Microalgal Oleaginous Traits

**DOI:** 10.1371/journal.pgen.1004094

**Published:** 2014-01-09

**Authors:** Dongmei Wang, Kang Ning, Jing Li, Jianqiang Hu, Danxiang Han, Hui Wang, Xiaowei Zeng, Xiaoyan Jing, Qian Zhou, Xiaoquan Su, Xingzhi Chang, Anhui Wang, Wei Wang, Jing Jia, Li Wei, Yi Xin, Yinghe Qiao, Ranran Huang, Jie Chen, Bo Han, Kangsup Yoon, Russell T. Hill, Yonathan Zohar, Feng Chen, Qiang Hu, Jian Xu

**Affiliations:** 1 CAS Key Laboratory of Biofuels, Shandong Key Laboratory of Energy Genetics and BioEnergy Genome Center, Qingdao Institute of BioEnergy and Bioprocess Technology, Chinese Academy of Sciences, Qingdao, Shandong, China; 2 Laboratory for Algae Research and Biotechnology, College of Technology and Innovation, Arizona State University, Mesa, Arizona, United States of America; 3 Institute of Marine and Environmental Technology, University of Maryland Center for Environmental Science, Baltimore, Maryland, United States of America; 4 Center for Microalgal Biotechnology and Biofuels, Institute of Hydrobiology, Chinese Academy of Sciences, Wuhan, Hubei, China; 5 Institute of Marine Environmental Technology and Department of Marine Biotechnology, University of Maryland Baltimore County, Baltimore, Maryland, United States of America; University of Utah School of Medicine, United States of America

## Abstract

Oleaginous microalgae are promising feedstock for biofuels, yet the genetic diversity, origin and evolution of oleaginous traits remain largely unknown. Here we present a detailed phylogenomic analysis of five oleaginous *Nannochloropsis* species (a total of six strains) and one time-series transcriptome dataset for triacylglycerol (TAG) synthesis on one representative strain. Despite small genome sizes, high coding potential and relative paucity of mobile elements, the genomes feature small cores of ca. 2,700 protein-coding genes and a large pan-genome of >38,000 genes. The six genomes share key oleaginous traits, such as the enrichment of selected lipid biosynthesis genes and certain glycoside hydrolase genes that potentially shift carbon flux from chrysolaminaran to TAG synthesis. The eleven type II diacylglycerol acyltransferase genes (*DGAT-2*) in every strain, each expressed during TAG synthesis, likely originated from three ancient genomes, including the secondary endosymbiosis host and the engulfed green and red algae. Horizontal gene transfers were inferred in most lipid synthesis nodes with expanded gene doses and many glycoside hydrolase genes. Thus multiple genome pooling and horizontal genetic exchange, together with selective inheritance of lipid synthesis genes and species-specific gene loss, have led to the enormous genetic apparatus for oleaginousness and the wide genomic divergence among present-day *Nannochloropsis*. These findings have important implications in the screening and genetic engineering of microalgae for biofuels.

## Author Summary

Microalgae are promising feedstock for biofuels. However, the diversity, origin and evolution of oil-producing microalgal genomes in general, and those of their oleaginous traits in particular, remain poorly understood. We present five new genomes of the oleaginous microalgae *Nannochloropsis* spp. that allow genus-, species- and strain-level genomic comparison. With each *Nannochloropsis* genome encoding approximately 6,562–9,915 genes, a core genome of ca. 2,700 genes and a large pan-genome of >38,000 genes were found. The genomes share key genetic features such as gene dose expansion of selected nodes in lipid biosynthesis pathways. Evidence of horizontal gene transfers, primarily from bacteria, was found in most of these nodes. However, the eleven type II acyl-CoA:diacylglycerol acyltransferase genes (*DGAT-2*),the highest gene dose reported among known organisms, likely originated from three ancient genomes of the secondary endosymbiosis host and the engulfed green and red algae; they were strictly vertically inherited in each of the *Nannochloropsis* spp. Thus, multiple genome pooling and horizontal genetic exchange have underlain the enormous genetic makeup underlying TAG production in present-day *Nannochloropsis*.

## Introduction

Microalgae represent a promising source of biomass feedstock for fuels and chemicals because many species possess the ability to grow rapidly and synthesize large amounts of storage neutral lipids in a form of triacylglycerol (TAG) from sunlight and carbon dioxide. They can be cultivated on non-arable land with non-potable water and waste streams (e.g., flue gases and wastewaters) and thus pose little competition to food crops while providing environmental benefits [1]. However, understanding of the divergence and evolution of oleaginous traits and the underlying evolutionary forces and molecular mechanisms in microalgae remains elusive [2].

*Nannochloropsis* is a genus of unicellular photosynthetic microalgae in the class Eustigmatophyceae, ranging in size from 2–5 µm and widely distributed in marine, fresh and brackish waters. They are of interest as a potential feedstock for fuels and high-value products because they tolerate broad enivronmental and culture conditions while growing rapidly and producing large amounts of TAG and eicosapentaenoic acid, a high-value polyunsaturated fatty acid [3]. A homologous recombination–based gene transformation system was recently established in *Nannochloropsis* [4], making trait improvement in this organism possible for overproduction of biomass or desirable products.

**Figure 1:**
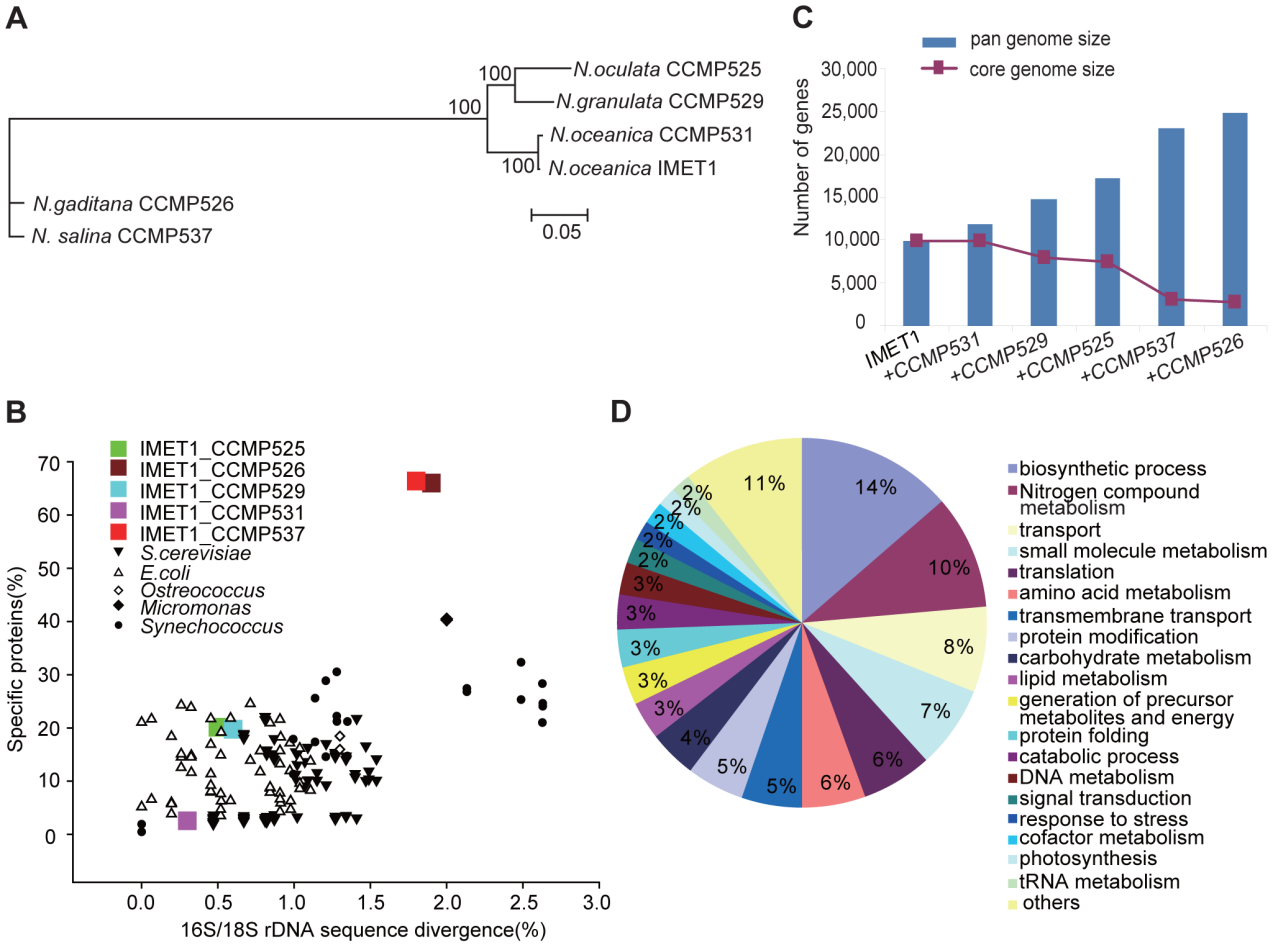
Structural features of the six *Nannochloropsis* genomes. (**A**) Whole-genome based phylogeny of *Nannochloropsis*. A maximum likelihood (consensus) tree was generated using the PhyML program (JTT model) with 1,000 replicates based on the 1,085 six-way single-copy orthologous gene sets identified from the six *Nannochloropsis* genomes (**Text S1**). Percentages of replicate trees in the bootstrap test are shown next to the branches. (**B**) Genome divergence in *Nannochloropsis*. For each pair of genomes that consists of IMET1 and another *Nannochloropsis* strain, the percentages of strain-specific proteins versus their discrepancies in the full-length 18S rDNA sequence were plotted. Ten *S. cerevisiae* strains, eight *E. coli* strains, nine *Synechococcus* strains and two each of *Ostreococcus* and *Micromonas* were also included. For prokaryotic organisms including *E. coli* and *Synechococcus*, the percentages of strain-specific proteins were plotted against the discrepancies in 16S rDNA sequences. (**C**) The number of genes from the *Nannochloropsis* genomes and the *Nannochloropsis* core, with successive inclusion of each additional strain. (**D**) Functional categorization of *Nannochloropsis* core proteins. GO Slim terms corresponding to each GO term are presented.

Here we present a comparative analysis of six genomes of oleaginous *Nannochloropsis* spp. that includes two *N. oceanica* strains (IMET1 and CCMP531) and one strain from each of four other recognized species: *N. salina* (CCMP537), *N. gaditana* (CCMP526, which was previously reported [5]), *N. oculata* (CCMP525) and *N. granulata* (CCMP529) (**Figure 1A**; **Figure S1**; **Figure S2**; **Table S1A, S1B**). Moreover, for *N. oceanica* IMET1, the diversity of transcripts was mapped to support gene prediction by sequencing cDNA libraries using 454-based long reads. Furthermore, transcript dynamics were measured via a two-condition (control condition and nitrogen starved condition), three time-point temporal series of transcriptomes during TAG accumulation using Illumina-based short-reads (**Text S1**). Integration of phenotypic, genomic and transcriptomic data across a *Nannochloropsis* phylogeny provided new insights into the molecular mechanisms driving the diversity and evolution of these oleaginous microalgae.

## Results

### (I) General features of *Nannochloropsis* genomes

The genome sizes of the six oleaginous *Nannochloropsis* species and strains range from 25.38 to 32.07 Mb (**Figure 1A**; **Table 1**). For strain IMET1, the nuclear, chloroplast and mitochondria genomes are 31.36 Mb, 117.5 Kb and 38 Kb, respectively, totaling 31.5 Mb. Pulse-field gel electrophoresis on total IMET1 DNA confirmed the genome size and indicated the presence of 22 chromosomes (**Figure S3A**, **S3B**). For IMET1, 9,754, 126 and 35 protein-coding genes were predicted in the nuclear, chloroplast and mitochondrial genomes, respectively (**Table 1**). Among the nuclear genes, 93.4% (9,111) were covered by mRNA-Seq data (defined as >80% of the transcribed region mapped by at least 10 reads; **Table S1C, S1D, Text S1**).

**Table 1:** Genomic features of the *Nannochloropsis* genomes.

Microalgae	*N. oceanica* IMET1	*N. oceanica* CCMP531	*N. granulate* CCMP529	*N. oculata* CCMP525	*N. salina* CCMP537	*N. gaditana* CCMP526*
**Whole genome**						
Assembly size (Mb)	30.1	35.5	30.1	34.5	26.9	29.0
G+C content (%)	53.7	53.8	54.6	54.2	54.2	54.2
Predicted gene number	9,915	7,268	8,060	7,254	6,562	8,738
Genes having blast hits in NCBI NR database	6,853	3,584	4,014	3,557	3,789	5,467
Genes with functional annotation	5,121	2,528	2,798	3,321	1,378	3,704
Predicted tRNA number	80	79	85	82	85	85
Predicted LTR number	26	24	6	25	4	33
LTR length (Kbp)	24.3	9.4	2.3	25.6	3.4	41.8
**Mitochondrial genome**						
Size (bases)	38,057	38,057	38,791	38,444	41, 907	38,981
G+C content (%)	31.9	31.9	32.0	31.8	31.4	31.4
Predicted gene number	35	35	35	35	36	35
Predicted tRNA number	28	28	28	28	28	26
**Chloroplast genome**						
Size (bases)	117,548	117,634	117,672	117,463	114,883	114,937
G+C content (%)	33.6	33.6	33.4	33.4	33.1	33.0
Predicted gene number	126	126	126	126	123	122
Predicted tRNA number	34	34	34	34	33	28

, data cited from the genome paper of *N. gaditana* CCMP526 [5].

These *Nannochloropsis* genomes are all relatively compact (**Table S2**; [5], [6]), much smaller than that of the model green microalga *Chlamydomonas reinhardtii* (121 Mb; [7]). The IMET1 genome features a higher coding potential (52.1%) than the diatom *Thalassiosira pseudonana* (32.7%; [8]), which has a similar genome size. Mobile elements can be prevalent in algae [e.g. *T. pseudonana* harbors 238 long terminal repeats (LTRs) totaling 1.56 Mb], but they are rather limited in IMET1, as only 26 LTRs (24.3 Kb in total), along with several DNA transposons (864 bp in total), are present in the genome without transposases (**Table S2**). The relative paucity of mobile elements appears to be one shared feature of the six *Nannochloropsis* strains (**Table 1**)

### (II) Divergence of *Nannochloropsis* genomes

Genomic diversity and divergence defining microalgal genera, species or strains are largely unknown [9]. A whole-genome phylogeny of *Nannochloropsis* (**Figure 1A**) was constructed from 1,085 single-copy-orthologous groups identified from the six genomes, which is consistent with the 18S-based phylogeny (**Figure S2**). Among the five *Nannochloropsis* species, *N. granulata* and *N. oculata* have a recent common ancestor and are clustered with the two *N. oceanica* strains. Among the 1,085 single-copy orthologous groups, 628 (61.7%) exhibited congruent phylogenies with the whole-genome phylogeny. The mean K_a_/K_s_ of 0.08 calculated from these candidate phylogenetic markers in the nuclear genomes was higher than in the chloroplast genomes (0.031) and in the mitochondrial genomes (0.064). Among these candidate markers, 25 genes exhibited sequence variations large enough to differentiate each of the species and strains (density of inter-species SNP at 20–40% and intra-species over 1%), but allowed for the design of consensus flanking PCR primers (**Dataset S1**). Those with the highest resolution included cytochrome P450, btaA, plastid ribosomal protein S1 and transaldolase etc., which represent novel phylogenetic markers that are more sensitive than 18S or ITS sequences (0.16% and 0.52% in intra-species SNP density, respectively) in strain-typing of *Nannochloropsis*.

Between any two genomes among the six *Nannochloropsis* strains, 35% of protein-coding genes (ranging from 2.6% between the two *N. oceanica* strains to 66.4% between IMET1 and *N. salina* CCMP537) were not found in the other genome on average, despite >98% similarity in full-length 18S rDNA. This places their inter-species genome divergence higher than the green algae studied and their intra-species divergence comparable to *E. coli* and yeast (**Figure 1B**). Therefore, the *Nannochloropsis* pan-genome, as defined by the six strains, consists of at least 38,000 protein-coding genes, along with a relatively small pool of *Nannochloropsis* core genes (e.g., 2,734 genes in IMET1) that are shared by the six strains (**Figure 1C**, **Text S1**). Most (93.2%) of these core genes have blast hits in NCBI non-redundant (NR) database, of which 94% were functionally annotated. The core genes mostly encode proteins involved in DNA, RNA, and protein synthesis and modification, transporters, signal transduction and central metabolic pathways (**Figure 1D**; for functional classification based on molecular function and cellular component, see **Figure S4**). The accessory genes, referring to those missing in at least one strain, mainly encode (*i*) central metabolism such as carbohydrate, lipid, energy, and nucleotide and amino acid metabolism (which are overlapped with the core genes), (*ii*) secondary metabolism and N-glycan biosynthesis (which are complementary to the core genes, and (*iii*) unknown functions (**Figure S5**).

There were 164–1,513 genes that were strain-specific among the six genomes. In contrast to the 2,734 *Nannochloropsis* core genes, of which 96.7% were supported by our mRNA-Seq reads, 11.0% (18) of the 164 IMET1-specific proteins lacked such supports, suggesting the possible presence of pseudogenes or false positives in gene prediction. Among the IMET1-specific genes with mRNA support, 94.5% were putative novel genes without any known homologs (Blast hits) in the NCBI NR database. It is possible that some of them might have horizontally transferred from unsequenced species. Among strain-specific genes with functional annotations, most were involved in responses to freezing in *N. oculata* CCMP525, *N. granulata* CCMP529 and *N. oceanica* CCMP531. In *N. gaditana* CCMP526, transporters were prevalent, while in *N. salina* CCMP537, no significant enrichment was found in any processes (**Figure S6**).

Correlation analysis revealed that the core and accessory genes exhibited different sequence and transcriptional features under the experimental conditions tested (**Text S2**). The accessory genes tend to be under lower purifying pressure while lower transcriptional levels (**Text S2; Figure S7**; **Figure S8**), supporting a link between sequence evolution and transcriptional activity [10], [11], [12].

To probe the link between the accessory genes and divergence of the genomes, protein-coding genes in the six *Nannochloropsis* were classified into different groups based on the number of strains in which they were present (thus those present in all the six strains were part of the *Nannochloropsis* core). The most prominent group included the genes shared by four of the strains, in which the majority (97.3%) were found in the phylogenetically closely related species, i.e., *N. oceanica* (two strains), *N. granulata* and *N. oculata*. The absence of these genes in the other two species explained the small number of *Nannochloropsis* core genes (**Figure S9A**). These genes might have been present in the common ancestors of heterokonts and later lost in *N. salina* and *N. gaditana*, as >60% of them were found in other heterokonts (e.g., diatoms, *Ectocarpus* and other non-photosynthetic heterokonts such as *Phytophthora*). The functions supported by these genes were similar to those of core genes, with oxidation-reduction, transmembrane transport and protein-related metabolism being dominant. This does not support the presence of functional bias in the gene loss events (**Figure S9B**).

### (III) Features of paralogous protein groups in the *Nannochloropsis* genomes

**Figure 2:**
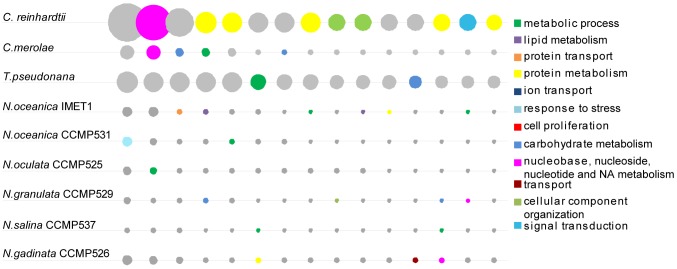
Structure of paralogous groups in each *Nannochloropsis* strain. Each circle represents a paralogous group. The area of the circle is proportional to the size of the paralogous group. The top 15 largest paralogous groups in each of the six *Nannochloropsis* genomes and the other three model microalgae (including *T. pseudonana*, *C. merolae* and *C. reinhardtii*) are shown. The largest paralogous groups in the six *Nannochloropsis* genomes and the other three model microalgae, including *T. pseudonana*, *C. merolae* and *C. reinhardtii*. The color of the circle represents the functions (as defined by the associated GO Slim terms in biological process) encoded by the paralogous group. The paralogous groups in each *Nannochloropsis* strain are relatively small in size.

To seek the cause of the structural divergence among the *Nannochloropsis* genomes, we clustered all encoded proteins based on their sequence similarity. Among sequenced plant and algal genomes, large paralogous groups are common, e.g., 217 F-box family protein genes in *Arabidopsis* [13] and 51 Class III guanylyl and adenylyl cyclase genes in *Chlamydomonas* [7]). However, *Nannochloropsis* spp. appear to have adopted a strategy in which paralogous groups are less biased in size, i.e., they formulate a large number of relatively small paralogous groups (**Figure 2**, **Text S1**). There are 4,263, 4,325, and 7,171 paralogous groups in *Thalassiosira*, *Chlamydomonas*, and *N. oceanica* IMET1, respectively. The top 15 largest paralogous groups in each *Nannochloropsis* genome range in size from two to seven genes, with a median value of three to four (**Figure 2**). For example, the largest paralogous group in IMET1 consists of 11 genes (mainly in metabolic process), which is in a sharp contrast with *T. pseudonana* (46 genes; protein modification process; [8]), *Cyanidioschyzon merlae* (23 genes; DNA metabolic process; [14]) and *C. reinhardtii* (150 genes; protein modification process; [7]). As genes from different origins might exhibit relatively low sequence conservation and thus fail to formulate a paralogous group, the reduced sizes of paralogous groups in the *Nannochloropsis* genomes might result from the integration of multiple genome resources, which is consistent with the proposal that heterokonts originated from multiple secondary endosymbiosis [15]. This observation also suggests that strain-specific gene sequence duplication was relatively rare in *Nannochloropsis*. On the other hand, it is also possible that *Nannochloropsis* spp. have adapted to their environment via a strategy of frugality in proteome structure, with paralogous protein-coding genes either emerging less frequently or many of them being lost.

Among the 8,992 homologous groups from the six *Nannochloropsis* genomes (by OrthoMCL [16]; based on amino acid sequence similarity), 1,731 included the genes from all six strains. However, 2,312, 1,515, 1,551 and 1,653 groups included genes from two, three, four and five of the strains, respectively, and thus were “mosaic” groups as they included genes from only a subset, but not all, of the strains. Furthermore, 230 groups were specific to one of the six strains, with 4 to 151 such groups in each strain. The large number of mosaic paralogous groups (7,031 or 78.2% in total) could explain the large size of the *Nannochloropsis* pan-genome, although the numbers of genes and gene groups could be over- or under-estimated due to the presence of alternative splice forms or artifacts of genome assembly.

### (IV) Gene dose expansion in each *Nannochloropsis* genome at selected steps of lipid synthesis pathways

**Figure 3:**
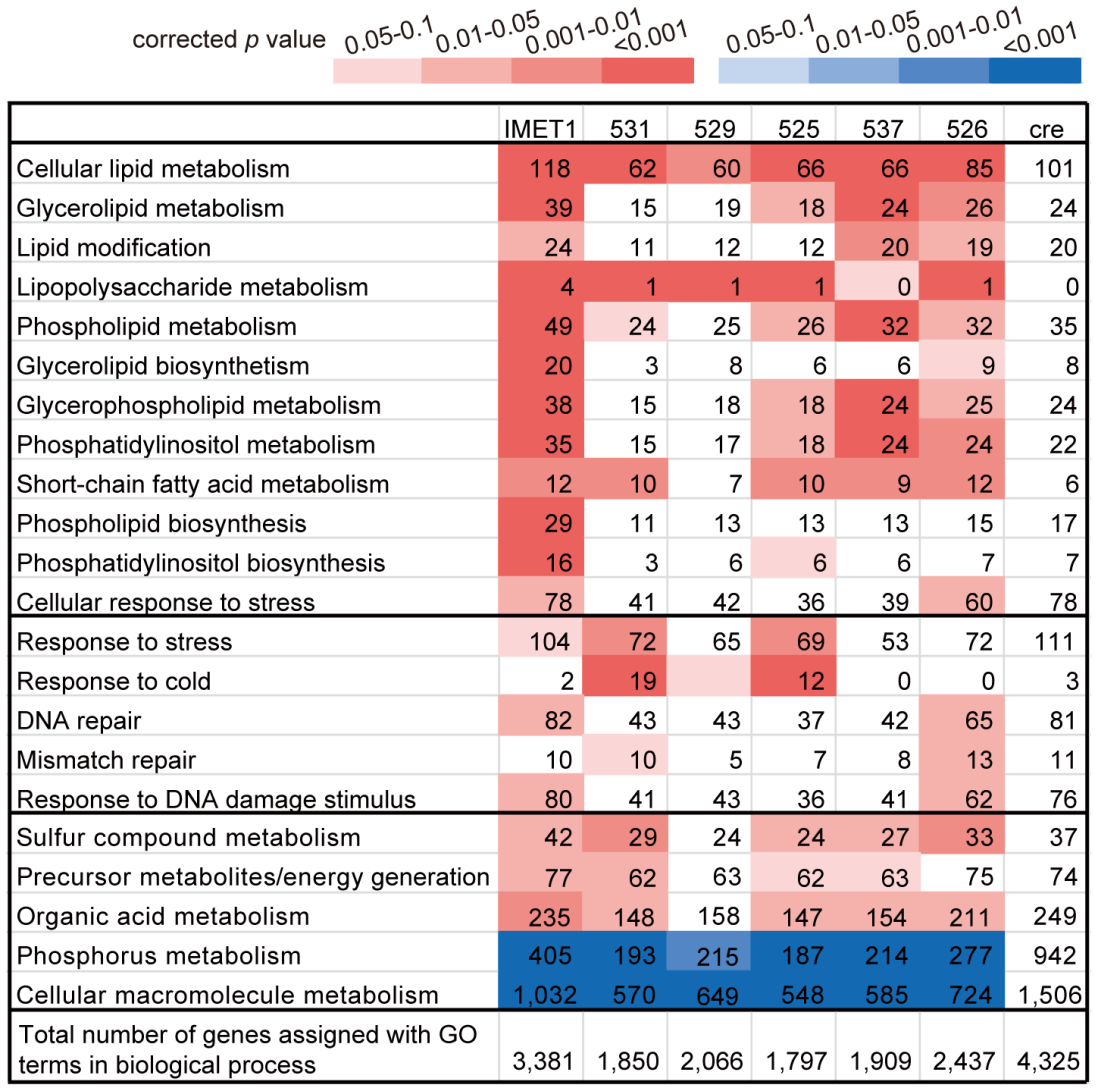
Functional conservation and variation of the *Nannochloropsis* genomes. For each genome, the numbers of genes assigned to each GO term and its subcategory terms are shown. The color scheme, defined by the scale bar on the top, represents the degree of relative enrichment or depletion for each functional category as compared to *C. reinhardtii*. The *p* values of enrichment or depletion were calculated using a binomial test corrected by FDR for multiple comparisons. IMET1, *N. oceanica* IMET1; 531, *N. oceanica* CCMP531; 529, *N. granulata* CCMP529; 525, *N. oculata* CCMP525; 526, *N. gaditana* CCMP526; 537, *N. salina* CCMP537; cre, *C. reinhardtii*.

Despite their high structural diversity, each of the six *Nannochloropsis* genomes exhibits functional features that underlie their oleaginous phenotypes. There is significantly higher gene enrichment for cellular lipid metabolism in each genome than in *C. reinhardtii* (**Figure 3** **, Dataset S2**). In all or most of the *Nannochloropsis* strains, the subcategories of lipid metabolism are enriched, including glycerolipid metabolism, phospholipid metabolism, lipopolysaccharide metabolism and lipid modification. Metabolic pathways enriched in *Nannochloropsis* also include organic acid metabolism, precursor generation and sulfur compound metabolism. Genes related to stress response, including responses to DNA damage stimulus, DNA repair and cold stress response, were also enriched in several *Nannochloropsis* strains. However, the number of genes involved in phosphorus metabolism and cellular macromolecule metabolism was significantly lower in each *Nannochloropsis* strain than in *C. reinhardtii* (**Figure 3**). Thus, the enrichment of gene doses in lipid metabolism pathways and stress response-related pathways appears to be a shared feature of *Nannochloropsis* genomes and likely underlies their advantageous oleaginous and environmental tolerance traits.

**Figure 4:**
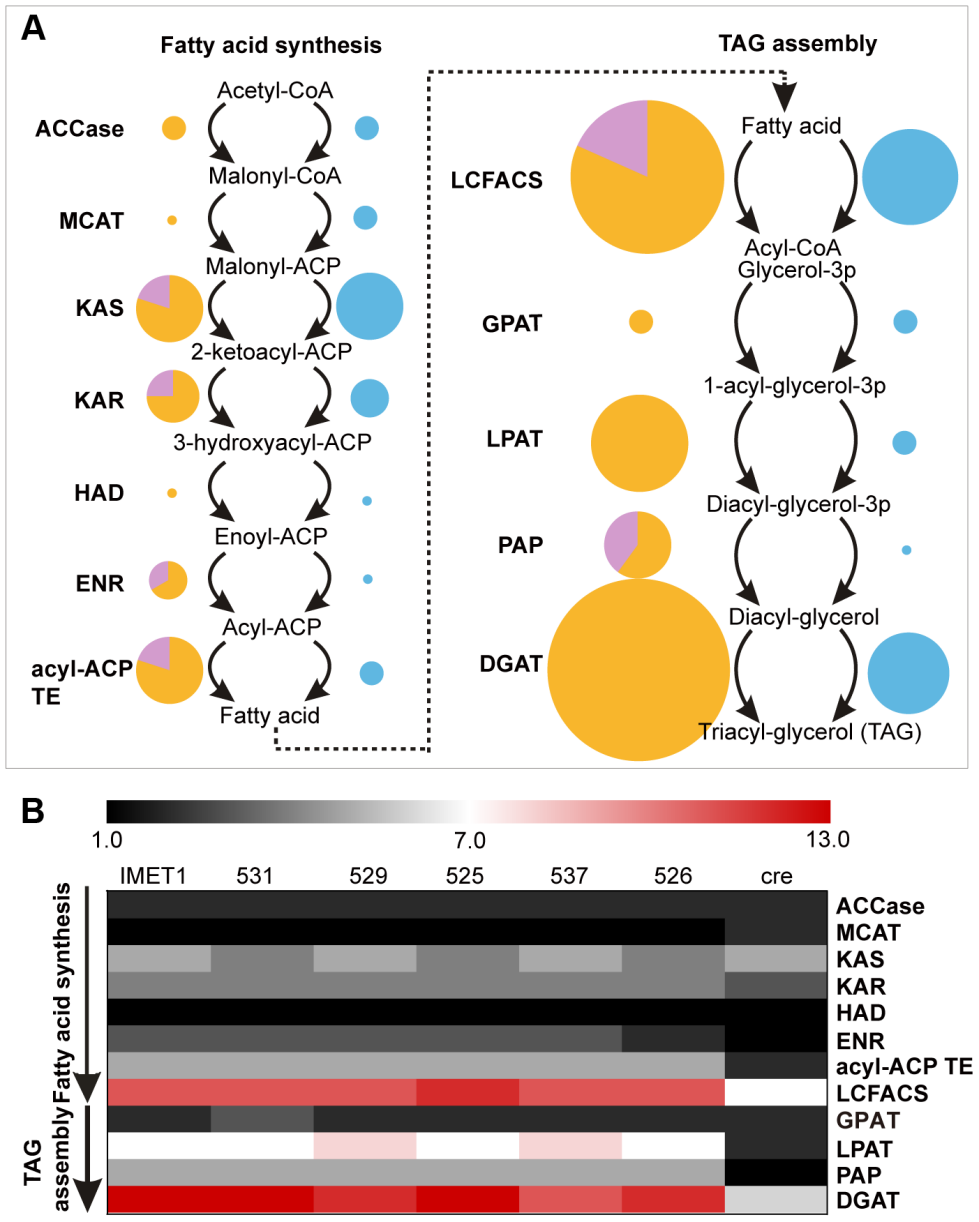
Enrichment of lipid biosynthesis genes in each of the six *Nannochloropsis* strains. (**A**) The gene dose expansion in *N. oceanica* IMET1 as compared to *C. reinhardtii*. In the schema, enzymes in each reaction node in the *Nannochloropsis* and *C. reinhardtii* lipid biosynthesis pathways are represented as yellow and blue circles, respectively. Length of radius stands for gene dose. Putative HGT genes in each node in IMET1 are shown in purple. *Chlamydomonas* genes were not investigated for HGT events here. (**B**) The expansion in gene dose was conserved among the *Nannochloropsis* genomes. Each colored cell in the heatmap represents the gene copy numbers in each of the *Nannochloropsis* strains and in *C. reinhardtii*. The scale of the color bar ranges from 1 (the lowest copy number among the genomes) to 13 (the highest copy number).

In the lipid biosynthesis pathway (the *de novo* biosynthesis of fatty acids and TAG), a prominent expansion in gene copy number in particular reaction nodes was observed as a shared feature among the six *Nannochloropsis* strains, despite a genome size only one-fourth of *C. reinhardtii*. Such enriched genes include those encoding ketoacyl-ACP synthase (KAS, four to five in each *Nannochloropsis* strain vs. three in *C. reinhardtii*), acyl-ACP thioesterase (acyl-ACP TE, five vs. one), long-chain fatty acyl-CoA synthetase (LC-FACS, 11–12 vs. seven), phosphatidic acid phosphatase (PAP, five vs. one), and the last two acyltransferases: lysophosphatidyl acyltransferase (LPAT, seven to eight vs. one) and diacylglycerol acyltransferase (DGAT) (**Figure 4**). Multiple copies of KAS proteins were found in each *Nannochloropsis* strain for the assembly of type II fatty acid synthases. In addition, six bacterial type I fatty acid synthase genes, each with several conserved functional domains, were identified (compared to only one in *C. reinhardtii*); phylogenetic analysis revealed that these genes are closely related to polyketide synthases (**Figure S10**), yet they might be involved in fatty acid synthesis [17].

Notably, such expansion in gene dose was not ubiquitous along the TAG pathway. For many of the nodes, the gene doses are comparable to those in *C. reinhardtii* (**Figure 4B**). These nodes include the acetyl-CoA carboxylase (ACCase), MCAT, KAR, HAD in fatty acid biosynthesis, GPAT in TAG assembly, and other membrane lipid biosynthesis-related enzymes (such as the MGD and DGD in galactolipid synthesis, SQD in sulfolipid synthesis, BtaA and BtaB in betaine lipid synthesis and EPT in phosphatidylethanolamine synthesis). The expansion of gene dose for the selective steps highlights their crucial roles in channeling carbon flux into TAG synthesis and might be considered a “genomic signature” of oleaginousness.

### (V) Evolutionary origin of gene dose expansion in lipid biosynthesis pathway genes

To probe the evolutionary forces expanding the TAG biosynthesis gene repertoire in *Nannochloropsis*, we carried out a phylogenomic analysis to investigate the horizontal gene transfer (HGT) events in *N. oceanica* IMET1 genome (**Text S1**; [18]). We identified 99 HGT candidates (**Figure S11A**; **Dataset S3**), accounting for 1.0% of nuclear genes. Among them, the most abundant functions encoded (in terms of GO Slim terms in biological process) included biosynthetic process, small molecule metabolism, cellular nitrogen compound metabolism and lipid metabolism (**Figure S11**).

HGT appeared to have played an important role in the evolution of oleaginousness loci in these organisms. Totally nine HGT candidates (15.3% of total lipid biosynthesis genes, much higher than average percentage of HGT presence in nuclear genome) were inferred in most of the nodes with increased gene doses, such as KAS, enoyl-ACP reductase (ENR), acyl-ACP TE, LC-FACS and PAP (**Figure 4A** **, Figure S12, Figure S13**). PAP catalyzes the Mg^2+^-dependent dephosphorylation of phosphatidic acid (PA) to yield diacylglycerol (DAG) and P_i_. Both PA (via CDP-DAG) and DAG can enter phospholipid synthesis, and DAG is the direct precursor of TAG. Thus, PAP may control the direction of carbon flux and affect overall cellular lipid synthesis [19]. Five genes encoding PAP enzymes were found in each *Nannochloropsis* strain: three were conserved in eukaryotes, while the other two were clustered with the bacteria, indicating a bacterial HGT origin (either one HGT followed by gene duplication or multiple horizontal transfers; **Figure S12F, Figure S13F**). The two horizontally transferred PAP genes exhibited higher transcriptional levels than the eukaryotic ones. The presence of multiple prokaryotic PAP genes suggests complex mechanisms to regulate the substrate preference for the synthesis of various classes and species of lipids. Among the ENR genes in each *Nannochloropsis* strain, two likely originated by HGT from bacteria into the common ancestor of the six *Nannochloropsis* strains (**Figure S12C**, **Figure S13C**; suggested by the absence of other heterokonts in the bacterial ENR clade), which were then inherited by each of the *Nannochloropsis* strains.

**Figure 5:**
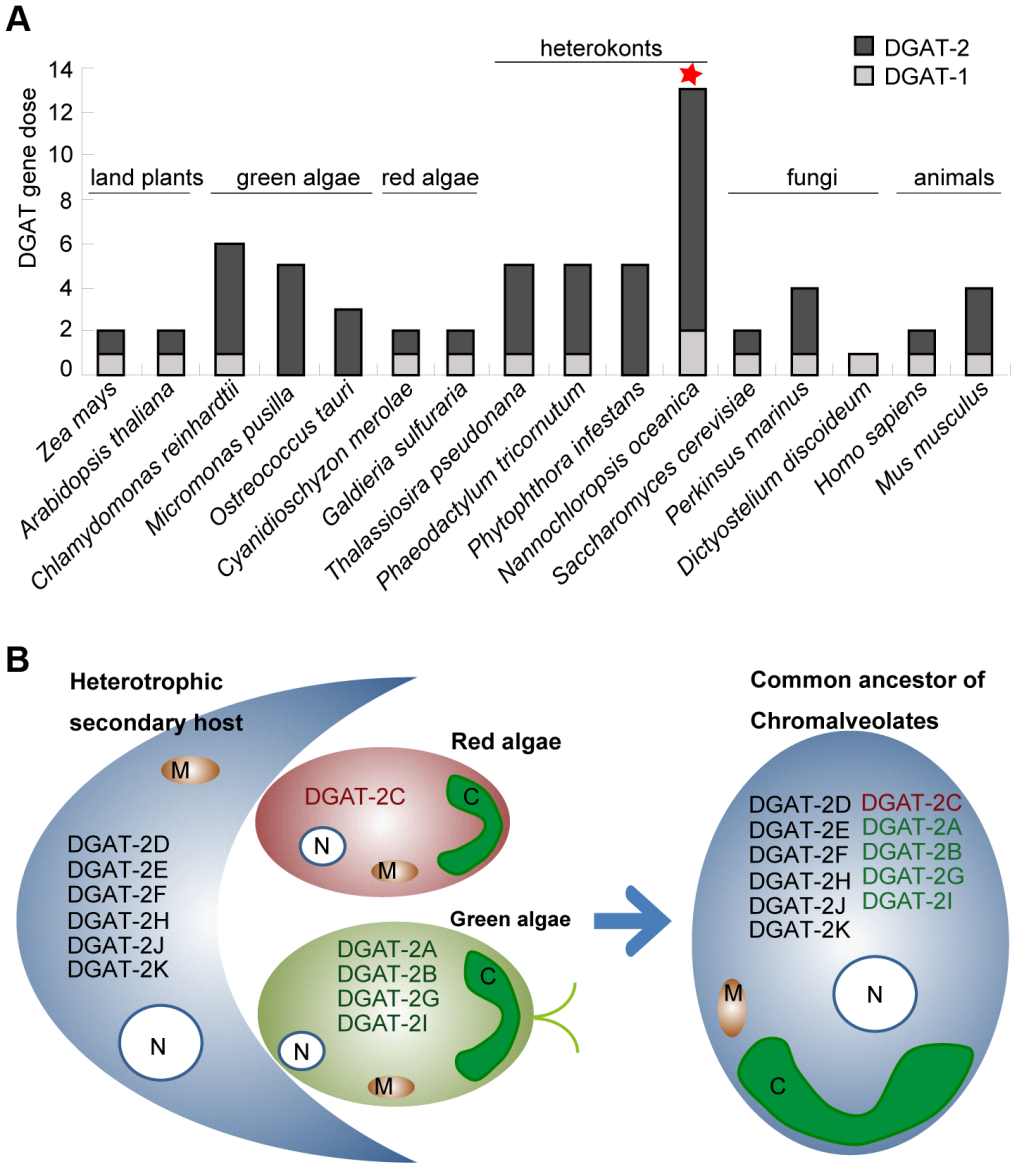
Divergent phylogenetic origins of *Nannochloropsis DGAT-2* genes. (**A**) Gene doses of *DGAT-1* and *DGAT-2* in *Nannochloropsis* and several model organisms. The numbers of *DGAT-1* and *DGAT-2* genes in each genome are indicated by different colors. *N. oceanica* IMET1 is marked by red star. (**B**) Schema illustrating the divergent phylogenetic origins of *Nannochloropsis DGAT-2* from the red/green algae-related endosymbionts and potential secondary host. The protein names listed in each individual schematic cell indicate the DGAT-2 genes that were likely to be encoded in the genome. Abbreviations: N, nucleus; C, chloroplast; M, mitochondria.

The most prominent example of gene dose expansion is DGAT, which catalyzes the last step of TAG synthesis from DAG and acyl-CoA [20] and includes DGAT-1 and DGAT-2 [21]. There are 12–13 *DGAT* in each *Nannochloropsis* strain (one to two *DGAT-1* and 11 *DGAT-2*), representing the highest dose among known genomes (**Figure 5A**). In contrast, only six and four *DGAT* are present in *C. reinhardtii* and the diatom *T. thalassiosira*, respectively, and even fewer in some other green algae and heterokonts (**Figure 5A**). In IMET1, all the *DGAT-1* and *DGAT-2* were transcriptionally active [FPKM (Fragments Per Kilobase of exon per Million mapped reads) >1.0]. Phylogenetic analysis of *DGAT* from selected bacteria, fungi, algae and higher plants revealed extraordinary evolutionary diversities of all 74 *DGAT* in the six *Nannochloropsis* strains (**Figure S14**). Several observations were apparent. (*i*) The partition of *DGAT-1* and *DGAT-2* might have occurred early, likely before the primary endosymbiosis event or even earlier. (*ii*) The copy number of *DGAT-1* was lower (1–2) and less variable than that of *DGAT-2*, which is consistent in a wide range of organisms from bacteria to land plants. (*iii*) A similar degree of *DGAT-2* dose expansion was observed in all six *Nannochloropsis* strains (**Figure 4B**). Moreover, for each of the 11 *DGAT-2* identified in each strain, the orthologs in the other five strains were all identified and clustered into a phylogenetic group (**Figure S14**); the sequence identity between orthologous gene pairs was >98% between the two *N. oceanica* strains, >80% among *N. oceanica*, *N. oculata* and *N. granulate*, and >65% between *N. oceanica* IMET1 and the outmost *N. gaditana*. These results suggested the stable inheritance of *DGAT-2* genes in *Nannochloropsis* evolution. In contrast, *DGAT-1* might have experienced species-specific gene loss. For example, no counterparts of *DGAT-1B* in IMET1 were found in *N. salina* and *N. gaditana* despite a high degree of conservation of this gene in the other four strains. (*iv*) The 11 *DGAT-2* genes in IMET1 exhibited relatively low intra-genome pairwise identity (averaging 18%), and each was grouped into a separate paralogous group with its orthologs from the other five *Nannochloropsis* strains, indicative of distinct and divergent phylogenetic origins of *DGAT-2* in *Nannochloropsis*. Two of the *DGAT-2* genes in IMET1 (*DGAT-2F* and *DGAT-2D*) exhibited a relatively high protein sequence similarity (identity at 51%), suggesting that the two genes might be derived from a gene duplication event in the *Nannochloropsis* lineage (**Figure S14**). However this individual case of suspected gene duplication cannot account for the expanded dose of *DGAT-2* genes in IMET1.

To infer the origin of these genes, a comprehensive phylogenetic analysis was carried out among all species with genomes and ESTs available in several public databases (**Text S1**; [22]). *DGAT-2C* showed a phylogeny with strong affiliation with the red algae *C. merolae* and formed a sister group with those from other chromalveolates (**Figure S15A, Figure S16A**). The most plausible explanation for such strong links between *Nannochloropsis* and red algal *DGAT* is a red-algae derivation of *DGAT-2C* through endosymbiotic gene transfer (EGT) in the secondary endosymbiosis event (which was proposed as the evolutionary mechanism through which the common ancestor of chromalveolates acquired chloroplasts from a red algae-related endosymbiont [23]). On the other hand, four *DGAT-2* genes, including *DGAT-2A*, *DGAT-2B*, *DGAT-2I* and *DGAT-2G*, are clustered with their counterparts from green algae as well as other chromalveolates (**Figure S15B–E, Figure S16B–E**), suggesting a green algal origin of these *DGAT-2* genes. This is consistent with the hypothesis of a green algae related endosymbiont residing in the common ancestor of chromalveolates [15]. Three (*DGAT-2A*, *DGAT-2I* and *DGAT-2C*) of the above five red-lineage (red algae derived) and green-lineage (green algae derived) *DGAT-2* genes were predicted to harbor chloroplast targeting signals, supporting their ancestral derivation from the endosymbionts. The higher dose of green- than red-lineage *DGAT-2* in each of the *Nannochloropsis* strains suggests a more significant contribution of the green lineage to the oleaginous traits of modern *Nannochloropsis*. Furthermore, phylogenetic trees of the other six DGAT-2 genes did not exhibit unambiguous relationships with those from red or green algae and are thus referred to as “unresolved.” It is possible that several of these genes originated from the secondary host [24], as four (*DGAT-2D*, *DGAT-2E*, *DGAT-2F* and *DGAT-2H*) of the six genes were predicted to be located in the endoplasmic reticulum (ER) or cytosol. Thus, the observed sequence divergence of the 74 *DGAT* genes (eight type I and 66 type II) in the six *Nannochloropsis* genomes mainly resulted from their diverse origins from the red- or green-algae–related endosymbionts (through EGT) and the secondary host (**Figure 5B**).

Phylogenetic evidence also supports a green endosymbiont origin for one gene encoding MCAT (s00247.g6828) in fatty acid biosynthesis (**Figure S15F, Figure S16F**). Thus, the diverse evolutionary origin of the *Nannochloropsis DGAT-2*s and one of the other lipid synthesis genes has underlain their massive genetic pools and likely contributed to the extraordinary capacity for TAG synthesis in present-day strains.

In addition to their diverse origins, differentiation in selective pressure appeared to underlie the sequence divergence of *DGAT* and other members of lipid-related gene families. *DGAT* genes in *Nannochloropsis* were generally under strong purifying selective pressure (K_a_/K_s_ typically under 0.1). However, higher K_a_/K_s_ ratios were observed in the red-lineage *DGAT-2C* of 0.11 (**Figure S14**). No significant difference in the ratio was found between the green-lineage and secondary-host–originated *DGAT-2*. Furthermore, *DGAT-2C* with the highest K_a_/K_s_ ratio was among the *DGAT-2* genes with the lowest transcriptional level under normal growing conditions, while *DGAT-2J* with the lowest ratio was one of the most transcribed *DGAT-2* (second only to *DGAT-2A*). These findings add further support to the negative correlation between transcriptional level and selective pressure in the evolution of *Nannochloropsis* genes.

### (VI) Enrichment of glycoside hydrolase genes and their evolutionary origin

Dramatic enrichment of glycoside hydrolase (GH) genes accompanied by a reduced pool of glycoside synthase genes (as compared to *C. reinhardtii*) was also observed in each of the *Nannochloropsis* genomes. *C. reinhardtii* harbors seven starch synthase genes for starch production and two 1,3-β-glucan synthase genes; however, each *Nannochloropsis* encodes just one 1,3-β-glucan synthase gene (which might convert glucose into the polysaccharide chrysolaminarin or laminarin), and no starch synthase genes were found. Conversely, 48–49 GH genes were found in each strain (**Dataset S4**), with a gene dose per Mb of genome 6–7 fold higher than that of *C. reinhardtii* (27 GH genes). These *Nannochloropsis* genes were from 13 different GH families, dominated by GH2 (13 members), GH9, GH3 and GH1 families with over four members. Surprisingly, there were only three genes in the GH16 family, which specifically hydrolyzes the glycosidic bond of 1,3-β-glucan, while GH16 was the dominant group in *C. reinhardtii*, with five members. In IMET1, 91.7% of the 48 GH genes were transcriptionally active at each of the time points under both N-replete and N-depleted culture conditions (FPKM>1.0; **Text S1**). Among them, 16 exhibited significant variations at the transcriptional level under N-depleted conditions (10 with increased transcription), including two members of the GH2_C family and one GH17 gene with a significant increase in transcription (fold-change >1.5) from 3 h and 6 h after the onset of N-depletion and one GH9 gene down-regulated under the same conditions. The monosaccharides released from GH that catalyzed hydrolysis of polysaccharides may be used in glycolysis to produce acetyl-CoA and ATP for fatty acid synthesis.

Among the 48 GH genes in each strain that were conserved among the six genomes, 16 were inherited from the common ancestor of heterokonts, as their homologs were found in the diatoms *Phytophthora* and *Ectocarpus*. Another five GH genes were likely to have originated from bacteria via HGT. Among these, three GH8 genes inferred to be horizontally acquired from cellulose-digesting *Clostridium*-like bacterium were absent in other sequenced unicellular algae. The remaining 27 GH genes were phylogenetically closest to homologs in animals, insects or multicellular fungi, such as the nine putative cellulase genes that were most similar to those in the nematode *Pristionchus*, indicating HGT events with donors being *Nannochloropsis*-like organisms [25]. In addition, *N. granulata* and *N. salina* each possessed one strain-specific GH gene that might have been introduced after their speciation.

## Discussion

Microalgae, which are primarily unicellular, aquatic and photosynthetic eukaryotes, are phylogenetically diverse. They are responsible for over 45% of our planet's annual net primary biomass [26]. The *Nannochloropsis* genomes studied here, one of the first such datasets for microalgae, reveal the nature and degree of genome divergence and dynamics at the strain, species and genus level. They could serve as an initial framework for genome-wide association studies, while the genome-derived nuclear gene markers should be useful for highly sensitive typing of strains.

The genomes of the six oleaginous *Nannochloropsis* strains presented here are of relatively small size and high coding potential and many fewer mobile elements compared to many previously sequenced microalgae [9]. The large size of the *Nannochloropsis* pan-genome can be partially traced to the large number of mosaic paralogous groups, which further suggests a significant degree of species-specific gene loss during *Nannochloropsis* evolution. On the other hand, the small core genome size and the large number of mosaic homologous gene clusters among the *Nannochloropsis* spp. suggest that, as one moves down the tree of life for stramenopiles, the number of shared genes reduces quickly and is replaced by lineage-specific gene gains and losses. The core genes generally exhibit lower K_a_/K_s_ ratio but higher transcriptional levels than non-core genes, suggesting their roles in shaping the evolution of microalgal genes. Our findings, together with observations in yeasts [10], [27], revealed a link that is conserved in unicellular eukaryotes in terms of gene function, selective pressure, transcriptional level and gene essentiality.

Despite the high sequence diversity of protein-coding genes, the six *Nannochloropsis* genomes shared a genus-level oleaginousness signature that included enrichment of selective lipid biosynthesis genes and certain glycoside hydrolases that potentially shift carbon flux from storage carbohydrate to TAG synthesis. It is quite remarkable that these gene expansions have occurred despite their significant genome shrinkage relative to other microalgae such as *C. reinhardtii*. Different mechanisms have underlain the emergence of the signature. Multiple-genome pooling was particularly evident for the 11 *DGAT-2* in each strain, which were all transcriptionally expressed during TAG synthesis and apparently originated from at least three ancient genomes: (*i*) the engulfed green algae, (*ii*) the engulfed red algae and (*iii*) the host cell in the secondary endosymbiosis.

Chromalveolates include both photosynthetic (e.g. diatoms and Eustigmatophyceae that include *Nannochloropsis*) and non-photosynthetic taxa (e.g., *Phytophthora*). The chromalveolate hypothesis suggests that the common ancestor of Chromalveolates originated via an eukaryotic host (i.e., the secondary host) engulfing a red alga (as the secondary plastid) in an ancient secondary endosymbiosis event [23]. The presence of a large number of “green genes” in the diatom nuclear genomes has been interpreted as evidence of a cryptic prasinophyte-like secondary endosymbiosis before the red algae intake [15]. Though confounded by potential sampling bias against red algae and artifacts in phylogenetic analysis [28], this hypothesis was supported by the 172 membrane transporter genes showing potential origins from green or red algae in a relatively strict phylogenomic analysis [22]. Moreover, genomes of the cryptophyte alga *Guillardia theta* and the chlorarachniophyte alga *Bigelowiella natans* also contain hundreds of genes with a phylogenetic affiliation to red or green algae [24]. Our search of *DGAT-2* in publicly available red algae genomes (and ESTs) returned one *DGAT-2* each from *Cyanidioschyzon merolae*, *Galdieria sulphuraria* [18] and *Porphyridium purpureum* [29]. The paucity of *DGAT-2* in red algal genomes and the distinct features of these genes in the six *Nannochloropsis* genomes (the greatly expanded copy number, large pair-wise sequence divergence, rare gene duplication events, and absence of mobile elements or evidence for HGT in each of the *DGAT-2* loci) suggested multiple-genome pooling as the cause for the massive *DGAT* pool in *Nannochloropsis* spp. These findings also provided additional support for the existence of a green algae–associated secondary endosymbiosis in the evolutionary history of chromalveolates.

Furthermore, among the six *Nannochloropsis* strains, the inheritance of each *DGAT-2* was highly conserved in that no strain-specific duplications or losses were found for any *DGAT-2* in each of the six strains, and the genes have been under strong negative selection. In contrast, diatoms such as *T. pseudonana* (believed to have also experienced the multiple secondary endosymbiosis [15]) encode many fewer *DGAT-2*; only four *DGAT-2* were identified, and all were predicted to be from the green algae–related endosymbiont and the secondary host, with none from red lineage. The absence of *Thalassiosira* genes in certain gene-phylogeny clusters (e.g., the red-lineage *DGAT-2C*) in the diatom, in contrast to the presence of these genes in *Nannochloropsis* and many other heterokonts, suggests the loss of *DGAT-2* in diatoms. Thus, such strict inheritance and stable maintenance of the large reservoir of *DGAT-2* from multiple lineages seem to be *Nannochloropsis*-specific. It also suggests the essentiality of each *DGAT-2* and its possible functional complementarity in the cell.

In addition, HGT primarily from bacteria were found in the majority of the gene dose-expanded lipid synthesis nodes and in many glycoside hydrolases. In the red alga *Galdieria sulphuraria*, 5% of protein-coding genes were acquired from bacteria and archaea via HGT, which forged its adaptation to a thermophilic and metal-rich environment [18]. The HGT events in *Nannochloropsis* likely reflected an organismal adaptation to a niche that favored oleaginousness and glycoside hydrolysis.

Therefore, the multiple-genome pooling and horizontal genetic exchange from bacteria, together with the selective inheritance of lipid synthesis genes and species-specific gene loss, might have underlain the enormous genetic apparatus for oleaginousness and led to the structural divergence and functional conservation observed among present-day *Nannochloropsis*. In many organisms, other mechanisms such as gene and genome duplications may play an important role in supplying new genetic materials for organismal adaptation [30] and have been frequently proposed as drivers of the emergence of particular traits in bacteria [31], [32], fungi [33], [34], plants [35] and animals [36]. Thus, the extraordinary origin and evolution of oleaginous traits in *Nannochloropsis* have important implications in the selection and genetic engineering of such traits in these and other microalgae of economic interest.

## Methods

### Data files

All genomic data for this study, including the assembled genomes and mRNA-Seq data, were deposited at NCBI. The BioProject accessions for assembled genomes were: PRJNA202418 for *N. oceanica* IMET1, PRJNA65107 for *N. oculata* CCMP525, PRJNA65111 for *N. granulata* CCMP529, PRJNA65113 for *N. oceanica* CCMP531 and PRJNA62503 for *N. salina* CCMP537. The mRNA-Seq data were deposited at SRA under SRP032930.

### Sequencing of the *Nannochloropsis* genomes and the transcriptomes of *N. oceanica* IMET1

Five new *Nannochloropsis* genomes were sequenced in this work (**Table 1**; **Table S1**). For *Nannochloropsis oceanica* IMET1, both shotgun sequencing data and paired-end data with different pair distances from 454 Titanium and Illumina GAIIx were collected. Newbler (Roche) was used for initial assembly. Gap-filling and scaffold-building were performed with Illumina data, followed by manual manipulation and sorting of contigs. Genes were predicted by combining the *ab initio* predictions with predictions based on mRNA-Seq read alignments (387K aligned cDNA reads from a Roche 454 Sequencer) by AUGUSTUS (v2.5). For each of the other four *Nannochloropsis* strains (Table S1), paired GAIIx reads were assembled using Velvet with specified insert sizes. The previously published genome sequence of *N. gaditana* CCMP526 [5] was downloaded from http://Nannochloropsis.genomeprojectsolutions-databases.com/. Gene models of each of the six genomes were predicted using two different *ab initio* gene predictors (AUGUSTUS and GeneID) followed by a combination of gene models using EVidenceModeler (EVM) with a 1∶1 weight ratio. For all strains, predicted protein-coding genes were annotated via searching against three databases: the NCBI NR and KEGG databases by BlastP, and the Gene Ontology database by InterProScan. GO terms were mapped to the GO slim hierarchy proposed by the GO consortium by a customized script (http://www.bioenergychina.org/fg/d.wang_scripts/).

For collecting the transcriptomics datasets underlying TAG production, *N. oceanica* IMET1 was cultivated in f/2 liquid medium [37] with 4 mM NO_3_^−^ under continuous light at 50 µmol photons m^−2^ s^−1^. Mid-logarithmic phase algal cells were inoculated in nitrogen-replete and nitrogen-depleted conditions, respectively. Total RNA were collected at 3, 6 and 24 h after each inoculation and pooled together for full-length cDNA sequencing in 454 Titanium. The data produced were subsequently used for gene prediction. Furthermore, total RNA from each of the aforementioned control (nitrogen-replete) and nitrogen-starvation conditions along the time points of 3, 6 and 24 h after the onset of nitrogen depletion (six samples under each condition) were loaded for mRNA-Seq in Illumina GAIIx.

### Phylogeny analysis

*Nannochloropsis* core genes were identified as the intersections of the five “IMET1 pairwise cores,” which were obtained by searching IMET1 proteins via BlastP and tBlastN against the proteome and the genome, respectively, of each of the other five strains with an e-value cutoff of 1e-5 and a protein sequence identity cutoff of 80%. Paralogous groups among these six strains were identified by a Markov Clustering algorithm (OrthoMCL [16], v. 4) with an inflation index of 1.5. PAML (v. 4.4c) codon substitution models and likelihood ratio tests (codeml) were used to estimate the selective pressure. An identical method was applied in the establishment of paralogous groups among other model microalgae.

HGT candidates were inferred following the method in the genomic analysis of *Galdieria sulphuraria* (**Text S1**; [18]). Phylogenetic trees for each of the putative HGT genes in NEWICK format were deposited in **Dataset S3**. The phylogenetic tree for each HGT candidate was manually checked and only accepted when a clear pattern of HGT was observed in both Neighbor Joining (NJ) and Maximum Likelihood (ML) trees. To deduce the evolutionary origins of lipid biosynthesis-related genes, we first implemented the strategy described in Chan et al. [22] to build a comprehensive database and to construct the homologous groups for each lipid synthesis gene, except that we collected more recently published genomes and EST datasets updated in public databases, including genomes of the red algae *G. sulphuraria* [18], *Chondrus crispus* [38] and *Porphyridium purpureum* [29]. In the following phylogenetic analysis, phylogenies for the homologous group of each lipid synthesis gene were constructed in MEGA5 by both NJ and ML methods. A gene was inferred to be potentially derived from a green or red algae related secondary endosymbiont when the phylogeny was supported by both NJ and ML trees.

For a comprehensive and detailed description of the methods, please refer to **Text S1**.

## Supporting Information

Dataset S1 Candidate nuclear-genome phylogenetic markers in *Nannochloropsis*.
(XLS)

Dataset S2 Genes from Figure 3 annotated with Gene Ontology terms in biological processes. Genes from each *Nannochloropsis* strain and *C. reinhardtii* are listed respectively in an accompanying Excel worksheet.
(XLS)

Dataset S3 Putative horizontally transferred genes in the *N. oceanica* IMET1 genome. Each line represents a HGT candidate. Both NJ and ML trees in NEWICK format for each HGT candidate are included in the dataset. Species names are shown at the end of each branch. Characters in parentheses represent the classification of each species: B, bacteria; B_cyano, cyanobacteria; E, eukaryotes; E_M, metazoan; E_P, plant; E_p_a, eukaryotic algae; E_F, fungi. HGT candidates related to the lipid biosynthesis pathway (as noted in **Figure 4** and **Figure S12**) are highlighted.
(XLS)

Dataset S4 Glycoside hydrolase genes encoded in the *N. oceanica* IMET1 genome.
(XLS)

Figure S1 Total lipid content of the six *Nannochloropsis* strains under normal growth conditions. Information for *N. gaditan*a CCMP526 was cited from Radakovits et al. [5].
(PDF)

Figure S2 Phylogenetic relationship of *Nannochloropsis* and other microalgal lineages. (**A**) Phylogeny based on 18S ribosomal DNA sequences. Higher plants were used as an outgroup. *Nannochloropsis* strains included in this phylogenomic analysis were underlined. (**B**) Phylogeny based on orthologs derived from whole-genome comparisons among *Nannochloropsis oceanica* IMET1, *Thalassiosira pseudonana*, *Cyanidioschyzon merolae*, *Chlamydomonas reinhardtii* and *Arabidopsis thaliana*.
(PDF)

Figure S3 Pulsed-field gel electrophoresis (PFGE) analysis of *Nannochloropsis oceanica* IMET1 chromosomal DNA. Asterisks (*) indicate those bands that likely represent two or more chromosomes. Three DNA size standards that include *Saccharomyces cerevisiae* (240–2,200 Kb, Marker A), *Hansenula wingei* (1–3.1 Mb, Marker B), *Schizosaccharomyces pombe* (3.5–5.7 Mb, Marker C) were used to estimate chromosome sizes. (**A**) PFGE profile of IMET1 chromosomes. The band profile as analyzed by ImageJ is shown on the right. (**B**) PFGE profile of chromosomes larger than 2 Mb.
(PDF)

Figure S4 Functional categories of *Nannochloropsis* core genes. (**A**) GO Slim categories in “molecular function”. (**B**) GO Slim categories in “cellular component”.
(PDF)

Figure S5 Metabolic conservation and divergence among the six *Nannochloropsis* strains. Metabolic pathways were represented in IPATH scheme tools. Red edges indicate KEGG IDs identified in both *Nannochloropsis* core genes and accessory genes. Blue edges indicate those exclusively identified in core genes. Green edges indicate those exclusively identified in accessory genes.
(PDF)

Figure S6 Functional categories of strain-specific genes in *N. oceanica* IMET1. The GO categories (in biological process) of strain-specific genes in each strain were presented.
(PDF)

Figure S7 K_a_/K_s_ of core and accessory gene sets and the link to their transcriptional level in *N. oceanica*. For each of the five-strain orthologous gene sets (excluding the outmost *N. gaditana*) from the *Nannochloropsis* core and accessory genes, PAML model M0 was used to estimate a single ω (K_a_/K_s_, ratio of non-synonymous to synonymous nucleotide divergence) as a measure of the selective pressure that the gene sets were under. (**A**) Different K_a_/K_s_ of core and accessory proteins. (**B**) Selective pressure on the functional categories associated with *Nannochloropsis* core and accessory genes. GO slim terms in biological process with at least three genes associated are shown. Yellow box-plot indicates smallest observation (sample minimum), lower quartile (Q1), upper quartile (Q3), largest observation (sample maximum) and outlier(s) of ω values in a functional category. (**C**) Negative correlation between the K_a_/K_s_ and the transcriptional level of a gene. (**D**) Different transcriptional levels of core and accessory genes.
(PDF)

Figure S8 Selective pressure on protein-coding genes in *Nannochloropsis* spp. For each of the 1,085 six-way single-copy orthologous gene sets, PAML model M0 was used to estimate a single ω (K_a_/K_s_, ratio of non-synonymous to synonymous nucleotide divergence) that is fixed across the reconstructed whole-genome phylogeny. The associated GO slim terms that have at least five genes are shown for (**A**) Biological Process, (**B**) Molecular Function and (**C**) Cellular Component. The red dots represent the median ω in a functional category. The yellow box-plot shows the smallest observation (sample minimum), lower quartile (Q1), upper quartile (Q3), largest observation (sample maximum) and outlier(s) of ω values in a functional category.
(PDF)

Figure S9 Conservation of IMET1 genes in the other five *Nannochloropsis* strains. (**A**) A histogram of the number of IMET1 genes conserved in a series of strain sets. The diagonal in the two-strain sets represents genes that were exclusively conserved in *N. oceanica* species; the diagonal in the four-strain sets represents genes that were exclusively conserved in *N. oceanica* (IMET1 and CCMP531), *N. granulata* and *N. oculata*. (**B**) Functional categories in the GO slim hierarchy of genes that were exclusively conserved in *N. oceanica*, *N. granulata* and *N. oculata*. Categories with percentages less than 1% are pooled and presented as “Others.”
(PDF)

Figure S10 Phylogeny of type I fatty acid synthase. Multiple sequence alignments among six putative PKS proteins from IMET1 (indicated by a red square), as well as type I PKS proteins and type I fatty acid synthases (FAS) from bacteria, as indicated by the species name, were generated with ClustalW. The phylogenetic tree was constructed using the neighbor-joining method in MEGA5 with a bootstrap test (based on 100 replicates).
(PDF)

Figure S11 Functional categories of horizontally transferred genes in *N. oceanica* IMET1. (**A**) The GO Slim categories (in biological process) of the HGT genes in IMET1. (**B**) The GO Slim categories (in molecular function) of the HGT genes in IMET1.
(PDF)

Figure S12 Phylogenies by the NJ method for lipid synthesis genes that were inferred to have originated from HGT. MUSCLE was used to perform multiple alignments for each of the lipid synthesis genes (indicated by a red star) and their orthologous genes generated by Inparanoid Program. Neighbor-joining trees were constructed in MEGA5 with the bootstrapping method (based on 100 replicates), using the passion correction model to calculate evolutionary distances. Branches of bacterial sequences (with the exception of cyanobacteria) are highlighted in red. (**A**) Phylogeny of KAS gene (ID: s00303.g8736) gene; (**B**) phylogeny of KAR gene (s00043.g2007); (**C**) phylogeny of ENR genes (s00007.g154, s00295.g8627); (**D**) phylogeny of the acyl-ACP TE gene (s00355.g10346); (**E**) phylogeny of LCFACS genes (s00262.g7492, scaffold00341.g9817); (**F**) phylogeny of PAP genes (s00058.g2171, s00127.g4338).
(PDF)

Figure S13 Phylogenies by the ML method for lipid synthesis genes that were inferred to have originated from HGT. MUSCLE was used to perform multiple alignments for each of the lipid synthesis genes (indicated by a red star) and their orthologous genes generated by Inparanoid Program. ML trees were constructed in PhyML program by the best protein evolution model selected by ProtTest, using 100 bootstrapping replicates. Branches of bacterial sequences (with the exception of cyanobacteria) are highlighted in red. (**A**) Phylogeny of KAS gene (ID: s00303.g8736) gene; (**B**) phylogeny of KAR gene (s00043.g2007); (**C**) phylogeny of ENR genes (s00007.g154, s00295.g8627); (**D**) phylogeny of the acyl-ACP TE gene (s00355.g10346); (**E**) phylogeny of LCFACS genes (s00262.g7492, scaffold00341.g9817); (**F**) phylogeny of PAP genes (s00058.g2171, s00127.g4338).
(PDF)

Figure S14 Phylogeny of *DGAT* genes in the six *Nannochloropsis* genomes. DGAT genes from all the six *Nannochloropsis* strains, as well as lineages representing bacteria, fungi, microalgae and plants, were aligned in ClustalW. A phylogenetic tree was constructed using the neighbor-joining method in MEGA5 with a bootstrap test (based on 100 replicates). Orthologs (from the six *Nannochloropsis* strains) of each *DGAT* in IMET1 are indicated with the same color. The K_a_/K_s_ value of each orthologous group of *DGAT-2* in *Nannochloropsis* is shown next to the Group ID.
(PDF)

Figure S15 Phylogenies by the NJ method for lipid synthesis genes that were inferred to have originated from secondary endosymbionts. *DGAT-2* from *N. oceanica* IMET1 are indicated by red stars. (**A**) Phylogeny of *DGAT-2C*; (**B**) phylogeny of *DGAT-2B*; (**C**) phylogeny of *DGAT-2A*; (**D**) phylogeny of *DGAT-2I*; (**E**) phylogeny of *DGAT-2G*; (**F**) phylogeny of *MCAT*.
(PDF)

Figure S16 Phylogenies by the ML method for lipid synthesis genes that were inferred to have originated from secondary endosymbionts. *DGAT-2* from *N. oceanica* IMET1 are indicated by red stars. (**A**) Phylogeny of *DGAT-2C*; (**B**) phylogeny of *DGAT-2B*; (**C**) phylogeny of *DGAT-2A*; (**D**) phylogeny of *DGAT-2I*; (**E**) phylogeny of *DGAT-2G*; (**F**) phylogeny of *MCAT*.
(PDF)

Table S1 Genomic and transcriptomic datasets for the six *Nannochloropsis* species and strains. (**A**) Genomic DNA sequencing data for *N. oceanica* IMET1. (**B**) Genomic DNA sequencing data for the other six *Nannochloropsis* strains. (**C**) cDNA sequencing data for *N. oceanica* IMET1. The cDNA sequencing was performed on 454 Titanium. (**D**) mRNA-Seq data for *N. oceanica* IMET1. SG, shotgun; PE, pair-end; MP, mate-pair. *: Illumina GAIIx–based transcriptome sequencing was performed on total mRNA samples isolated from microalgal cells under both the control growth conditions (indicated as “**C**”) and the N-depleted conditions (indicated as “**N**”) at three different time points (3, 6 and 24 h) for each condition.
(DOC)

Table S2 Comparing the genomic features of *N. oceanica* IMET1 and five unicellular eukaryotic microalgae. Parameters of the genomes other than IMET1 were retrieved from the literature.
(DOC)

Text S1 Detailed descriptions of materials and methods.
(DOC)

Text S2 The selective pressure that drives genome diversity in *Nannochloropsis* spp.
(DOC)
