# Supplementary figures and images for: Nannochloropsis Genomes Reveal Evolution of Microalgal Oleaginous Traits
Source: PLoS Genet. 2014 Jan 9;10(1):e1004094. doi: 10.1371/journal.pgen.1004094 (PMC3886936; doi:10.1371/journal.pgen.1004094)

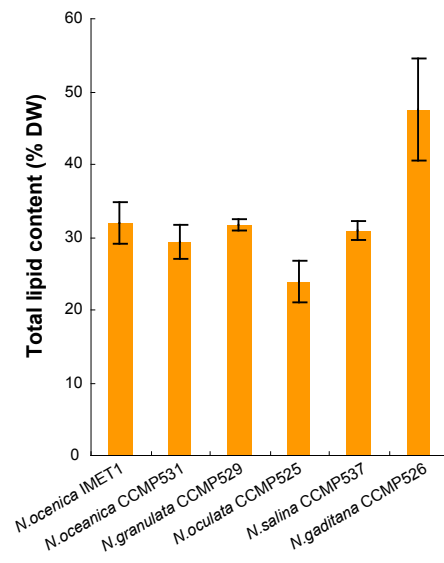

Supplement: Figure S1 — Total lipid content of the six Nannochloropsis strains under normal growth conditions. Information for N. gaditana CCMP526 was cited from Radakovits et al. [5]. (PDF) [file pgen.1004094.s005.pdf]

A

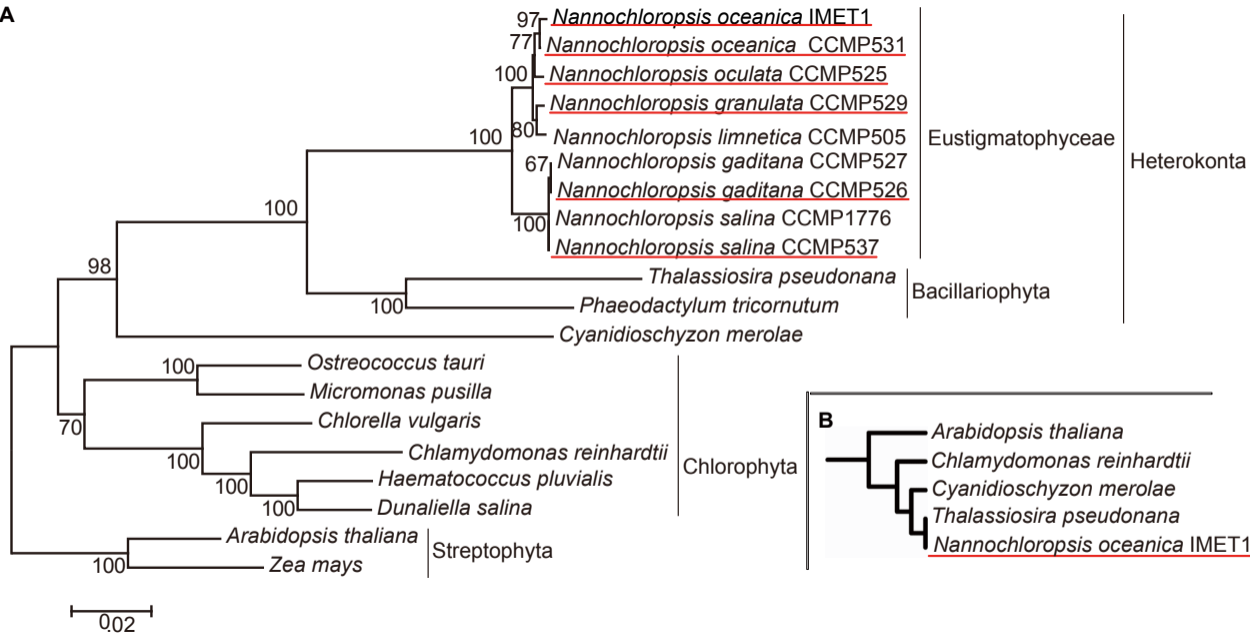

B

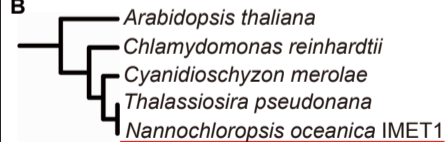

Supplement: Figure S2 — Phylogenetic relationship of Nannochloropsis and other microalgal lineages. (A) Phylogeny based on 18S ribosomal DNA sequences. Higher plants were used as an outgroup. Nannochloropsis strains included in this phylogenomic analysis were underlined. (B) Phylogeny based on orthologs derived from whole-genome comparisons among Nannochloropsis oceanica IMET1, Thalassiosira pseudonana, Cyanidioschyzon merolae, Chlamydomonas reinhardtii and Arabidopsis thaliana. (PDF) [file pgen.1004094.s006.pdf]

**A**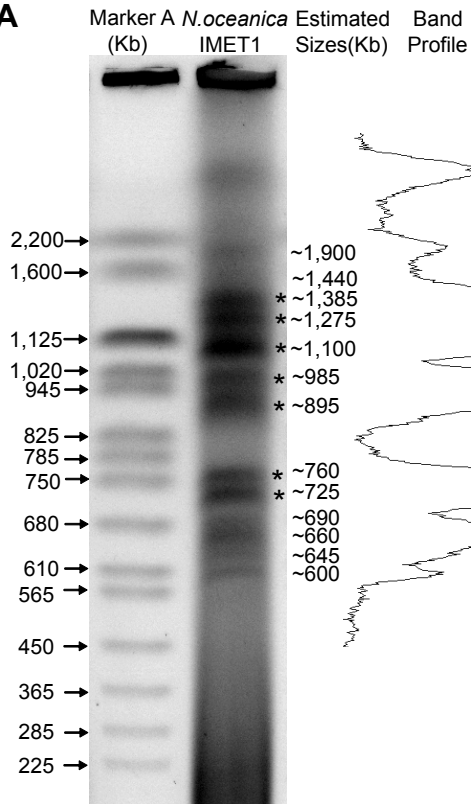**B**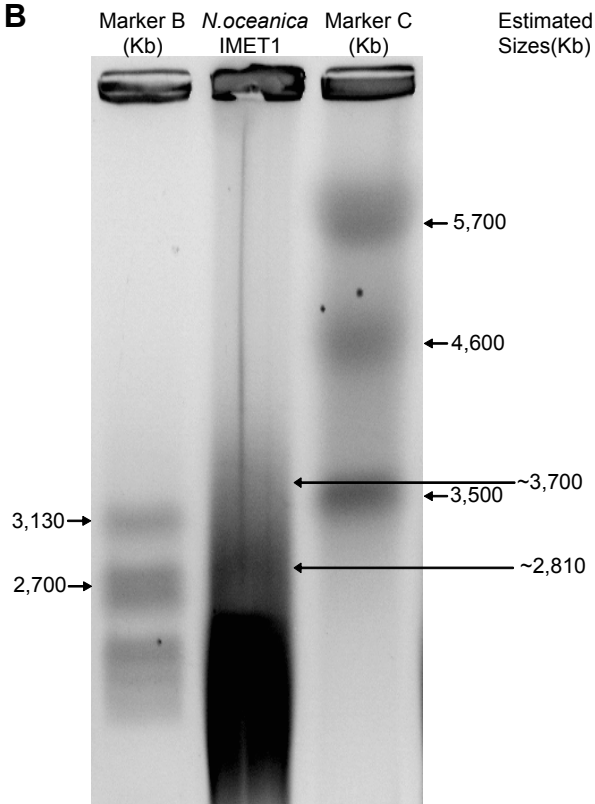

Supplement: Figure S3 — Pulsed-field gel electrophoresis (PFGE) analysis of Nannochloropsis oceanica IMET1 chromosomal DNA. Asterisks (*) indicate those bands that likely represent two or more chromosomes. Three DNA size standards that include Saccharomyces cerevisiae (240–2,200 Kb, Marker A), Hansenula wingei (1–3.1 Mb, Marker B), Schizosaccharomyces pombe (3.5–5.7 Mb, Marker C) were used to estimate chromosome sizes. (A) PFGE profile of IMET1 chromosomes. The band profile as analyzed by ImageJ is shown on the right. (B) PFGE profile of chromosomes larger than 2 Mb. (PDF) [file pgen.1004094.s007.pdf]

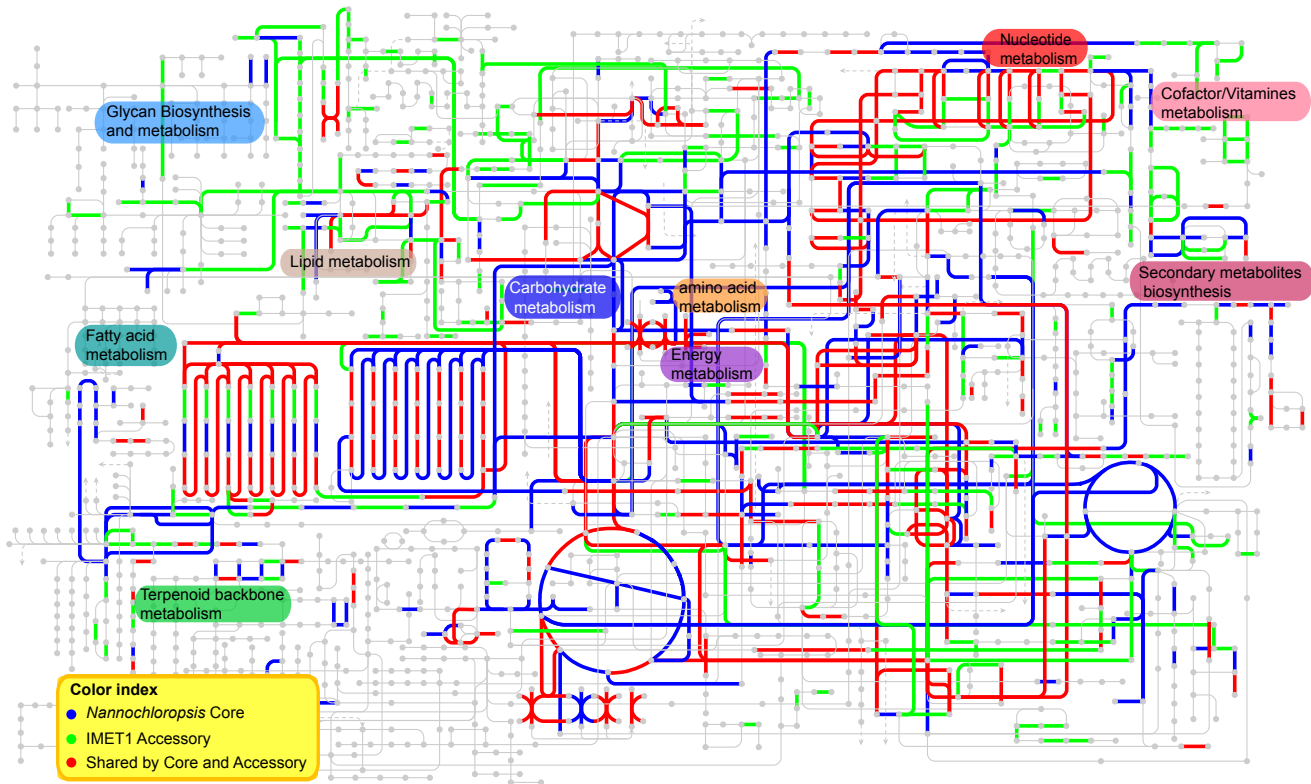

Supplement: Figure S5 — Metabolic conservation and divergence among the six Nannochloropsis strains. Metabolic pathways were represented in IPATH scheme tools. Red edges indicate KEGG IDs identified in both Nannochloropsis core genes and accessory genes. Blue edges indicate those exclusively identified in core genes. Green edges indicate those exclusively identified in accessory genes. (PDF) [file pgen.1004094.s009.pdf]

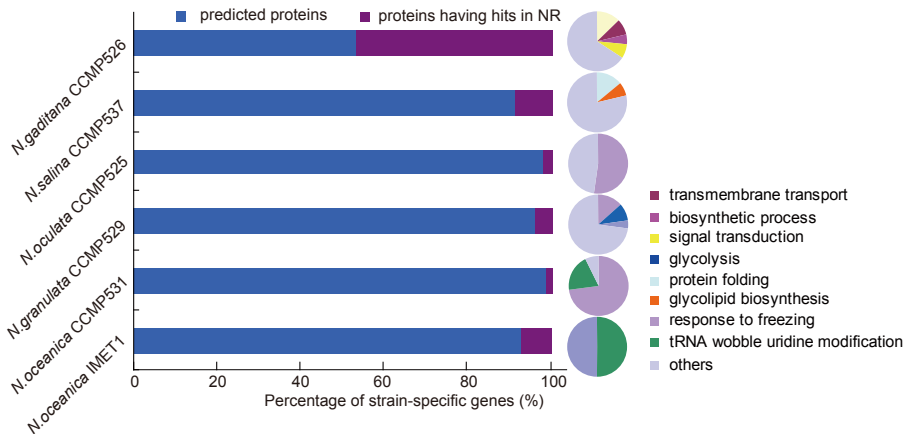

Supplement: Figure S6 — Functional categories of strain-specific genes in N. oceanica IMET1. The GO categories (in biological process) of strain-specific genes in each strain were presented. (PDF) [file pgen.1004094.s010.pdf]

**A**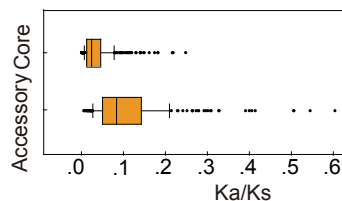**C**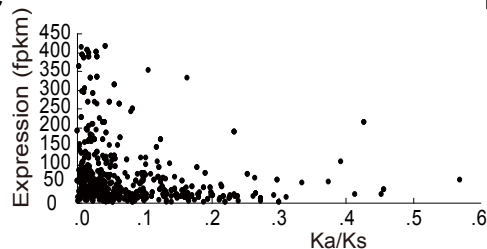**D**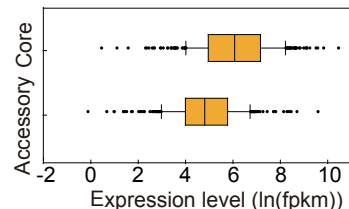**B**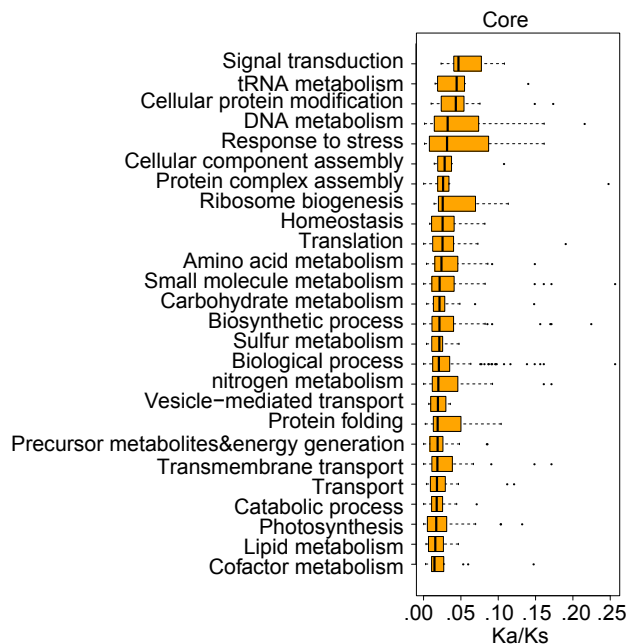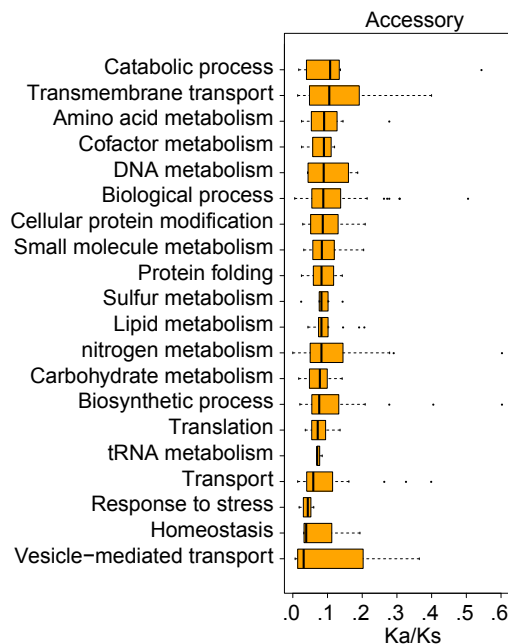

Supplement: Figure S7 — Ka/Ks of core and accessory gene sets and the link to their transcriptional level in N. oceanica. For each of the five-strain orthologous gene sets (excluding the outmost N. gaditana) from the Nannochloropsis core and accessory genes, PAML model M0 was used to estimate a single ω (Ka/Ks, ratio of non-synonymous to synonymous nucleotide divergence) as a measure of the selective pressure that the gene sets were under. (A) Different Ka/Ks of core and accessory proteins. (B) Selective pressure on the functional categories associated with Nannochloropsis core and accessory genes. GO slim terms in biological process with at least three genes associated are shown. Yellow box-plot indicates smallest observation (sample minimum), lower quartile (Q1), upper quartile (Q3), largest observation (sample maximum) and outlier(s) of ω values in a functional category. (C) Negative correlation between the Ka/Ks and the transcriptional level of a gene. (D) Different transcriptional levels of core and accessory genes. (PDF) [file pgen.1004094.s011.pdf]

**A**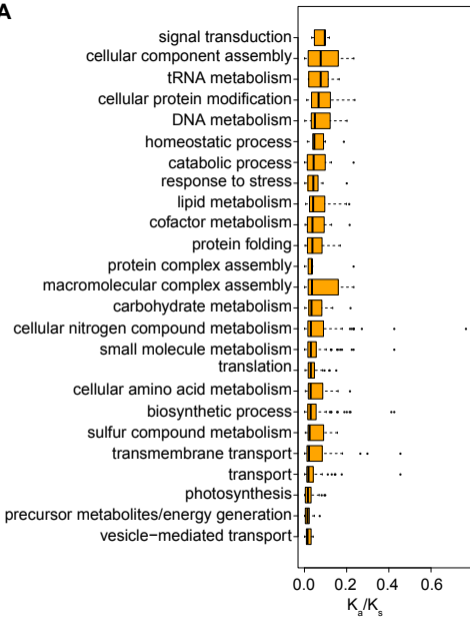**B**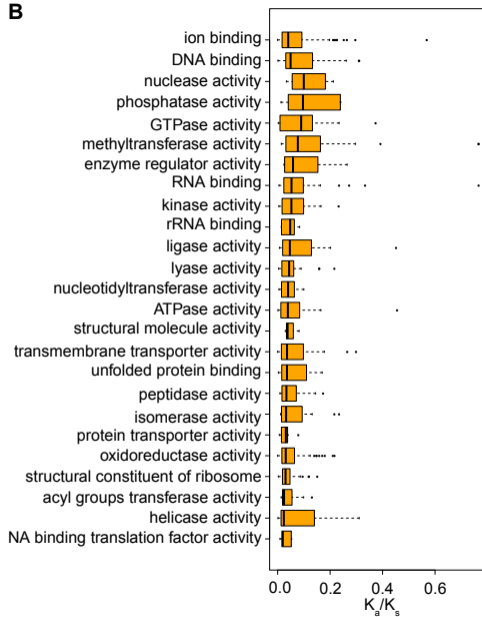**C**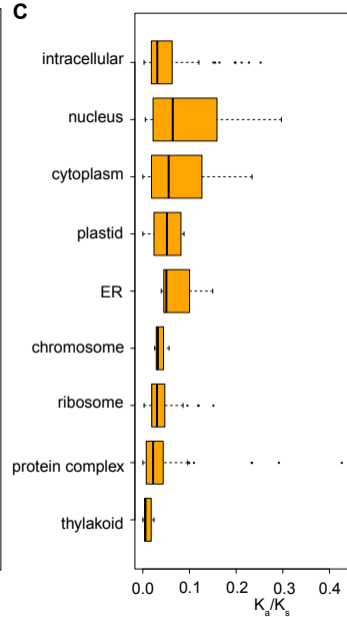

Supplement: Figure S8 — Selective pressure on protein-coding genes in Nannochloropsis spp. For each of the 1,085 six-way single-copy orthologous gene sets, PAML model M0 was used to estimate a single ω (Ka/Ks, ratio of non-synonymous to synonymous nucleotide divergence) that is fixed across the reconstructed whole-genome phylogeny. The associated GO slim terms that have at least five genes are shown for (A) Biological Process, (B) Molecular Function and (C) Cellular Component. The red dots represent the median ω in a functional category. The yellow box-plot shows the smallest observation (sample minimum), lower quartile (Q1), upper quartile (Q3), largest observation (sample maximum) and outlier(s) of ω values in a functional category. (PDF) [file pgen.1004094.s012.pdf]

**A**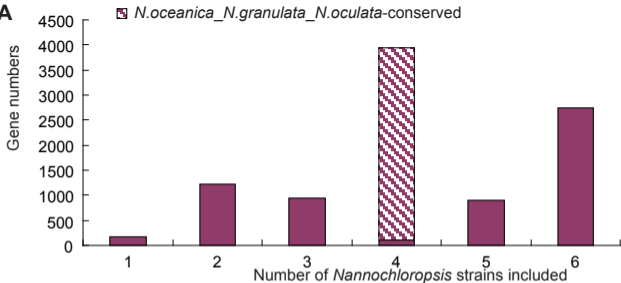**B**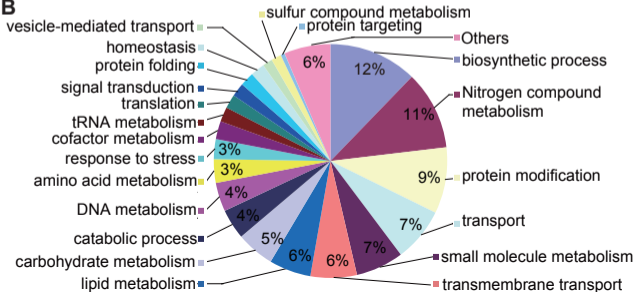

Supplement: Figure S9 — Conservation of IMET1 genes in the other five Nannochloropsis strains. (A) A histogram of the number of IMET1 genes conserved in a series of strain sets. The diagonal in the two-strain sets represents genes that were exclusively conserved in N. oceanica species; the diagonal in the four-strain sets represents genes that were exclusively conserved in N. oceanica (IMET1 and CCMP531), N. granulata and N. oculata. (B) Functional categories in the GO slim hierarchy of genes that were exclusively conserved in N. oceanica, N. granulata and N. oculata. Categories with percentages less than 1% are pooled and presented as “Others.” (PDF) [file pgen.1004094.s013.pdf]

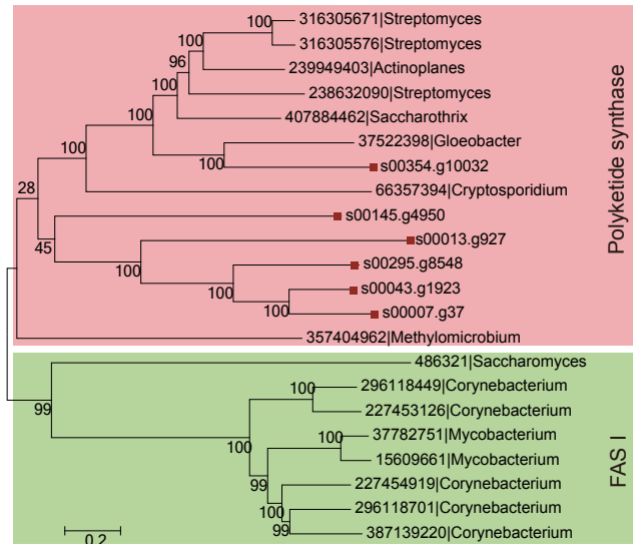

Supplement: Figure S10 — Phylogeny of type I fatty acid synthase. Multiple sequence alignments among six putative PKS proteins from IMET1 (indicated by a red square), as well as type I PKS proteins and type I fatty acid synthases (FAS) from bacteria, as indicated by the species name, were generated with ClustalW. The phylogenetic tree was constructed using the neighbor-joining method in MEGA5 with a bootstrap test (based on 100 replicates). (PDF) [file pgen.1004094.s014.pdf]

**A**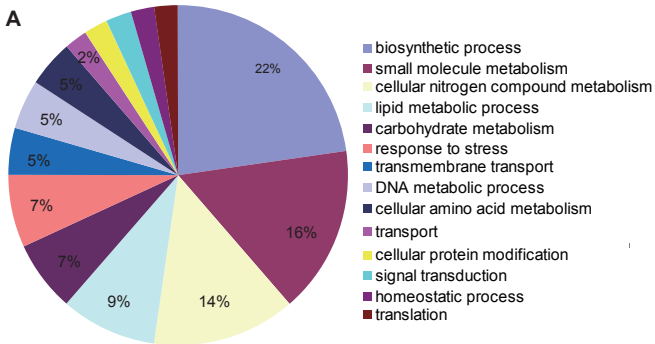**B**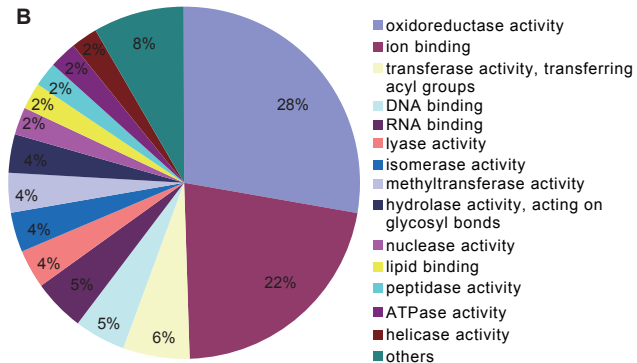

Supplement: Figure S11 — Functional categories of horizontally transferred genes in N. oceanica IMET1. (A) The GO Slim categories (in biological process) of the HGT genes in IMET1. (B) The GO Slim categories (in molecular function) of the HGT genes in IMET1. (PDF) [file pgen.1004094.s015.pdf]

A

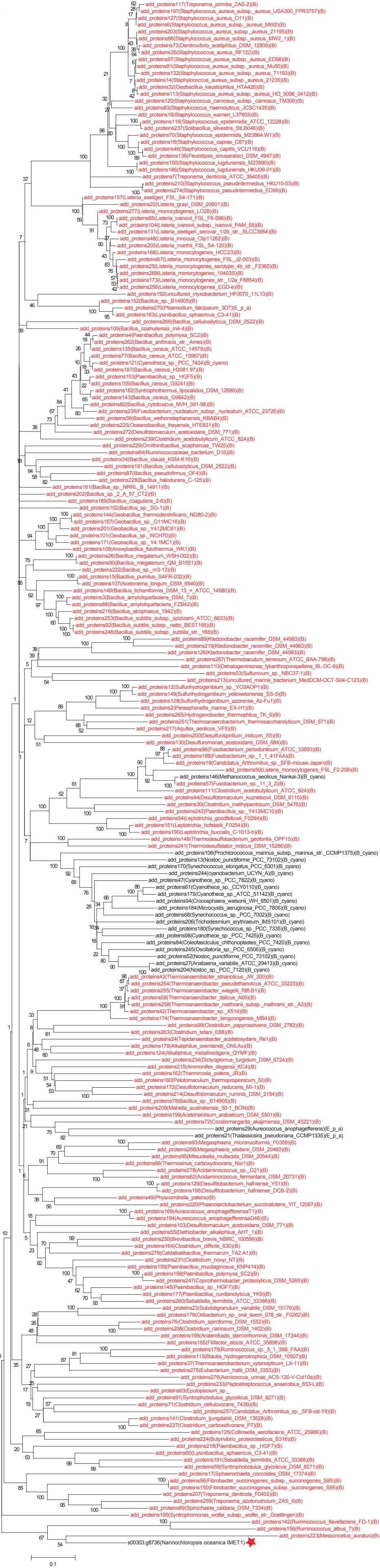

B

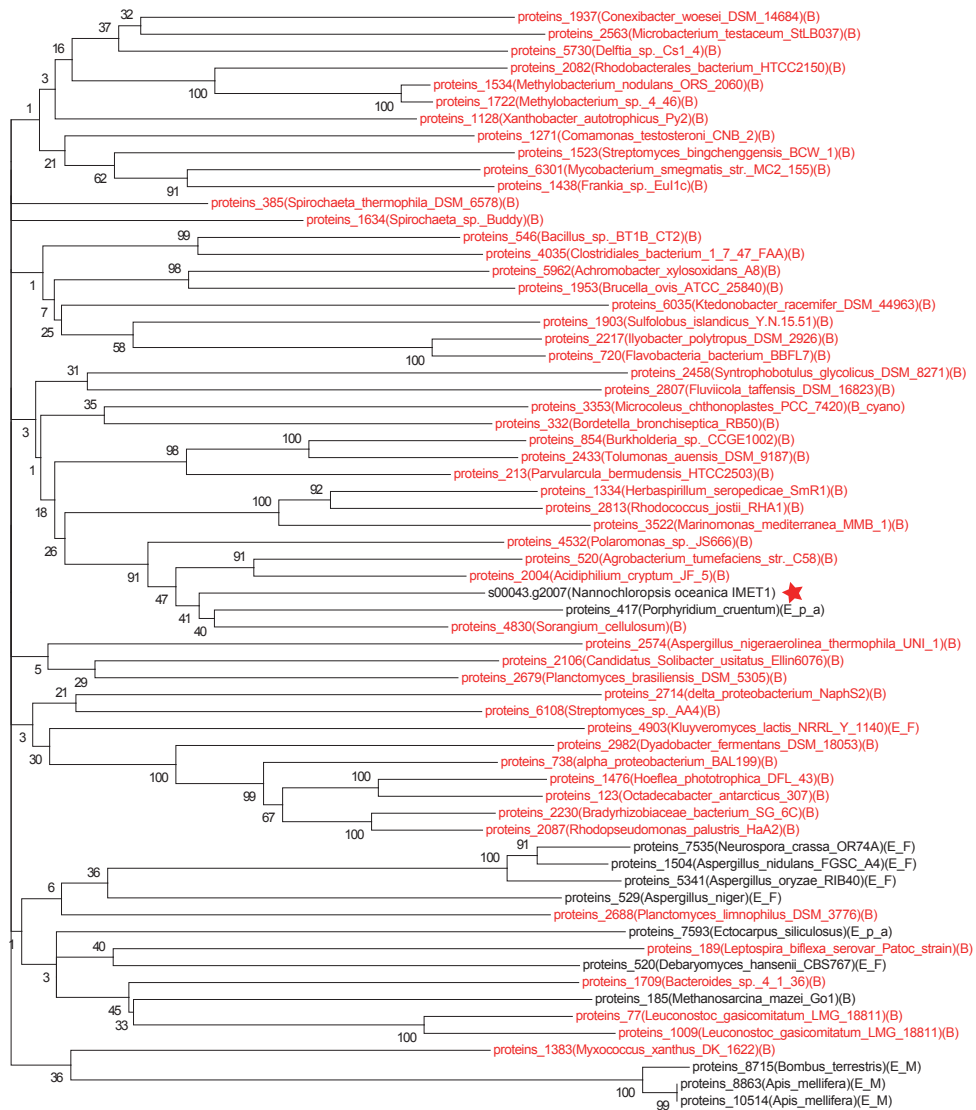

0.1

C

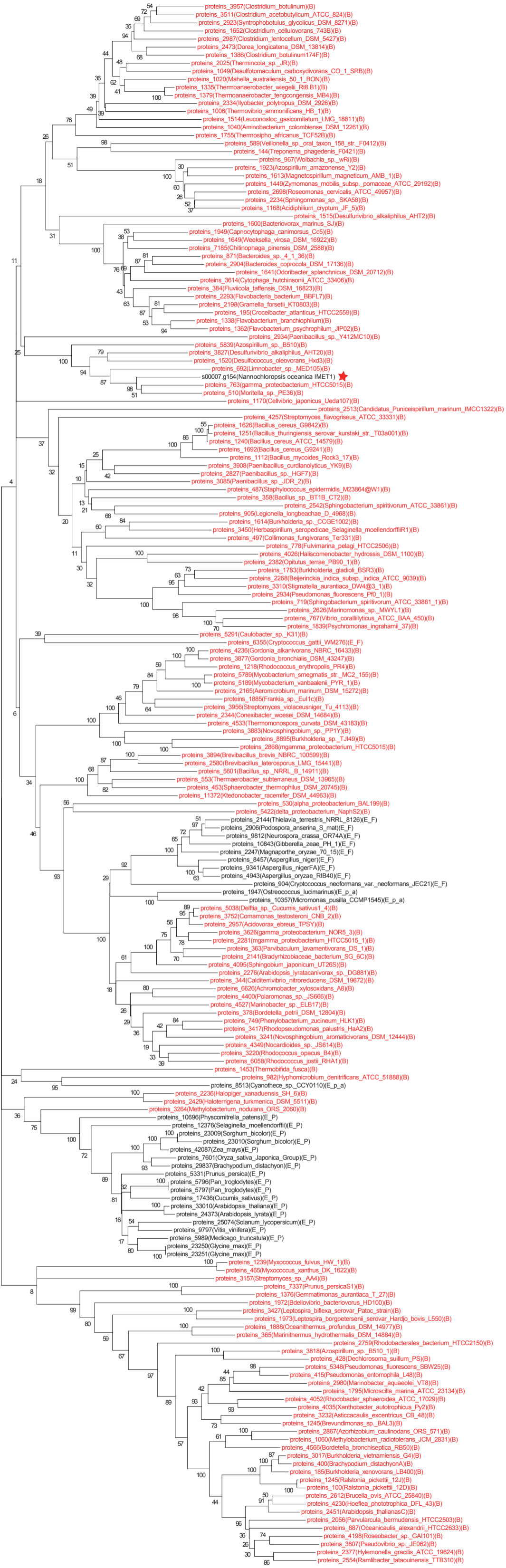

0.1

C

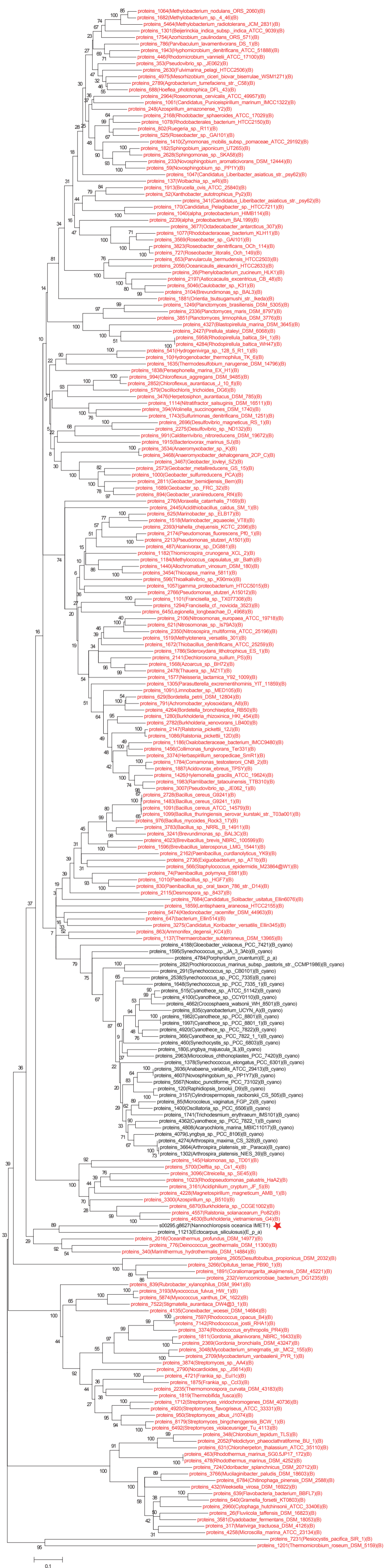

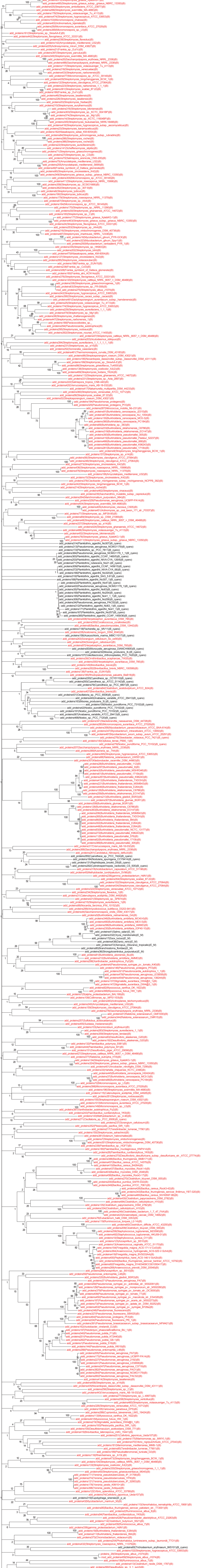

E

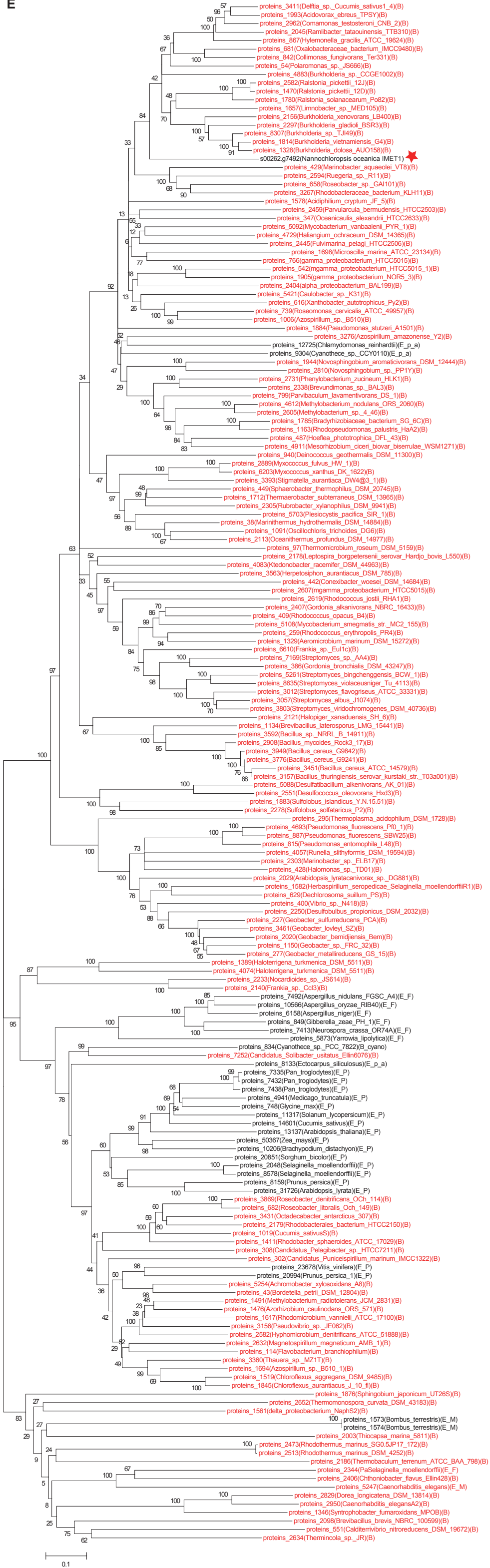

E

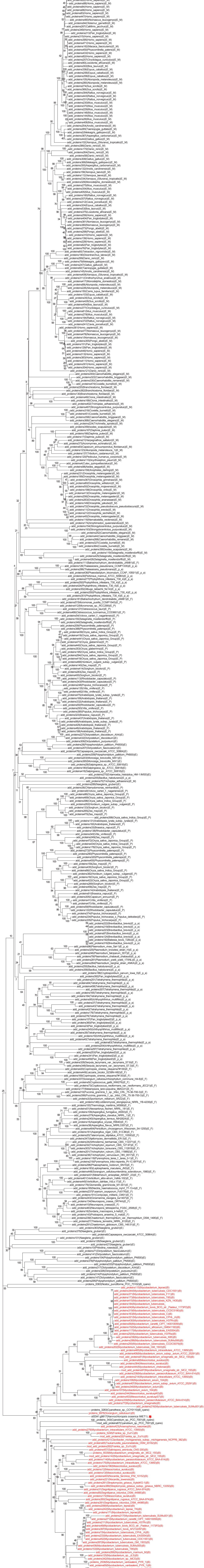

F

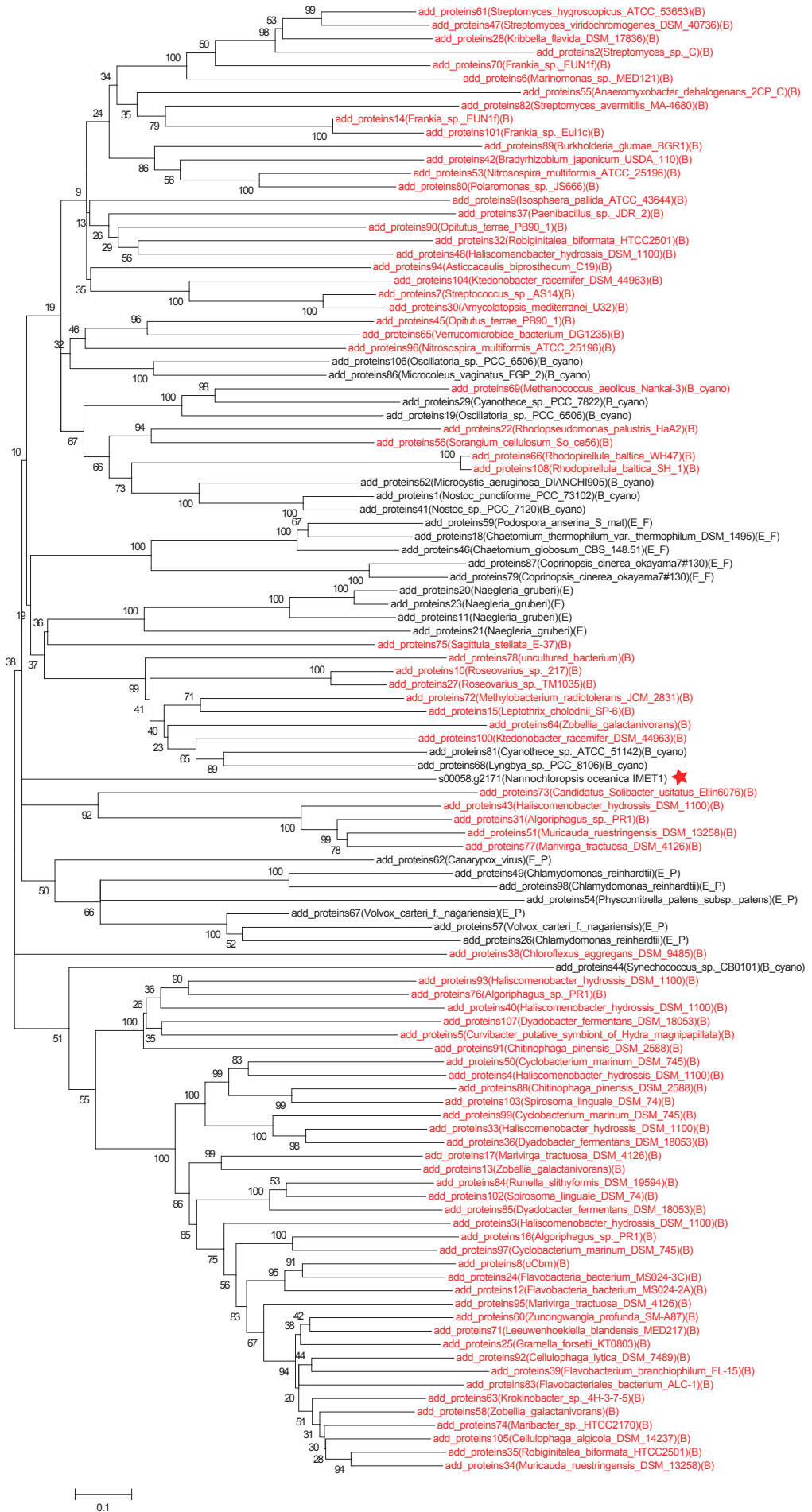

**F**

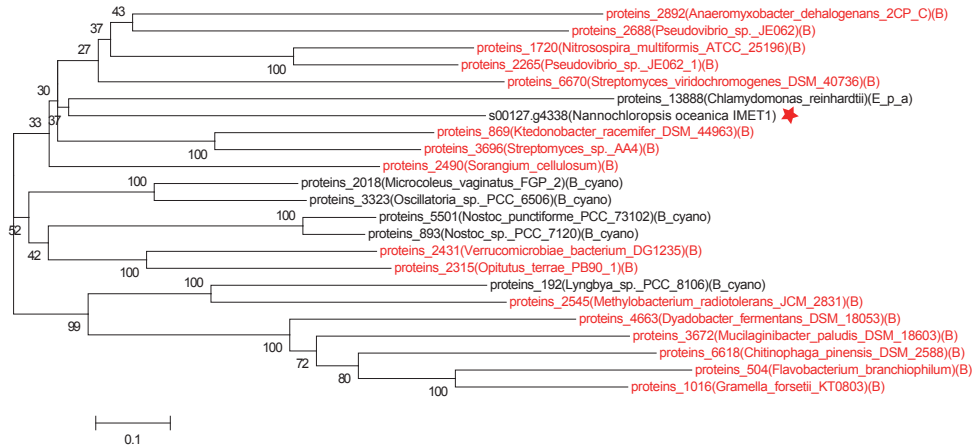

Supplement: Figure S12 — Phylogenies by the NJ method for lipid synthesis genes that were inferred to have originated from HGT. MUSCLE was used to perform multiple alignments for each of the lipid synthesis genes (indicated by a red star) and their orthologous genes generated by Inparanoid Program. Neighbor-joining trees were constructed in MEGA5 with the bootstrapping method (based on 100 replicates), using the passion correction model to calculate evolutionary distances. Branches of bacterial sequences (with the exception of cyanobacteria) are highlighted in red. (A) Phylogeny of KAS gene (ID: s00303.g8736) gene; (B) phylogeny of KAR gene (s00043.g2007); (C) phylogeny of ENR genes (s00007.g154, s00295.g8627); (D) phylogeny of the acyl-ACP TE gene (s00355.g10346); (E) phylogeny of LCFACS genes (s00262.g7492, scaffold00341.g9817); (F) phylogeny of PAP genes (s00058.g2171, s00127.g4338). (PDF) [file pgen.1004094.s016.pdf]

A

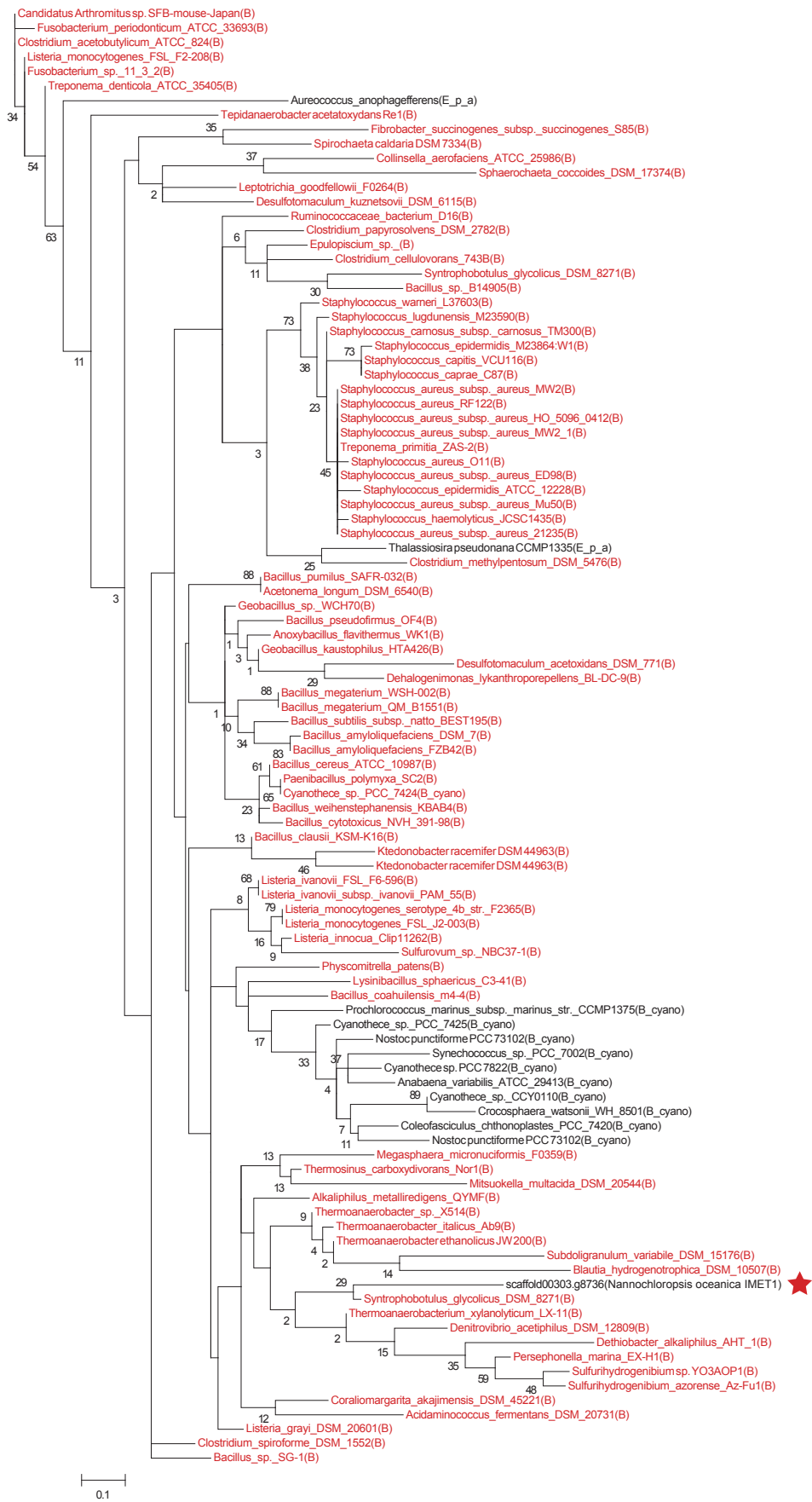

B

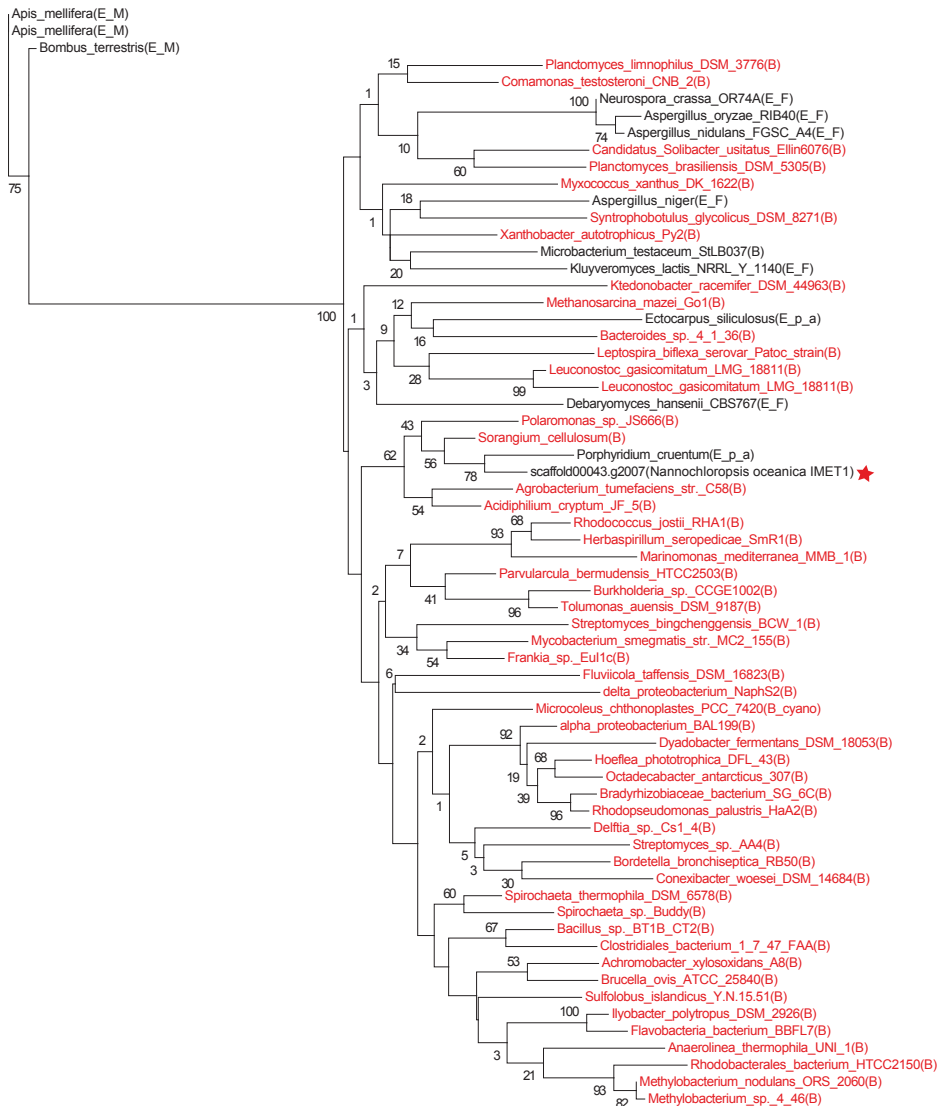

0.2

C

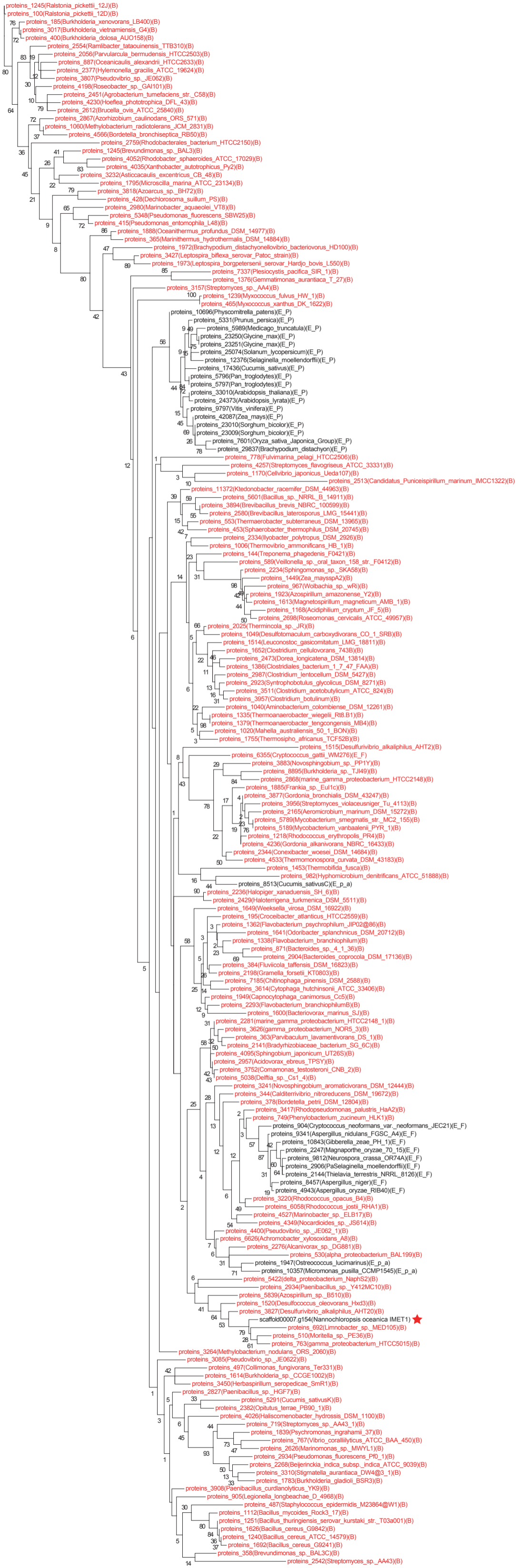

0.2

C

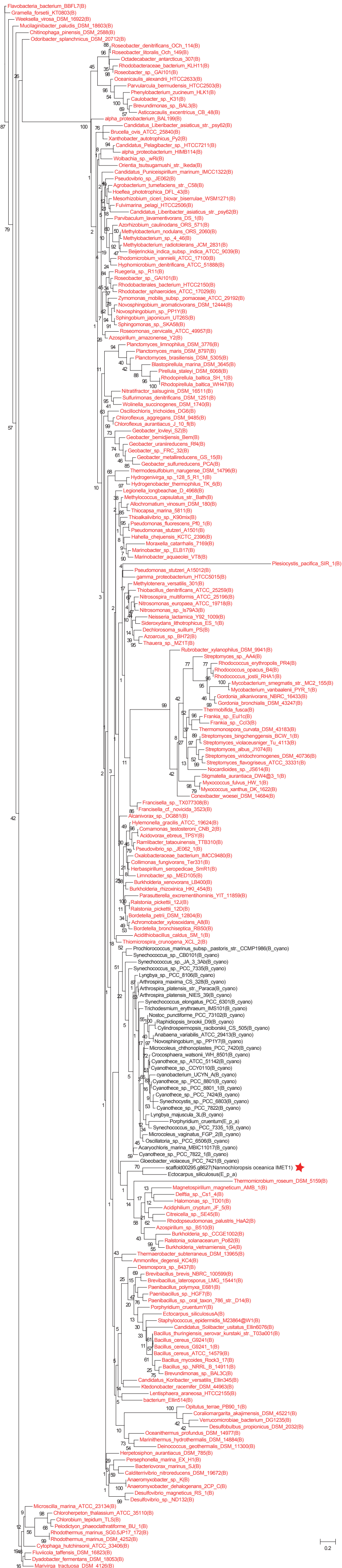

0.2

D

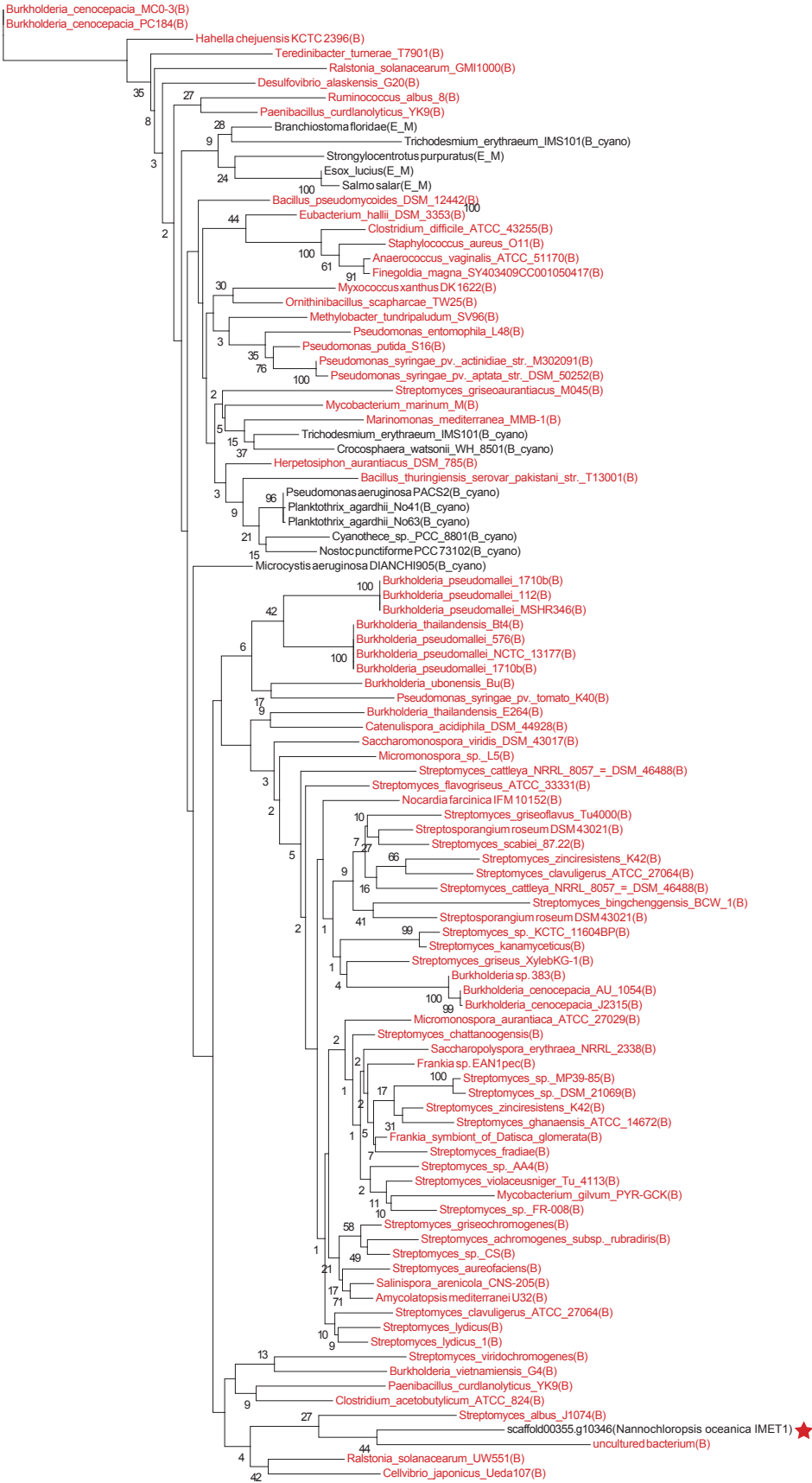

0.2

E

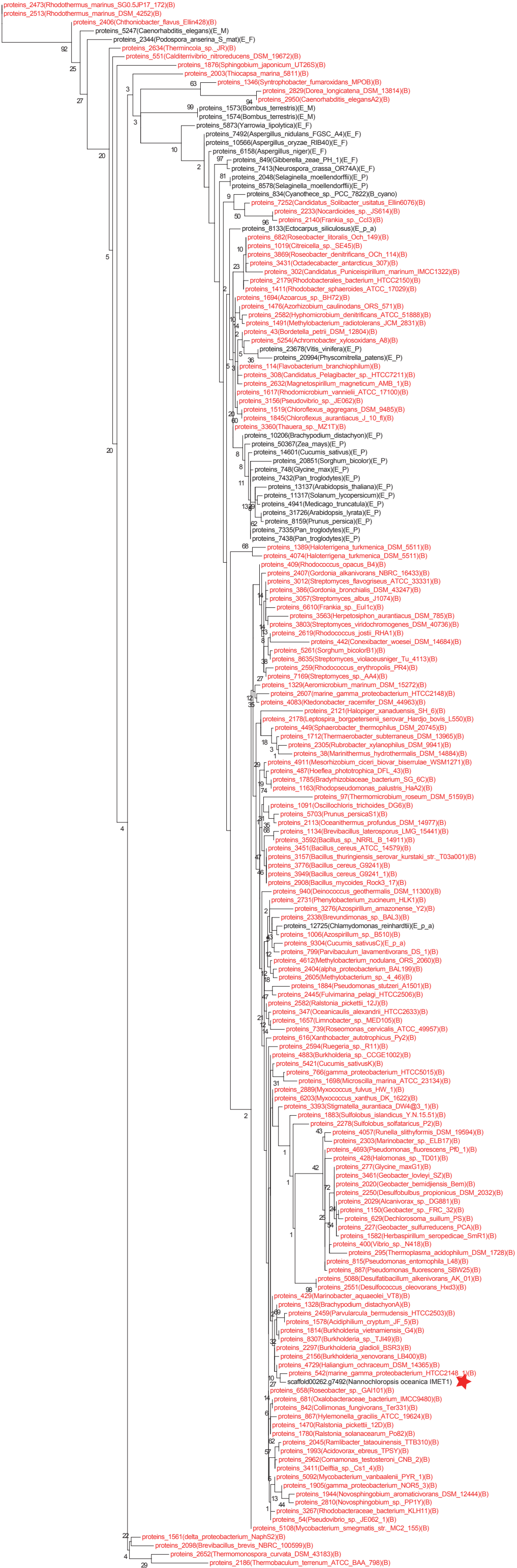

0.5

E

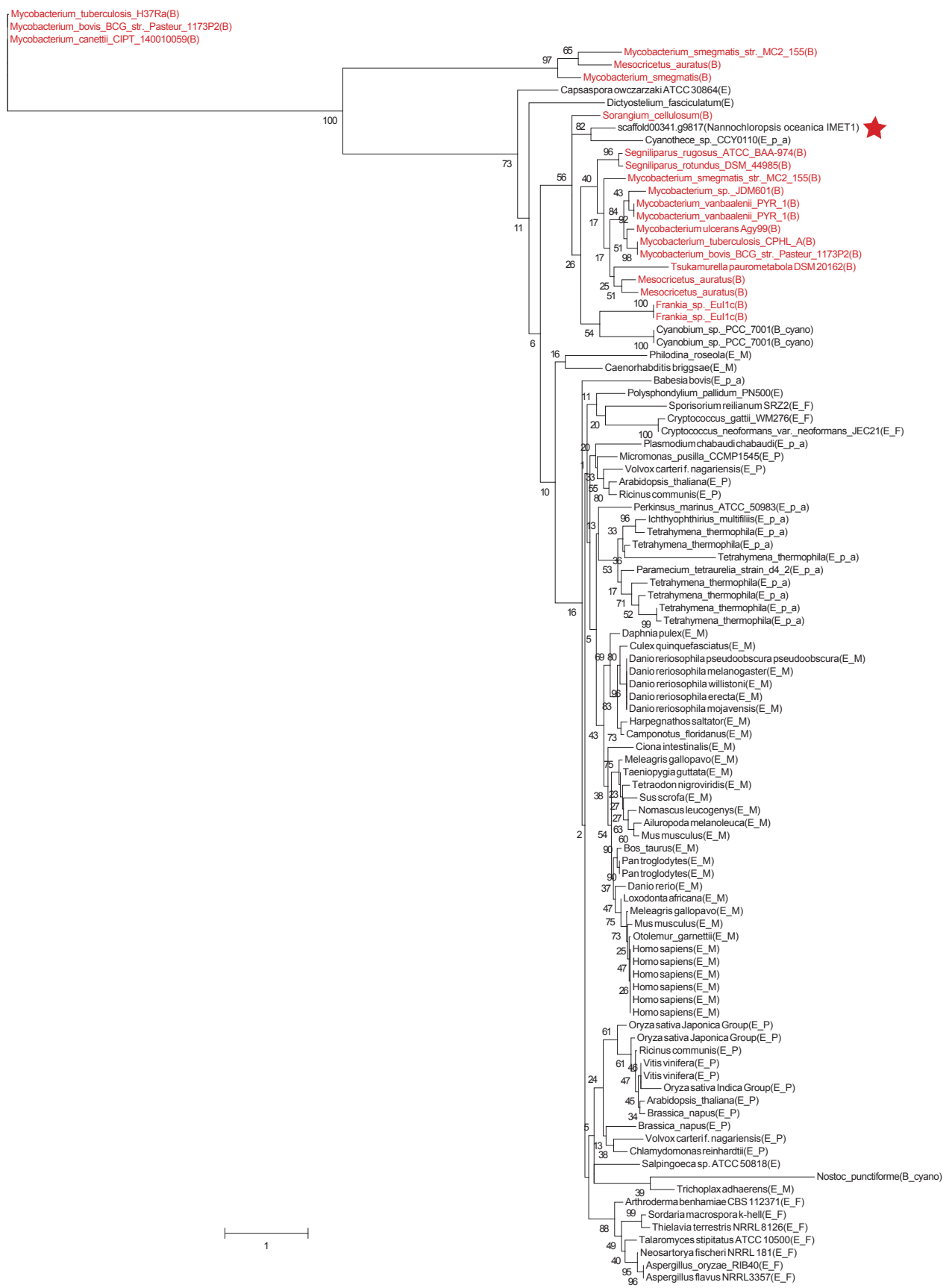

F

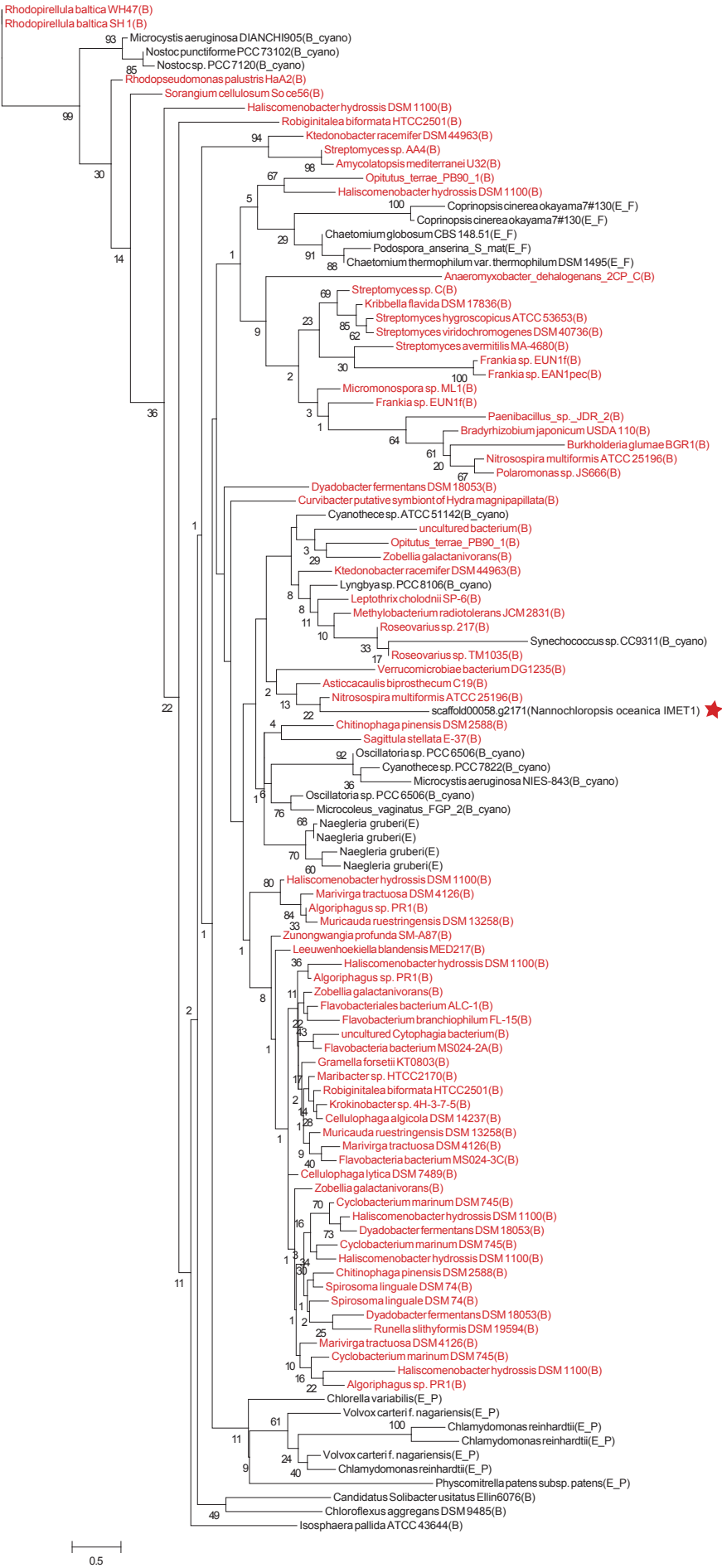

**F**

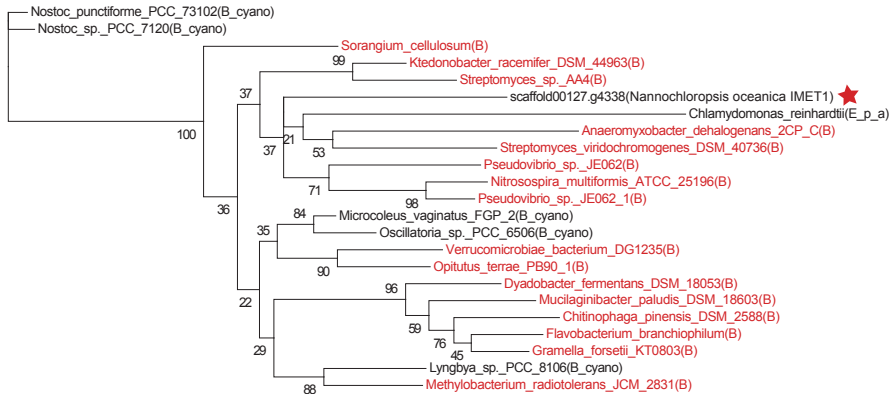

0.2

Supplement: Figure S13 — Phylogenies by the ML method for lipid synthesis genes that were inferred to have originated from HGT. MUSCLE was used to perform multiple alignments for each of the lipid synthesis genes (indicated by a red star) and their orthologous genes generated by Inparanoid Program. ML trees were constructed in PhyML program by the best protein evolution model selected by ProtTest, using 100 bootstrapping replicates. Branches of bacterial sequences (with the exception of cyanobacteria) are highlighted in red. (A) Phylogeny of KAS gene (ID: s00303.g8736) gene; (B) phylogeny of KAR gene (s00043.g2007); (C) phylogeny of ENR genes (s00007.g154, s00295.g8627); (D) phylogeny of the acyl-ACP TE gene (s00355.g10346); (E) phylogeny of LCFACS genes (s00262.g7492, scaffold00341.g9817); (F) phylogeny of PAP genes (s00058.g2171, s00127.g4338). (PDF) [file pgen.1004094.s017.pdf]

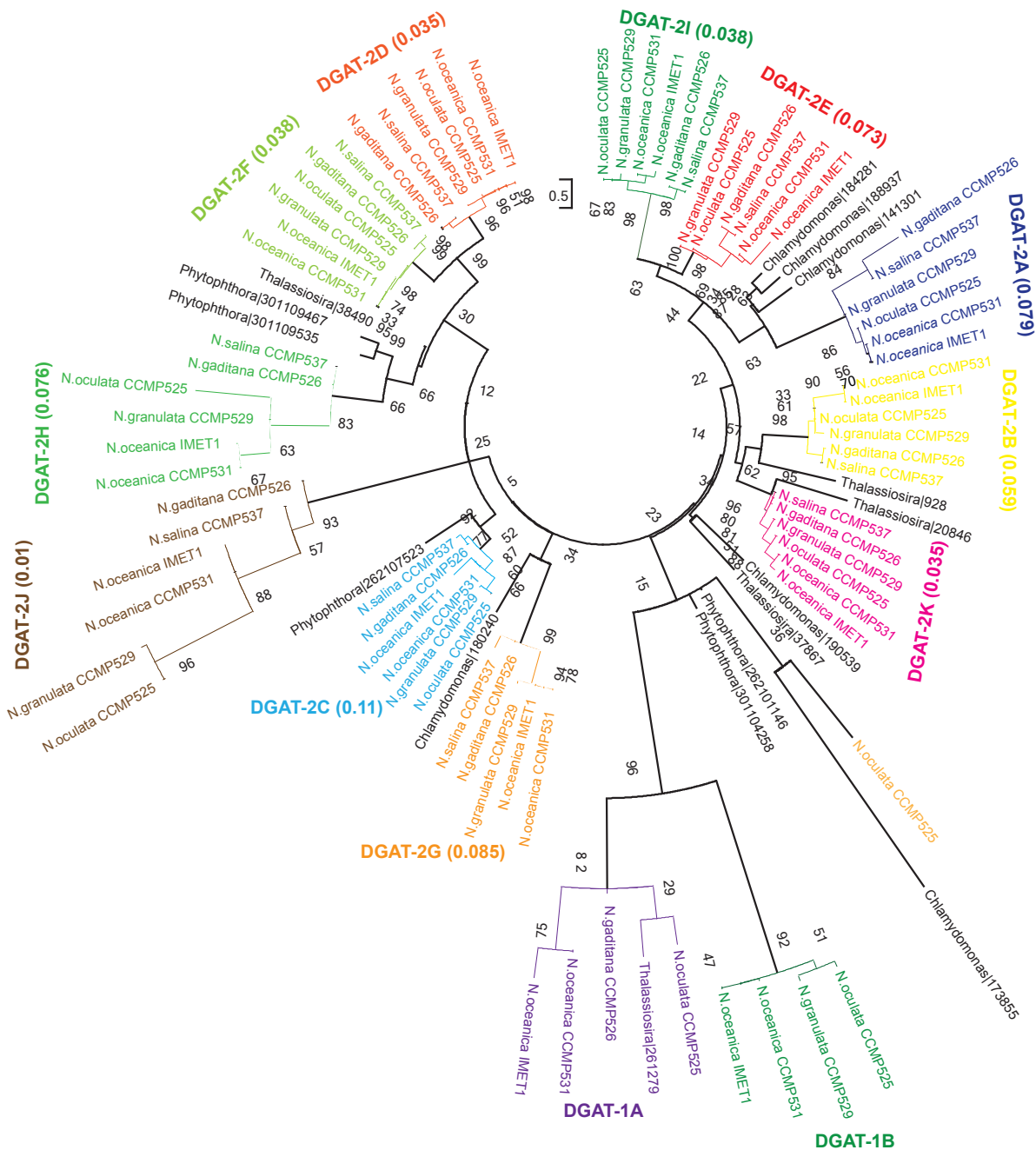

Supplement: Figure S14 — Phylogeny of DGAT genes in the six Nannochloropsis genomes. DGAT genes from all the six Nannochloropsis strains, as well as lineages representing bacteria, fungi, microalgae and plants, were aligned in ClustalW. A phylogenetic tree was constructed using the neighbor-joining method in MEGA5 with a bootstrap test (based on 100 replicates). Orthologs (from the six Nannochloropsis strains) of each DGAT in IMET1 are indicated with the same color. The Ka/Ks value of each orthologous group of DGAT-2 in Nannochloropsis is shown next to the Group ID. (PDF) [file pgen.1004094.s018.pdf]

A

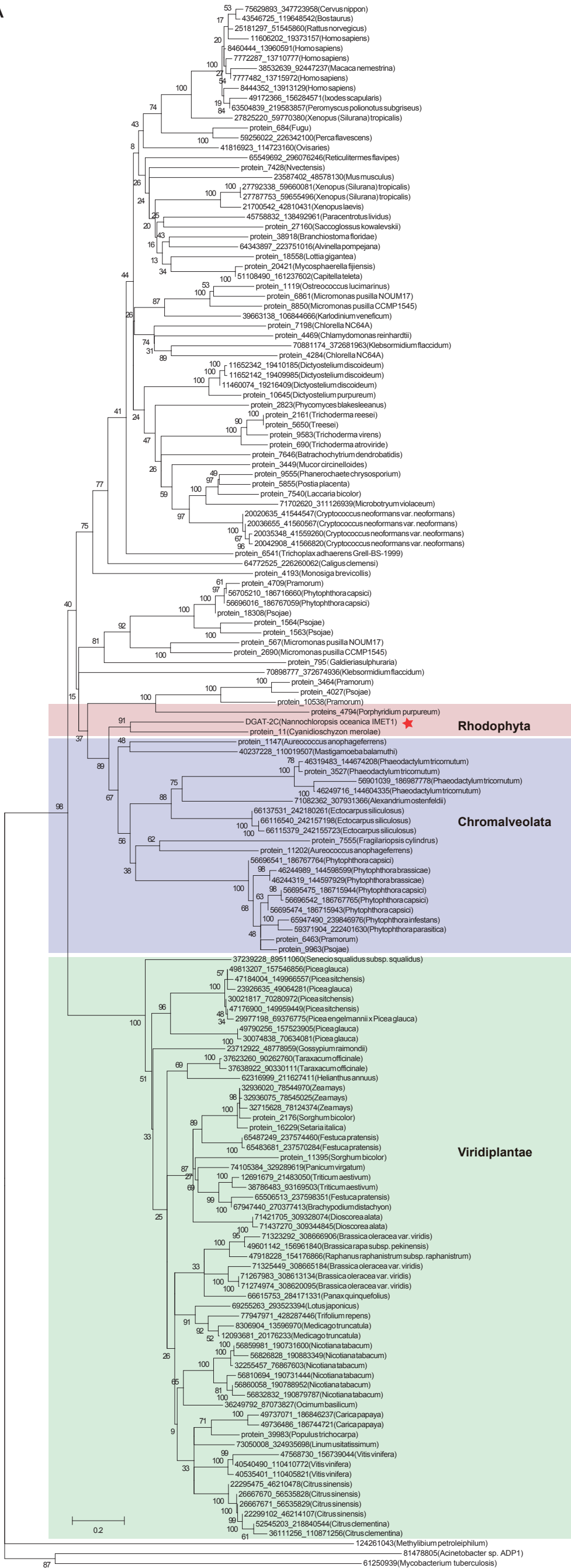

B

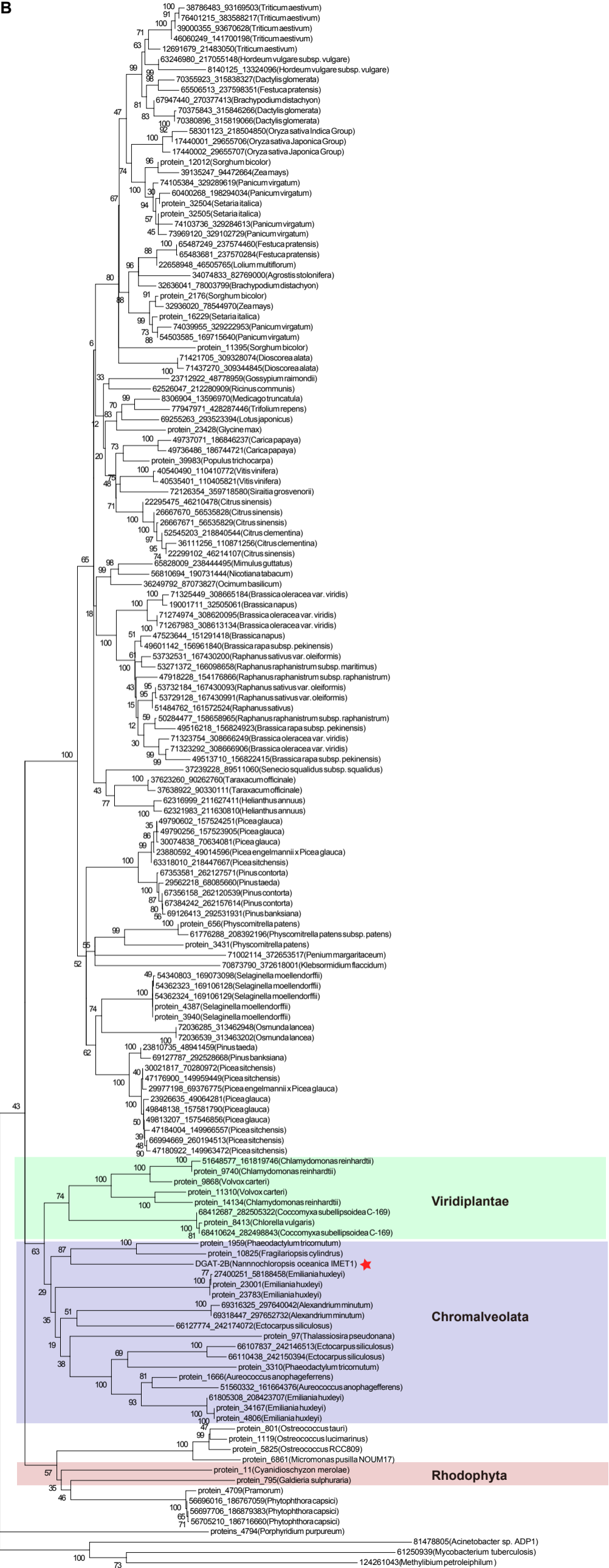

C

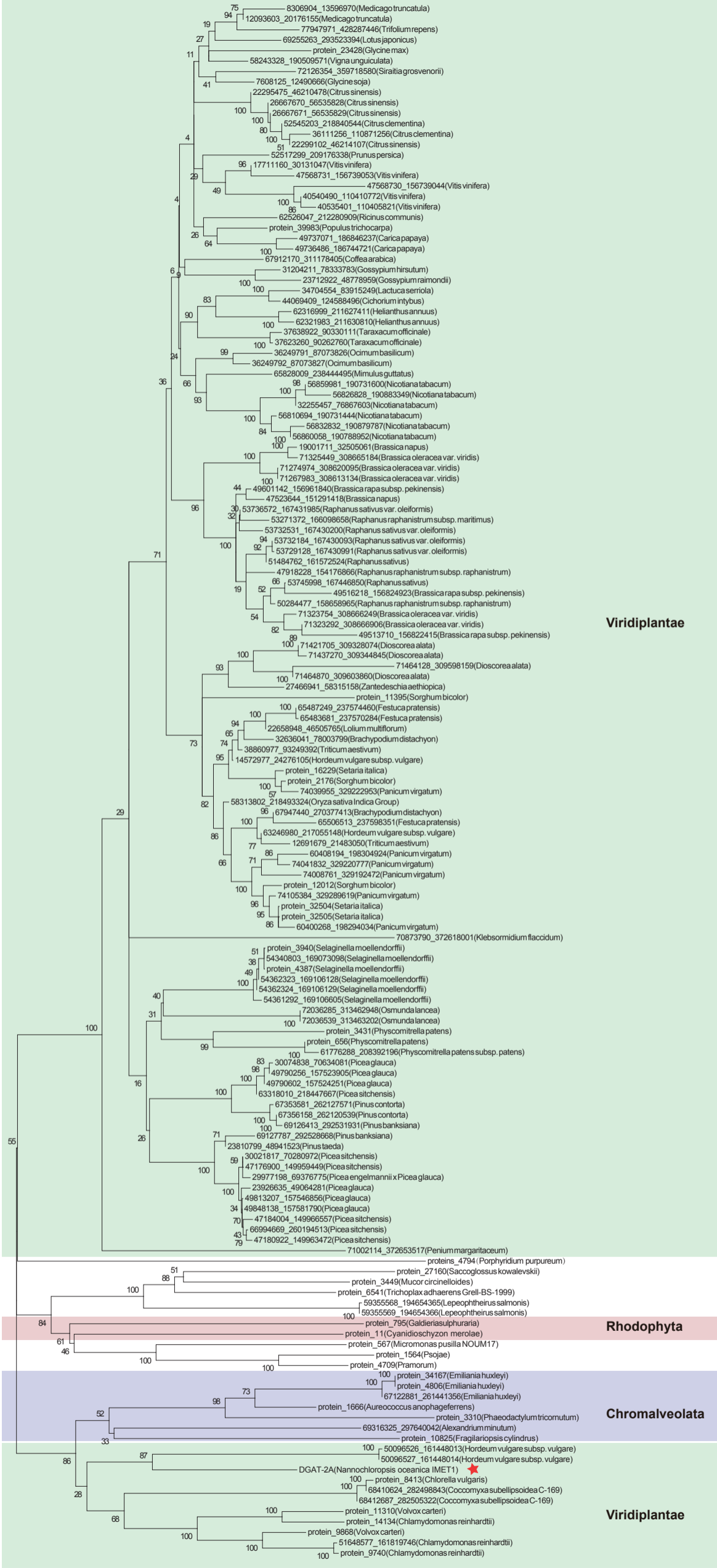

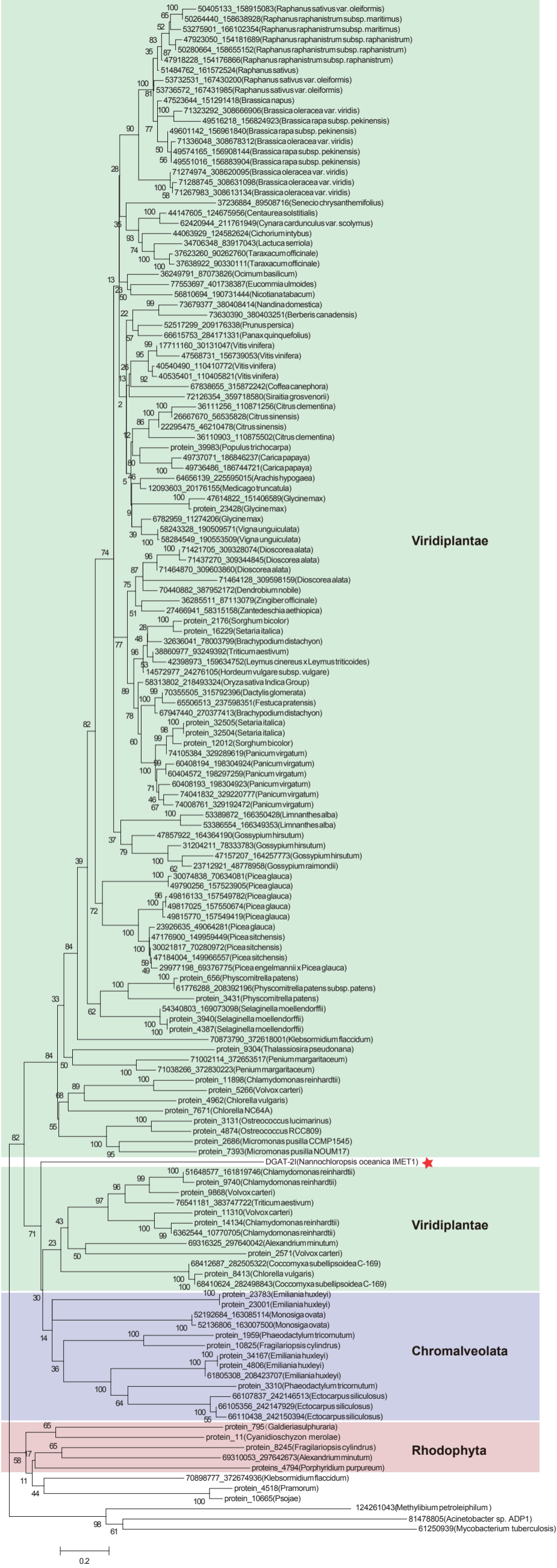

E

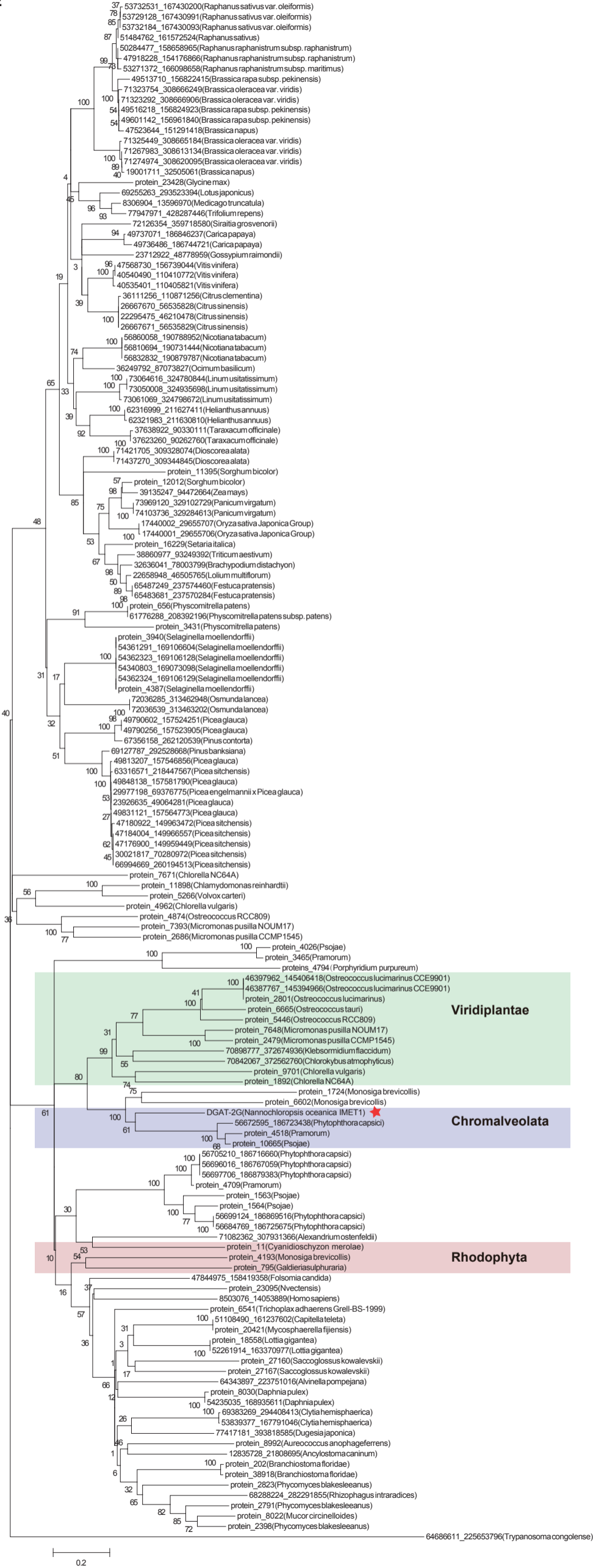

F

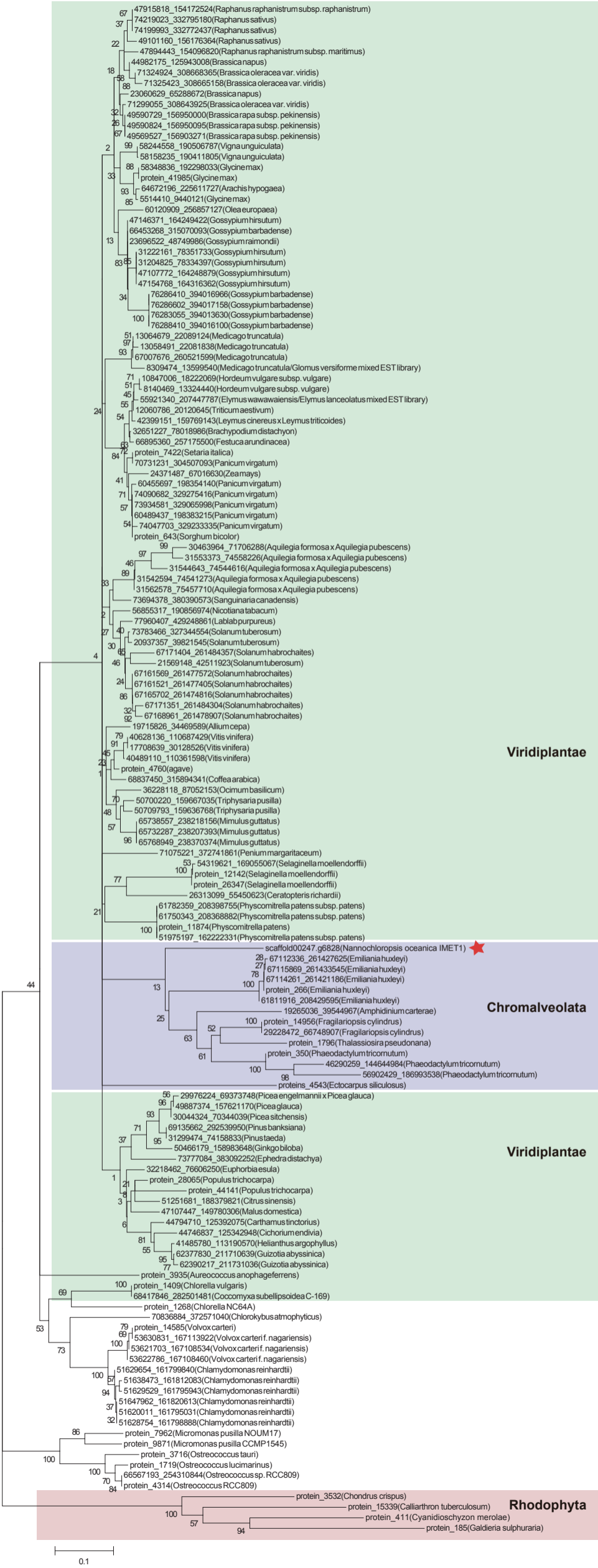

Supplement: Figure S15 — Phylogenies by the NJ method for lipid synthesis genes that were inferred to have originated from secondary endosymbionts. DGAT-2 from N. oceanica IMET1 are indicated by red stars. (A) Phylogeny of DGAT-2C; (B) phylogeny of DGAT-2B; (C) phylogeny of DGAT-2A; (D) phylogeny of DGAT-2I; (E) phylogeny of DGAT-2G; (F) phylogeny of MCAT. (PDF) [file pgen.1004094.s019.pdf]

A

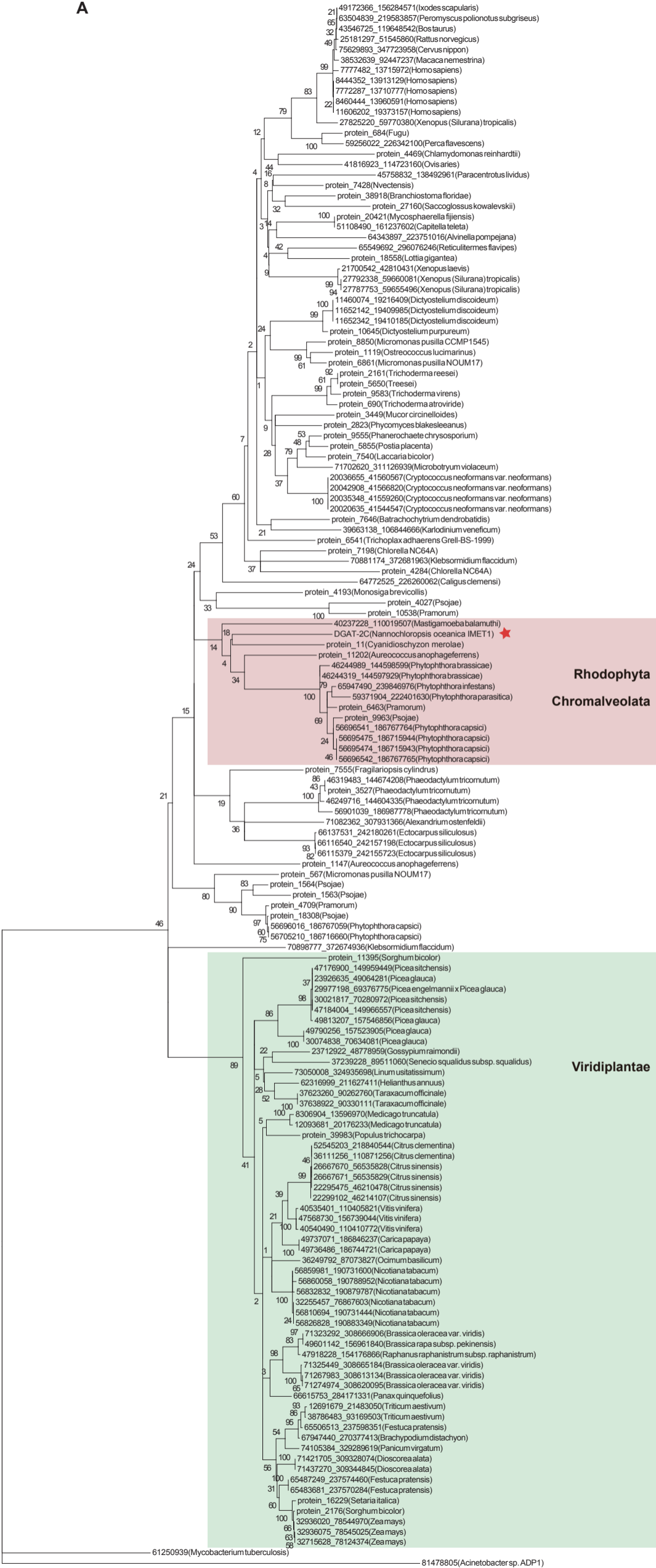

0.5

B

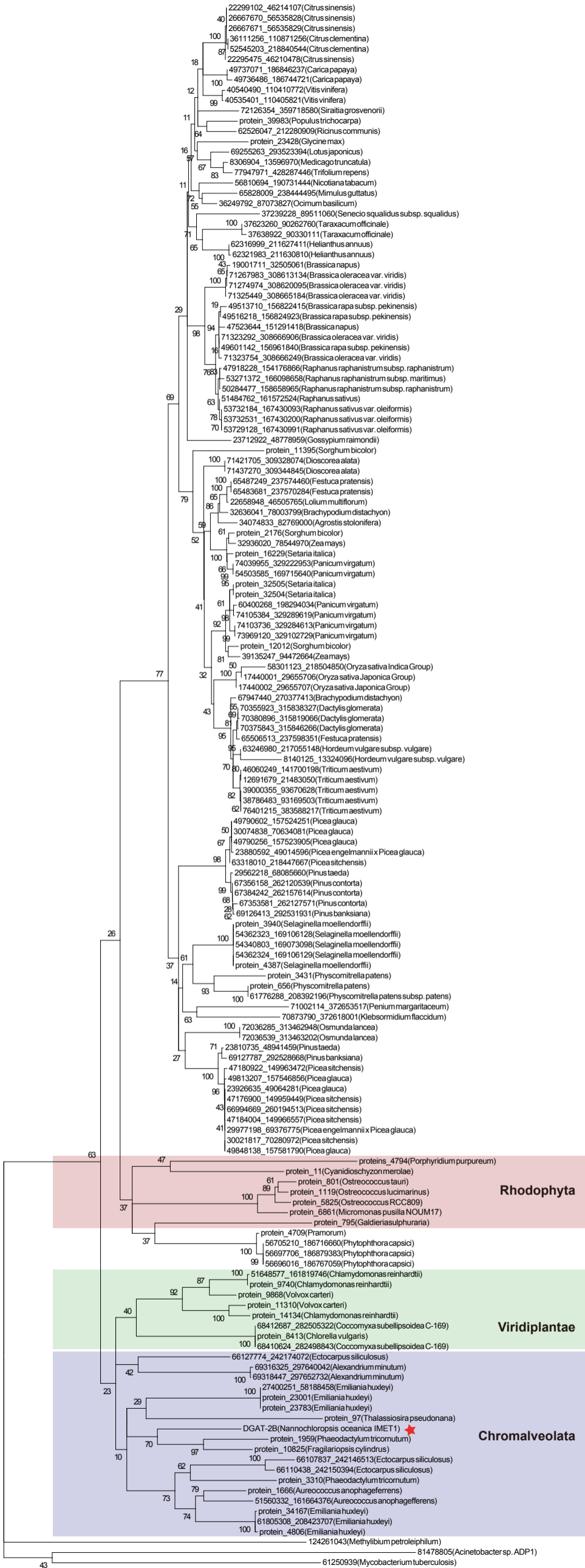

C

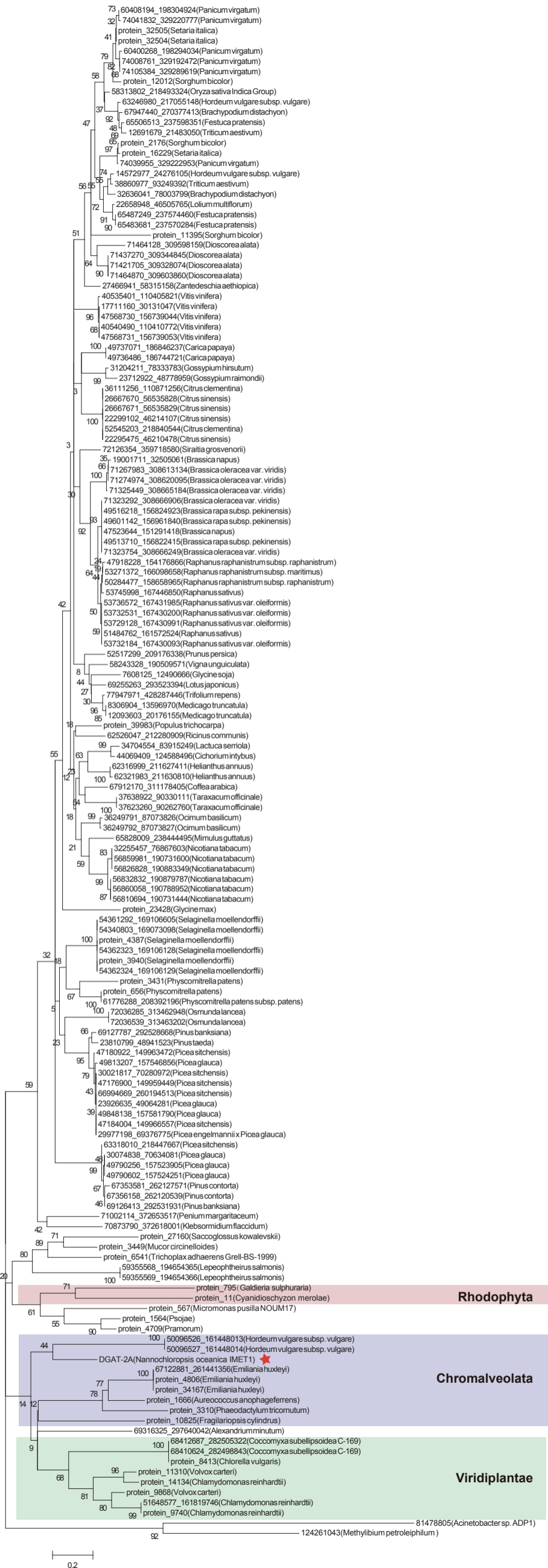

D

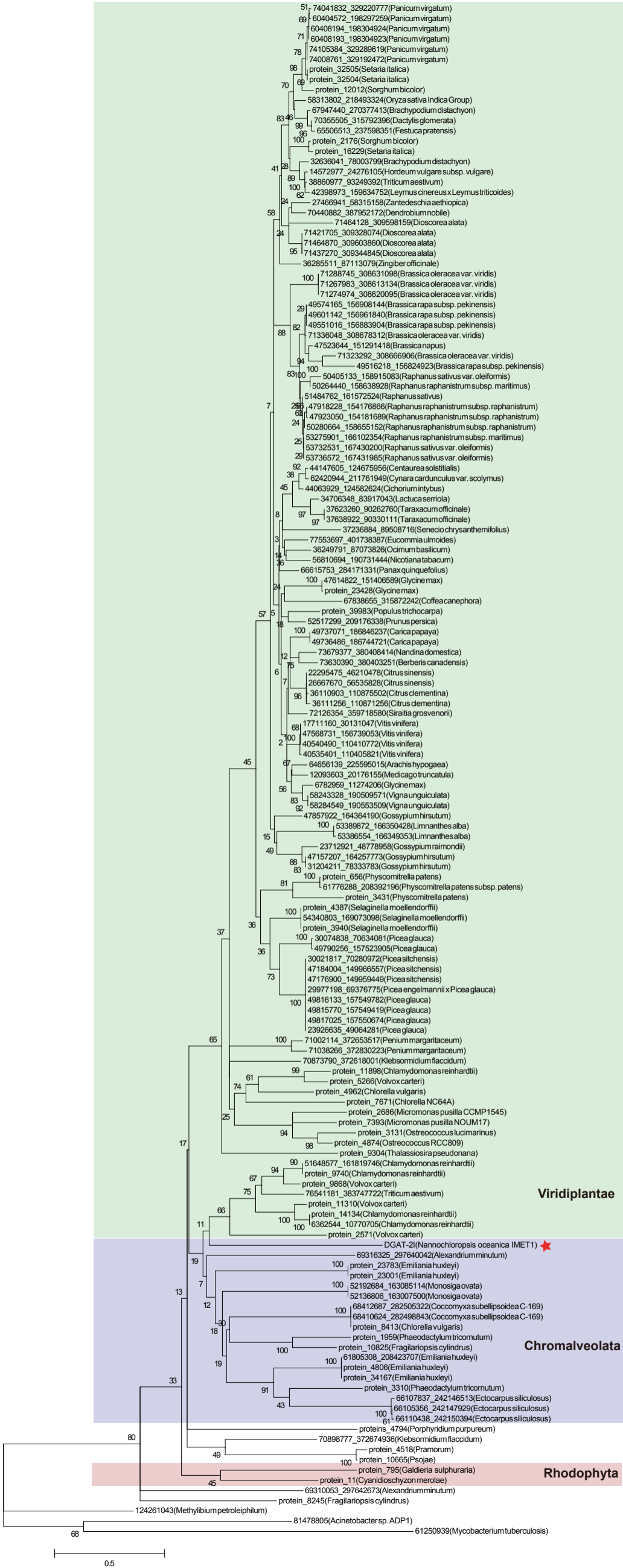

E

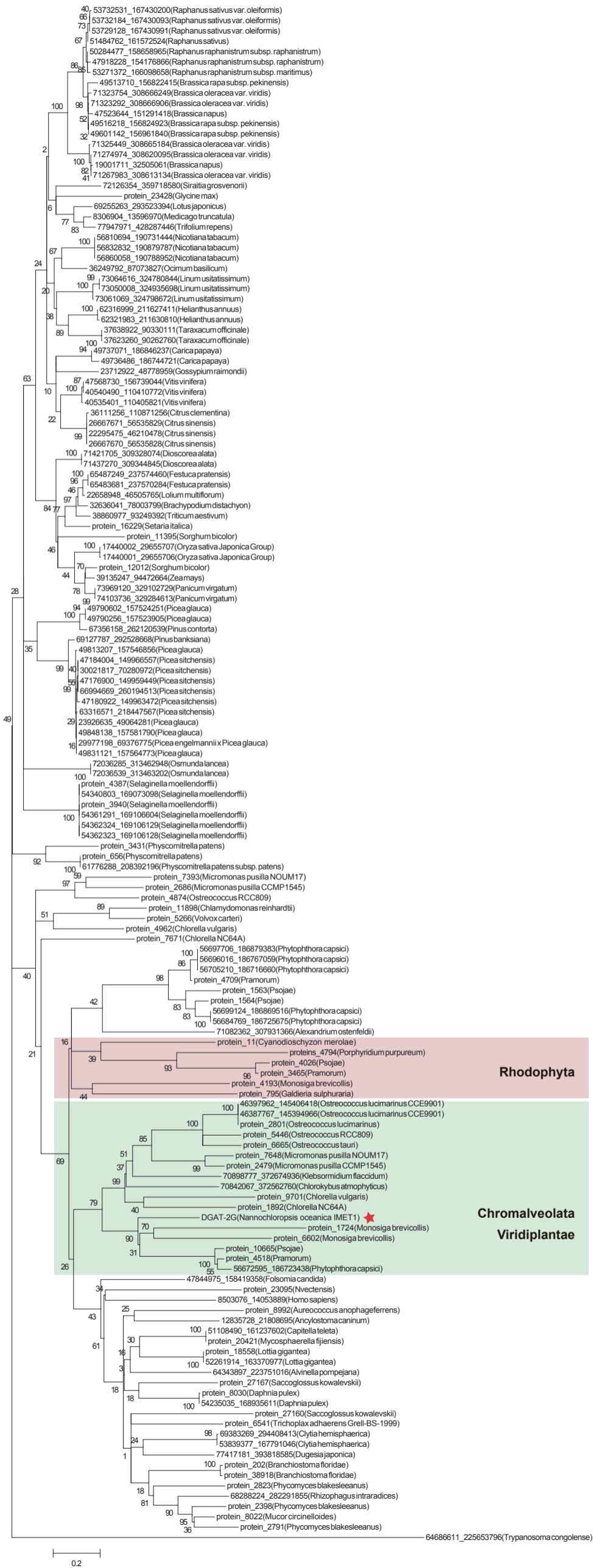

F

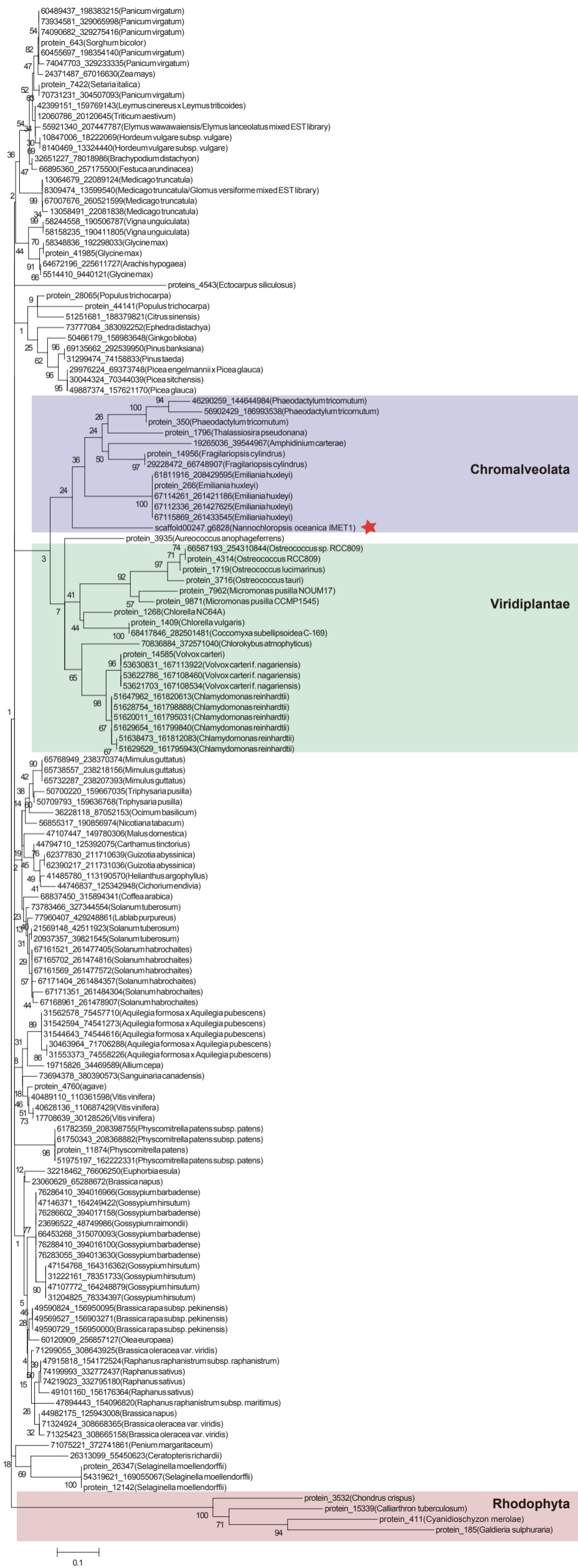

Supplement: Figure S16 — Phylogenies by the ML method for lipid synthesis genes that were inferred to have originated from secondary endosymbionts. DGAT-2 from N. oceanica IMET1 are indicated by red stars. (A) Phylogeny of DGAT-2C; (B) phylogeny of DGAT-2B; (C) phylogeny of DGAT-2A; (D) phylogeny of DGAT-2I; (E) phylogeny of DGAT-2G; (F) phylogeny of MCAT. (PDF) [file pgen.1004094.s020.pdf]
