# Supplementary material for: Nannochloropsis Genomes Reveal Evolution of Microalgal Oleaginous Traits
Source: PLoS Genet. 2014 Jan 9;10(1):e1004094. doi: 10.1371/journal.pgen.1004094 (PMC3886936; doi:10.1371/journal.pgen.1004094)
Supplement: Figure S4 — Functional categories of Nannochloropsis core genes. (A) GO Slim categories in “molecular function”. (B) GO Slim categories in “cellular component”. (PDF) [file pgen.1004094.s008.pdf]

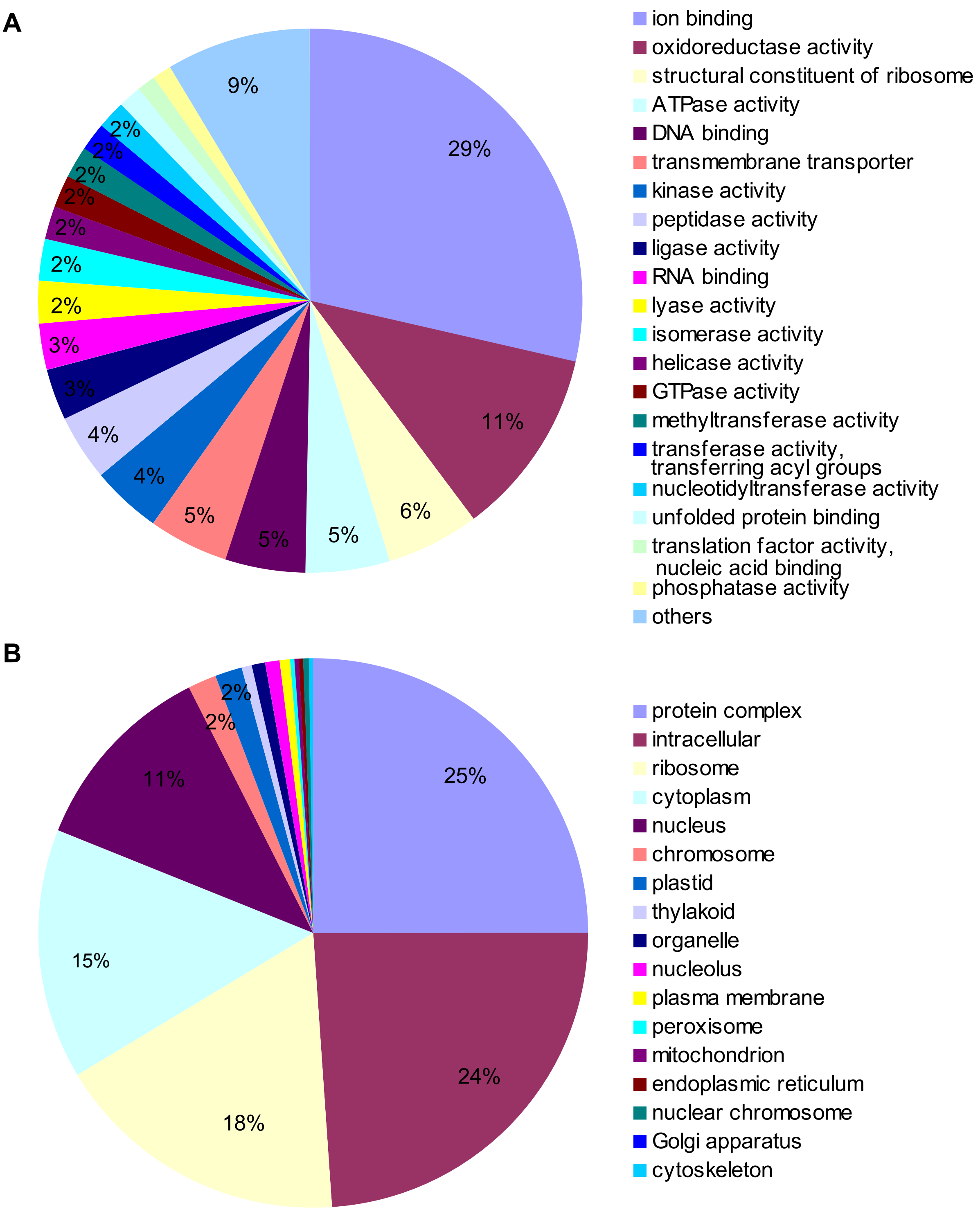
A
29%
11%
6%
5%
5%
5%
4%
4%
3%
3%
2%
2%
2%
2%
2%
2%
2%
9%
ion binding
oxidoreductase activity
structural constituent of ribosome
ATPase activity
DNA binding
transmembrane transporter
kinase activity
peptidase activity
ligase activity
RNA binding
lyase activity
isomerase activity
helicase activity
GTPase activity
methyltransferase activity
transferase activity, transferring acyl groups
nucleotidyltransferase activity
unfolded protein binding
translation factor activity, nucleic acid binding
phosphatase activity
others
B
25%
24%
18%
15%
11%
2%
2%
protein complex
intracellular
ribosome
cytoplasm
nucleus
chromosome
plastid
thylakoid
organelle
nucleolus
plasma membrane
peroxisome
mitochondrion
endoplasmic reticulum
nuclear chromosome
Golgi apparatus
cytoskeleton
