# Supplementary material for: Nannochloropsis Genomes Reveal Evolution of Microalgal Oleaginous Traits
Source: PLoS Genet. 2014 Jan 9;10(1):e1004094. doi: 10.1371/journal.pgen.1004094 (PMC3886936; doi:10.1371/journal.pgen.1004094)
Supplement: Table S1 — Genomic and transcriptomic datasets for the six Nannochloropsis species and strains. (A) Genomic DNA sequencing data for N. oceanica IMET1. (B) Genomic DNA sequencing data for the other six Nannochloropsis strains. (C) cDNA sequencing data for N. oceanica IMET1. The cDNA sequencing was performed on 454 Titanium. (D) mRNA-Seq data for N. oceanica IMET1. SG, shotgun; PE, pair-end; MP, mate-pair. *: Illumina GAIIx–based transcriptome sequencing was performed on total mRNA samples isolated from microalgal cells under both the control growth conditions (indicated as “C”) and the N-depleted conditions (indicated as “N”) at three different time points (3, 6 and 24 h) for each condition. (DOC) [file pgen.1004094.s021.doc]

**Table S1. Genomic and transcriptomic datasets for the five *Nannochloropsis* species and strains.** (**A**) Genomic DNA sequencing data for *Nannochloropsis oceanica* IMET1. (**B)** Genomic DNA sequencing data for the other six *Nannochloropsis* strains. (**C)** cDNA sequencing data for *Nannochloropsis oceanica* IMET1. The cDNA sequencing was performed on 454 Titanium. (**D)** mRNA-Seq data for *Nannochloropsis oceanica* IMET1. SG, shotgun; PE, pair-end; MP, mate-pair. *: Illumina GAIIx based transcriptome sequencing was performed on total mRNA samples isolated from microalgal cells under both the control growth condition (indicated as “**C**”) and the N-depleted condition (indicated as “**N**”), at three different time points (3h, 6h and 24h) for each condition.

| **A. Genome sequencing for *Nannochloropsis* *oceanic*a IMET1** | | | | | | | | | | | | | |
| --- | --- | --- | --- | --- | --- | --- | --- | --- | --- | --- | --- | --- | --- |
| Algal strains | | Platform | | Sequencing library | | | Insert Size (bp) | | Raw Reads | | | Sequencing Depth | |
| *N. oceanica* IMET1 | | 454 | | SG | | | - | | 412,236 | | | 120 | |
| *N. oceanica* IMET1 | | 454 | | SG | | | - | | 624,598 | | |
| *N. oceanica* IMET1 | | 454 | | SG | | | - | | 935,956 | | |
| *N. oceanica* IMET1 | | 454 | | PE | | | 8 K | | 41,621 | | |
| *N. oceanica* IMET1 | | 454 | | PE | | | 8 K | | 556,641 | | |
| *N. oceanica* IMET1 | | 454 | | PE | | | 10 K | | 223,431 | | |
| *N. oceanica* IMET1 | | 454 | | PE | | | 20 K | | 163,873 | | |
| *N. oceanica* IMET1 | | GAIIx | | PE (2x36) | | | 300 | | 14,045,817 | | |
| *N. oceanica* IMET1 | | GAIIx | | PE (2x36) | | | 300 | | 2,962,948 | | |
| *N. oceanica* IMET1 | | GAIIx | | MP (2x36) | | | 2.3 K | | 2,258,799 | | |
| *N. oceanica* IMET1 | | GAIIx | | MP (2x75) | | | 2.3 K | | 4,714,516 | | |
| *N. oceanica* IMET1 | | GAIIx | | PE (2x75) | | | 250 | | 19,768,781 | | |
| **B. Genome sequencing for the other six *Nannochloropsis* strains** | | | | | | | | | | | | | |
| *N. oculata* CCMP525 | | GAIIx | | PE (2x100) | | | 400 | | 7,352,859 | | | 56 | |
| *N. oculata* CCMP525 | | GAIIx | | MP (2x100) | | | 3 K | | 6,607,223 | | |
| *N. granulata* CCMP529 | | GAIIx | | PE (2x100) | | | 400 | | 9,457,329 | | | 72 | |
| *N. granulata* CCMP529 | | GAIIx | | MP (2x100) | | | 3 Kb | | 1,129,002 | | |
| *N. oceanica* CCMP531 | | GAIIx | | PE (2x100) | | | 400 | | 12,741,009 | | | 70 | |
| *N. oceanica* CCMP531 | | GAIIx | | MP (2x100) | | | 3 Kb | | 3,415,226 | | |
| *N. salina* CCMP537 | | GAIIx | | PE (2x100) | | | 400 | | 9,434,326 | | | 108 | |
| **C. cDNA sequencing data for *Nannochloropsis oceanica* IMET1** | | | | | | | | | | | | | |
| Algal strains | | Platform | | Raw reads | | Genome coverage | | | | Assembled reads/Contigs | | | |
| *N. oceanica* IMET1 | | 454 | | 189,107 | | 45% | | | | 34.9%/13,775 | | | |
| **D. Transcriptome sequencing for *Nannochloropsis oceanica* IMET1*** | | | | | | | | | | | | | |
| Samples | C-3h | | N-3h | | C-6h | | | N-6h | | | C-24h | | N-24h |
| Reads | 3,560,604 | | 4,259,687 | | 9,757,507 | | | 10,323,490 | | | 7,294,502 | | 8,527,729 |
| Nuclear genes covered by mRNA-Seq data | 9,111 (93.4%) | | | | | | | | | | | | |
