# Supplementary material for: Nannochloropsis Genomes Reveal Evolution of Microalgal Oleaginous Traits
Source: PLoS Genet. 2014 Jan 9;10(1):e1004094. doi: 10.1371/journal.pgen.1004094 (PMC3886936; doi:10.1371/journal.pgen.1004094)
Supplement: Table S2 — Comparing the genomic features of N. oceanica IMET1 and five unicellular eukaryotic microalgae. Parameters of the genomes other than IMET1 were retrieved from the literature. (DOC) [file pgen.1004094.s022.doc]

**Table S2. Comparing the genomic features of *Nannochloropsis oceanica* IMET1 and five unicellular eukaryotic microalgae.** Parameters of the genomes other than IMET1 were retrieved from the literature cited.

| **Microalgae** | ***Ostreococcus tauri* [1]** | ***Micromonas pusilla* [2]** | ***Nannochloropsis oceanica* IMET1** | ***Thalassiosira***  ***pseudonana* [3]** | ***Chlorella***  ***variabilis* [4]** | ***Chlamydomonas***  ***reinhardtii* [5]** |
| --- | --- | --- | --- | --- | --- | --- |
| **Genome size** | 12.6Mb | 21.9Mb | 30.1Mb | 32.5Mb | 46.2Mb | 121Mb |
| **G+C content (%)** | 58 | 65 | 54 | 47 | 67 | 64 |
| **Gene number** | 8,166 | 10,575 | 9915 | 11,242 | 9,791 | 15,143 |
| **Gene density (per Kb)** | 0.648 | 0.483 | 0.346 | 0.28 | 0.21 | 0.125 |
| **Coding sequence (%)** | 73.0 | 64.0 | 41.8 | 32.7 | 29.0 | 17.0 |
| **Average Gene length (bp)** | 1,245 | 1,557 | 1,239 | 992 | 2,558 | 4,312 |
| **Average Exon length (bp)** | 750 | 731 | 543 | 334 | 170 | 190 |
| **Exon per gene** | 1.6 | 1.9 | 1.8 | 2.4 | 7.3 | 8.3 |
| **Gene with introns (%)** | 25 | 50 | 38 | 60 | 98 | 92 |
| **Average Intron length(bp)** | 126 | 187 | 307.7 | 147.9 | 209 | 373 |
| **Number of LTRs** | 31 | 154 | 10 | 238 | 867 | 3,380 |
| **Length of LTRs (Kbp)** | 40.0 | 126.3 | 7.3 | 155.5 | 128.1 | 2,877 |

**References cited in Table S2**

1. Palenik B, Grimwood J, Aerts A, Rouze P, Salamov A, et al. (2007) The tiny eukaryote *Ostreococcus* provides genomic insights into the paradox of plankton speciation. P Natl Acad Sci USA 104: 7705-7710.

2. Worden AZ, Panaud, Piegu (2009) Green evolution and dynamic adaptations revealed by genomes of the marine picoeukaryotes *Micromonas*. Science 325: 147-147.

3. Armbrust EV, Berges JA, Bowler C, Green BR, Martinez D, et al. (2004) The genome of the diatom *Thalassiosira pseudonana*: ecology, evolution, and metabolism. Science 306: 79-86.

4. Blanc G, Duncan G, Agarkova I, Borodovsky M, Gurnon J, et al. (2010) The *Chlorella variabilis* NC64A genome reveals adaptation to photosymbiosis, coevolution with viruses, and cryptic sex. Plant Cell 22: 2943-2955.

5. Merchant SS, Prochnik SE, Vallon O, Harris EH, Karpowicz SJ, et al. (2007) The *Chlamydomonas* genome reveals the evolution of key animal and plant functions. Science 318: 245-250.
