# Supplementary material for: Nannochloropsis Genomes Reveal Evolution of Microalgal Oleaginous Traits
Source: PLoS Genet. 2014 Jan 9;10(1):e1004094. doi: 10.1371/journal.pgen.1004094 (PMC3886936; doi:10.1371/journal.pgen.1004094)
Supplement: Text S1 — Detailed descriptions of materials and methods. (DOC) [file pgen.1004094.s023.doc]

**SUPPORTING MATERIALS AND METHODS**

**(1) Selection of *Nannochloropsis* species and strains for genome sequencing**

**Selection strategy**

In selecting the six *Nannochloropsis* strains for genome sequencing, we considered valuable phenotypes (e.g. oil-producing strains), phylogenetic positions and research and industrial interest. The six strains were originally isolated from diverse habitats ranging from fresh to estuarine and oceanic waters. All are oleaginous, producing abundant TAG under environmental stress. For example, *Nannochloropsis oceanica* IMET1, which was originally named as *Nannochloropsis* strain OZ-1 [1,2,3,4] has been widely tested and used as a commercial eicosapentaenoic acid (EPA)- and oil-producer under large-scale outdoor or indoor photosynthetic cultivations in Israel, United States, Japan and China [4]. Therefore, strain IMET1 was chosen for generation of a high quality genome sequence. The other five *Nannochloropsis* strains, all obtained from the CCMP culture collection, were selected for genome sequencing based on the following considerations: first, at least one strain for each known *Nannochloropsis* species was selected; *second*, if there were multiple strains available in a given species, the strain with the most citations in PubMed was selected; third, for the species *N. oceanica*, two strains (CCMP531 and IMET1) were selected to investigate intraspecies genomic variation.

As a result, five *Nannochloropsis* strains selected from four *Nannochloropsis* species were chosen for genome sequencing (**Figure 1A**; **Table S1A**; **Table S1B**): *N. oceanica* strain IMET1, *N. oceanica* strain CCMP531, *N. salina* strain CCMP537, *N. oculata* strain CCMP525, and *N. granulata* strain CCMP529. Genomic sequencing and assembly data of another strain, *N. gaditana* strain CCMP526, were obtained from http://Nannochloropsis.genomeprojectsolutions-databases.com [5].

**Phylogenetic tree based on 18S rDNA sequences**

A maximum likelihood tree for the microalgal lineages was constructed based on 18S rDNA sequences (**Figure S2A**). Evolutionary distances were measured by the number of base substitutions per site. All positions containing alignment gaps and missing data were eliminated in pairwise sequence comparisons. There were a total of 1,729 bases in the final dataset. Phylogenetic analyses were conducted in MEGA5 [6].

**Total lipid content**

*Nannochloropsis* stains were cultivated in modified f/2 liquid medium [7] with 4 mM NO_3_^-^ and were aerated by bubbling with a mixture of 1.5% CO_2_ in air under continuous light (approximately 50 µmol photons m^-2^ s^-1^) at 25˚C. Algal cells were collected during the post exponential growth phase (12 days after inoculation). Total lipids were extracted with chloroform and quantified by the Folch gravimetric method [8], and total lipid content was calculated as total lipid weight divided by dried biomass weight (**Figure S1**). All extractions and measurements were performed with aliquots of algal cells from the same batch of cultivation in triplicates.

**(2) Analysis of *Nannochloropsis* *oceanica* IMET1 transcriptome via mRNA sequencing (mRNA-Seq)**

**Collection**, **sequencing and analysis of IMET1 cDNA for validating gene prediction**

*N.* *oceanica* IMET1 was cultivated in f/2 liquid medium with 4 mM NO_3_^-^ and aerated by bubbling with a mixture of 1.5% CO_2_ under continuous light at 50 µmol photons m^-2^ s^-1^ (defined as the control conditions, C). Mid-logarithmic phase algal cells were collected and washed three times with axenic seawater. Equal numbers of cells were re-inoculated into NO_3_^-^-free f/2 liquid medium under 50 µmol photons m^-2^ s^-1^ (defined as the nitrogen-starvation conditions, N) and f/2 liquid medium with 4 mM NO_3_^-^ under 200 µmol photons m^-2^ s^-1^ (defined as high light conditions, HL). Algal cells grown under the above conditions were collected for total RNA extraction using Trizol (Invitrogen Cat. 15596-018) at 3 h, 6 h and 24 h after re-inoculation. Total RNA from each sample was then pooled to prepare libraries of cDNA for mRNA sequencing on 454 Titanium (Roche, USA). One quarter of a region of 454 was performed, with 189,107 raw reads produced (**Table S1C**). All raw reads were trimmed based on the quality value before further analysis. All cDNA reads that passed quality control were used for transcript-based gene prediction.

**Generation of a single-base resolution transcriptomic program underpinning the full course of nitrogen starvation–induced TAG production in IMET1**

Total RNA samples from the above C and N conditions at the three time points described above were used for mRNA-Seq library preparation and then sequenced on GAIIx (Illumina, USA). In total, six samples from three time points for each of these two conditions were sequenced. For each of the six samples, 3.5 to 10.3 million reads were yielded. When all the reads from the six samples were pooled, 93.4% (9,111 genes) of the total number of predicted protein-coding genes were covered (defined as >80% of the transcribed region mapped by at least 10 reads) (**Table S1D**).

The mRNA reads were mapped with TopHat (v.1.2.0, allowing two mismatches) [9], and those mapped to more than one location were excluded. For each of the mRNA-Seq datasets, gene expression was measured as the number of aligned reads to annotated genes using Cufflinks (v.0.9.3; [10]) and then normalized to FPKM values (Fragments Per Kilobase of exon model per Million mapped fragments). Predicted genes with expression values (FPKM) less than five were filtered out before differential gene expression analysis. For each time point sampled, up- and down-regulation of gene expression (N as compared to C) were quantified by the fold change of FPKM values.

**(3) Characterizing and sequencing the *Nannochloropsis* *oceanica* IMET1 genome**

**Pulsed-field gel electrophoresis**

*N. oceania* IMET1 was grown in f/2 medium under a 12:12 h light-dark cycle with a light intensity of 50 µmol photons m^-2^ s^-1^ at 22˚C. Aliquots (50 ml) of algal cells were harvested at late logarithmic phase (1×10^8^ cells ml^-1^) via centrifugation. Pellets were resuspended in fresh f/2 medium to a final cell concentration of 5×10^9^ cells ml^-1^. To prepare agarose plugs for pulsed-field gel electrophoresis (PFGE), 1 ml of microalgal cells was spun down and resuspended in the same volume of prewarmed Buffer A [450 mM EDTA, 10 mM Tris-HCl (pH 8) and 100 mM NaCl] and placed in a 50˚C water bath for 5 min. The cell suspension was mixed with 1 ml 1.0% “InCert” agarose (Cambrex Bio Science Rockland, Inc., Rockland, ME, USA) in 125 mM EDTA and 10 mM Tris-HCl (pH 8) solution containing 100 mM 2-mercaptoethanol (BME) and 1 mg ml^-1^ lysozyme at 50˚C. The mixture was pipetted into plug molds and solidified for 8 min at -20°C. Plugs were serially washed in different buffers as follows: 10 mL lysozyme solution [500 mM EDTA (pH 8), 10 mM Tris-HCl (pH 8), 1% sodium lauryl/sarcosinate and 1 mg ml^-1^ lysozyme] at 37˚C overnight; 5 ml Proteinase K solution [500 mM EDTA (pH 8), 10 mM Tris-HCl (pH 8), 1% sodium lauryl/sarcosinate and 0.2 mg ml^-1^ Proteinase K] at 50˚C for 24 hours; and 1.5 ml Buffer A at 50˚C for 4 hours (twice). The plugs were then stored in Buffer A at 4˚C for future use. A CHEF-DRII Pulsed Field Electrophoresis System (contour-clamped homogeneous electric field) (Bio-Rad Laboratories, Hercules, CA, USA) was used in this study to perform PFGE. Chromosomes ranged from 100 to 2,000 Kb in size and were separated using the method modified from Nosenko *et al* [11]. Briefly, 1% pulsed field certified agarose gel was run in 0.5 × TBE buffer at 12˚C under the following conditions: Stage I: 0.9 v/cm, 500 s switch time, 3.5 h run time, 120˚ included angle; Stage II: 6 v cm^-1^, 60 s switch time, 15 h run time, 120˚ included angle; and Stage III: 6 v cm^-1^, 120 s switch time, 11.5 h run time, 120˚ included angle. Chromosomes larger than 2,000 Kb in size were separated using 0.8% agarose gel in 1 × TAE buffer (4.84 g Tris base in 250 ml ddH_2_O, 1.14 ml acetic acid, 2 ml 0.5M EDTA pH 8.0 L^-1^) under the following conditions: 2 v cm^-1^, 1800 s switch time, 72 h run time, 106˚ included angle. Three DNA size standards—*Saccharomyces cerevisiae* (240-2,200 Kb, Marker A), *Hansenula wingei* (1-3.1 Mb, Marker B), and *Schizosaccharomyces pombe* (3.5-5.7 Mb, Marker C)—were used to estimate chromosome sizes. Pulsed-field gels were stained with ethidium bromide and scanned using a Gel Logic 200 Imaging System. Profiles were analyzed with ImageJ (http://rsbweb.nih.gov/ij/) to detect and quantify every band.

Fifteen bands were identified from two different pulsed-field gel profiles (**Figure S3**). These bands corresponded to chromosomes of the following sizes: 3,700, 2,810, 1,900, 1,440, 1,385*, 1,275*, 1,100*, 985*, 895*, 760*, 725*, 690, 660, 645 and 600 Kb; bands marked with asterisks exhibited greater intensity than the others, indicating that these bands likely contain more than one chromosome. Here, these denser bands were assumed to comprise two chromosomes of similar sizes. Therefore, the estimated total genome size of *N. oceanica* IMET1from our PFGE study is ~26,695 Kb. Previous studies showed that a 20% underestimation of genome size is common when PFGE is used to investigate genome size [12,13]. Correcting for this possible underestimation, the genome size of *N. oceanica* IMET1 is within a range of 26,695 Kb to 33,369 Kb. This supported the 30.1 Mb total genome size revealed by whole-genome sequencing (below).

**Strategy for genome sequencing**

For sampling and sequencing the *Nannochloropsis* genomes and the IMET1 transcriptome, our sequencing strategy took advantage of the complementarity between 454 Titanium and GAIIx in terms of read length, sequencing throughput, sequencing depth, sequencing bias, etc. [14]. For the isolation of genomic DNA, all *Nannochloropsis* strains were first made sterile and picked as single colonies on agar plates as culture inocula. Unless otherwise indicated, strains were grown in flasks with 500 ml modified BG-11 media with filtered seawater for 7-10 days. Algal cells were collected through centrifugation at 5000 g for 5 min, followed immediately by CTA extraction of genomic DNA.

**Genome sequencing, assembly and improvement**

For *N. oceanica* strain IMET1, we collected shotgun and mate-paired reads from both 454 Titanium and GAIIx. We first generated a total of 30X 454-Titanium sequence-coverage (average read length 400-500 bp, with different pair-distances of 8, 10 and 20 Kb). Furthermore, we generated a total of 108X GAIIx sequence coverage with an average read length of 75 bp and pair-distances of 300 bp and 2.3 Kb (**Table S1A**). The shotgun and pair-ended 454 reads were assembled using Newbler (Roche, USA). GAIIx reads were utilized in a two-stage assembly-improvement process (as described below).

During stage I of assembly improvement (gap-filling), all GAIIx reads were mapped to the 454 assembly; paired reads spanning a gap were identified and used as anchors for a local assembly with all unmapped GAIIx reads; the resulting GAIIx-only contigs were individually integrated into the 454 assembly for gap-filling using Consed [15] after manual inspections. During stage II of assembly improvement (scaffold building), all paired GAIIx reads were mapped to the 454-contigs. For each read, only one best MAQ-hit was recorded (http://maq.sourceforge.net/), which would randomly choose a hit position for output when multiple best hits emerged. Those that spanned different contigs or scaffolds in the 454-assembly were identified. These candidate bridges underwent the following validation before being used for scaffold building as reliable bridges: (1) the length of the bridge had to fall within the expected insert size of the libraries it originated from; (2) for each potential inter-contig or inter-scaffold gap spanned, the bridges had to originate from at least two independently constructed libraries; and (3) those bridges with either or both of the end-reads mapped to more than two contigs were not considered. In the end, those inter-contig or inter-scaffold gaps that were spanned by at least eight such reliable bridges were identified as additional links, which were then used to manually order and orientate contigs and scaffolds.

The machine-annotated scaffolds were further assembled based on the manually annotated contig connections, which reduced the number of scaffolds from 355 to 296. The assembled genome sequences were further screened and filtered by searching against bacterial sequences from the SILVA [16] and the NCBI non-redundant (NR) databases. The number of IMET1 scaffolds was thus reduced to 294. In the end, the IMET1 genome assembly consisted of 293 scaffolds totaling 31.5 Mb with a contig N50 size of 51 Kb and a scaffolds N50 size of 935 Kb.

**(4) Sequencing the four *Nannochloropsis* strains other than *N. oceanica* IMET1**

**Genome sequencing**

For each of the four strains, we collected paired GAIIx reads (**Table S1B**). All GAIIx reads for each strain were assembled using Velvet [17] with a specified insert size (*k*-mer size = 35). The genome assemblies revealed genome sizes that ranging from 25.38 to 32.07 Mb, with contig N50 size in the range of 15 to 38Kb.

For each of the five *Nannochloropsis* strains (including IMET1), assembly and finishing of the mitochondrial and chloroplast genomes was completed via iterations of custom primer–based chromosome walking, local assembly of the finishing reads and manual inspection of the assemblies. The parameters for the organelle genomes are listed in **Table 1**.

**Quality assessment of the genome assemblies**

We first examined the IMET1 genome assembly. More than 90% of the scaffolds were greater than 1000 bp in length, and more than 90% of predicted genes (see below) were from scaffolds longer than 1000 bp. Predicted genes on longer scaffolds were more likely to have hits to functional genes (those that are not hypothetical or conserved hypothetical genes) in the NCBI NR database. Moreover, 80% of genes on scaffolds longer than 1000 bp were full-length genes (i.e., aligning to >90% of the full-length subject genes in a BlastP search versus the NCBI NR database).

Genome assemblies for the other five strains (including CCMP526, downloaded from http://Nannochloropsis.genomeprojectsolutions-databases.com/) were 26.9-35.5 Mb in size, similar to *N. oceanica* IMET1 (30.1 Mb). They encoded similar numbers of genes to IMET1 (**Table 1**). The gene density per Kb (0.20-0.30) on these genomes was lower than *N. oceanica* IMET1 (0.33). The proportions of the genes that have blast hits in the NCBI NR database (49.0%-62.6%) were slightly lower than IMET1 (69.2%).

**(5) Identification and annotation of functional elements in the *Nannochloropsis* genomes**

**Gene prediction and quality assessment**

For the IMET1 genome, genes were predicted by AUGUSTUS [18] (v2.5) which combined the *ab initio* predictions with predictions based on cDNA read alignments (387 K aligned cDNA reads from a Roche 454 Sequencer), with alternative splicing form predicting module turned off. The predicted genes were first validated by our experimentally determined mRNA-Seq data under C and N conditions (12 datasets representing three points from each of the two conditions; see above). We used Cufflinks to measure the level of gene expression based on 50 bp reads from GAIIx. For a given gene, if no gene expression was detected by Cufflinks, it was considered “not observed” from transcriptome sequencing data. In strain IMET1, 98.9% of genes were “observed”, indicating a ≤6% false positive rate. On the other hand, the false negative rate was <10% when gene structures predicted by Cufflinks were used as references.

We then examined the structural and functional features of the predicted genes. Firstly, the predicted gene length distribution of IMET1 (52% of the genes were of 200-400 bp) was very similar to the distributions reported for *C. reinhardtii* (56% of the genes were 200-400 bp; [19]) and *T. pseudonana* (49% of the genes were 200-400 bp; [20]). Secondly, genes from IMET1 that had hits in the NCBI NR database tended to be longer (most frequent gene length was ~400 bp) than genes that had no NCBI NR hit (most frequent gene length ~200 bp), a phenomenon similar to *C. reinhardtii* (most frequent gene length ~200 bp for hits, ~100 bp for non-hits) and *T. pseudonana* (most frequent gene length ~300 bp for hits, ~200 bp for non-hits). Thirdly, more than 80% of the genes that had hits in the NCBI NR database were full-length genes that aligned to >90% bases of the full-length subject genes).

**Functional annotation of protein-coding genes**

Predicted protein-coding genes were then annotated by searching against three databases: the NCBI NR and the Kyoto Encyclopedia of Genes and Genomes (KEGG) databases by BlastP, and the Gene Ontology (GO) database by InterProScan [21]. For each of the predicted proteins, its hit with the highest sequence identity in NCBI NR was determined using BlastP. A protein was annotated as a hypothetical protein if there were no sequence homologs in NCBI NR and as a conserved hypothetical protein if its best hits in NCBI NR were annotated as a “hypothetical protein”. Functional proteins were generally longer than conserved hypothetical proteins, and the hypothetical proteins had the shortest length.

**Identification and annotation of RNA-coding genes**

The locations of tRNA were predicted using tRNAscan-SE (v.1.21; [22]) . Loci encoding rRNA were identified via BlastN search against ribosomal RNA sequences from the RNAmmer database (v.1.2m, retrieved June 1^st^, 2011; [23]). Hundreds of rRNA and 80 tRNA were identified in the IMET1 genome.

**(6) Analysis of the structure and function of the *Nannochloropsis* genomes**

**Global comparison of genome-encoded functions**

Gene Ontology (GO) categories and InterPro ID numbers were assigned using InterProScan (Perl-based v.4.6; [21]). The number of genes assigned to each GO term, or to its parents in the hierarchy (according to the ontology description available as of Jan. 2013, including all GO terms and generic GO slim terms; [24]), were totaled. Genes that could not be assigned to a GO category were excluded. For GO terms with significant variations in abundance among the genomes, their subcategory (“child”) GO terms were then further investigated to pinpoint the lower-level GO terms that contributed to the variation.

**Reconstruction of metabolic pathways**

KEGG IDs associated with each predicted protein-coding gene in *Nannochloropsis* were obtained, when applicable, by searching the protein sequence against the KEGG database with an e-value cutoff at 1e-5. Best hits and best known matched KEGG IDs (i.e., the best hit with a subject of known function) were collected to map to metabolic pathways using the iPATH tools. Sub-cellular localization of proteins were predicted by ChloroP, TargetP [25], PredAlgo [26] and HECTAR [27].

**Identification of core and accessory proteomes**

To clarify the functional diversity of the *Nannochloropsis* genome, we identified the “*Nannochloropsis*-core” proteins as the intersections of the five “IMET1-pairwise cores” and “IMET1-only accessory” proteins as the intersections of the five “IMET1-pairwise accessories”. To obtain the IMET1-pairwise cores and IMET1-pairwise accessories, all proteins from IMET1 (i.e., Genome-A) were searched against all proteins from each of the other five *Nannochloropsis* genomes (Genome-B) by BlastP with an e-value cutoff at 1e-5 and a protein sequence identity cutoff at 80%. To avoid omitting alignments due to gene prediction errors, all proteins from IMET1 (Genome-A) were searched against each Genome-B by tBlastN with the above e-value and protein sequence identity cutoffs. Proteins in IMET1 that failed to align to Genome-B by either BlastP or tBlastN were considered IMET1-pairwise accessories, while others were labeled as IMET1-pairwise cores.

To calculate the pan-genome size of *Nannochloropsis*, we started with the IMET1 genome as the subject database and proteins from CCMP531 as the query to obtain the number of pairwise accessories, which was then added to the total number of IMET1 genes as the pan-genome size of IMET1 and CCMP531. The IMET1 genome and CCMP531 genome were then put together as a database when the next proteome CCMP529 was included as a query, and the number of pairwise accessories derived was added again. Each of the *Nannochloropsis* proteomes was included sequentially, and the final pan-genome size of *Nannochloropsis* was thus derived (**Figure 1C***)*. The *Nannochloropsis* core size was calculated by reducing the number of pairwise cores produced when each proteome was included from the originals that started from the total number of IMET1 proteins (**Figure 1C**).

**(7) Evolutionary analysis of the *Nannochloropsis* genomes**

**Orthologs** **and** **paralogs**

The orthologs and paralogs among the six strains were identified by a Markov Clustering algorithm (OrthoMCL [28], v. 4) with an inflation index of 1.5. The protein-coding gene set for each of the genomes was searched against all genes in the six genomes by BlastP with an e-value cutoff value of 1e-5. The ortholog groups were then generated by MCL with an inflation index of 1.5 [28], in which each of the genes was an ortholog to all other members of the same group. In-paralogous proteins in the genomes were also identified by OrthoMCL.

**Generation of whole-genome phylogenetic tree for the six *Nannochloropsis* strains**

We have used the method described for the 12 *Drosophila* genomes [29] to generate the whole-genome phylogeny of the six *Nannochloropsis* spp. There were 1,085 orthologous gene sets from the six strains, with each of the orthologous gene-sets harboring one and only one ortholog from each strain. For each of the orthologous gene sets, the encoded protein sequences were aligned by MUSCLE (v. 3.7; [30]). The alignments were curated by GBlock (v.0.91b; [31]) to filter out poorly aligned positions. The curated alignments were then analyzed by PhyML (v.3.0; [32]) to generate ML trees using the Poisson model and the bootstrapping method (based on 1,000 replicates). A consensus tree was then constructed for all of the orthologous gene sets.

**Selection pressure of protein-coding genes**

PAML (v.4.4c; [33]) codon substitution models and likelihood ratio tests (codeml) were used to estimate the rate of evolution and to test selection pressure. For each gene set in the six-set single-copy ortholog genes, PAML Model M0, M7 and M8 were run with branch lengths as free parameters, and codon frequencies were estimated by F3x4. PAML Model M0 was used to estimate a single ω (K_a_/K_s_, ratio of non-synonymous to synonymous divergence) that was fixed across the phylogeny for each alignment (referred to as ω of a gene). In order to avoid convergence problems, we ran each analysis three times with different initial values of ω and adopted results from the run with the highest likelihood.

To connect gene function with sequence evolution, GO term assignments for each of the genes were retrieved from InterProScan results. Since GO slims are particularly useful for giving a summary of the genome-wide GO annotation, all GO terms were mapped to GO slim (<http://www.geneontology.org/GO.slims.shtml>). Only those GO terms associated with five or more genes were plotted. At the genus level, the six-set single-copy orthologous genes from the six *Nannochloropsis* strains were mapped to the ontology of 59 functional categories; 25 described a molecular function, 9 described a cellular component and 25 described a biological process.

For each gene, the relevant parameters (ω, K_a_, etc.) were obtained from the PAML results described above. For each of the functional categories, the ω value was estimated as the average among all genes belonging to the same category. The selection pressures of core and accessory genes were analyzed respectively using a method similar to the selection pressure analysis of protein-coding genes.

**Horizontal gene transfer (HGT)**

We implemented the approach described in Schonknecht, et al. [34] to identify HGT genes in IMET1. We started by collecting 441 sequenced genomes that included model organisms, all published algal genomes and those that harbored best Blast hits of IMET1 proteins in the NCBI NR database. InParanoid (v.2; [35]) with default parameters was used to search for orthologous groups between proteins in IMET1 and proteins from these 441 genomes. Orthologous groups with score 1 were chosen for further analysis. The IMET1 proteins were classified into two categories based on the InParanoid results: Category 1 for those giving only Blast hits in bacterial or archaeal sequences, and Category 2 for those giving Blast hits in bacterial or archaeal sequences in addition to hits in eukaryotic sequences. Both categories were selected as initial HGT candidates for further phylogenetic analysis as described below.

We used stringent criteria for our phylogenetic analyses, similar to the criteria of Schonknecht, et al. [34]: i) proteins that were shorter than 150 amino acids (and thus were not able to build reliable MSAs) were not accepted; ii) those phylogenetic trees that included fewer than ten species were excluded and removed; iii) in order to discriminate against endosymbiotic gene transfer, proteins that were potentially transferred from cyanobacteria were accepted as HGT candidates only when their homologs were absent from other photosynthetic eukaryotes and were not associated with photosynthetic functions; and iv) when a phylogenetic tree did not allow for conclusions about the origin of the gene, the gene was removed from the list of candidates. Those Category 1 proteins that met the criteria above were labeled as HGT candidates.

For Category 2 proteins, we conducted the further phylogenetic analyses. Multiple sequence alignment for each of the proteins and their orthologs was performed using MUSCLE with the maximum number of iterations set to 100, followed by GBlock curation (parameters: -b3=8, –b4=2, –n=y) to remove poorly aligned regions [30,31]. The best protein evolution model for each MSA was selected using ProtTest [36] and was used to reconstruct the phylogenetic relationships for the proteins in the MSA by PhyML [32] with 100 bootstrapping replicates. NJ trees were also reconstructed for each MSA by MEGA5 [6] with 100 bootstrapping replicates. The phylogenetic tree for each HGT candidate was manually checked and only accepted when a clear pattern of HGT was observed in both NJ and ML trees. The manual inspection identified 99 HGT candidates. For each candidate, both NJ and ML trees in NEWICK format are listed in **Dataset S3**. For a detailed description of the methodology, please refer to Schonknecht, et al. [34].

**Evolutionary origin of lipid synthesis genes**

We carried out a detailed phylogenetic analysis of the *Nannochloropsis* lipid biosynthesis genes to investigate their evolutionary origin. To reduce the bias in taxon sampling, the strategy described in Chan et al. [37] was implemented to build a comprehensive database and to construct the homologous groups for each lipid synthesis gene for phylogenetic analysis. The database contained all sequenced genomes from RefSeq and Joint Genome Institute (ftp://ftp.jgi-psf.org/pub/JGI_data/) as well as EST sequences from dbEST and TBestDB. Genomes of red algae (*Cyanidioschyzon merolae* [38], *Galdieria sulfuraria* [34], *Porphyridium purpureum* [39], and *Condrus crispus* [40]) and all the EST datasets for red algae were included. Each lipid synthesis gene in IMET1 was first searched against the database using BlastP with an e-value cutoff of 1e-10. Proteins of the resultant top five hits were used as a query to search against the database again, generating five lists of BlastP hits. The original BlastP hits of IMET1 query proteins and the five lists were grouped together to build the homologous groups. For each group, we adopted a sampling criterion similar to Chan et al. [37] to ensure reasonable taxon sampling, using a customized script (http://www.bioenergychina.org/fg/d.wang_scripts/). Multiple sequence alignments were performed using ClustalW in MEGA5 [6]. Homologs in bacteria and metazoan were used as outgroups. Both ML and NJ trees were constructed based on the Poisson correction model in MEGA5 with the bootstrapping method (based on 100 replicates). A gene was inferred to be potentially from a green or red algae related secondary endosymbiont when its phylogent was supported by both NJ and ML trees. Manual inspection on the phylogenetic trees (**Figure S15, Figure S16**) inferred that *DGAT-2C* originated from a red algal endosymbiont, *DGAT-2A*, *DGAT-2B*, *DGAT-2G* and *DGAT-2I* from a green algal endosymbiont, and others potentially from the heterotrophic secondary host.

The phylogenetic relationship among the 74 *DGAT*s (including both *DGAT-1*s and *DGAT-2*s) from the six *Nannochloropsis* strains were inferred by constructing NJ trees in MEGA5 (**Figure S14**). *DGAT* homologs from other model organisms [including green algae (*Chlamydomonas reinhardtii* and *Ostreococcus tauri*), red algae (*Cyanidioschyzon merolae*, *Galdieria sulfuraria* and all the EST sequences in other red algae available in public databases), higher plants (*Arabidopsis thaliana*), heterokonts (the diatoms *Thalassiosira pseudonana* and *Phaeodactylum tricornutum*) and bacteria] were also included in this tree. Homologs of DGAT in these models were identified through BlastP against proteomes, tBlastN against genomes and ESTs using *Nannochloropsis* genes as queries, followed by manual curation on the resulting candidates by investigating their functional annotation, conserved domains and phylogeny.

**Reference**

1. Cheng-Wu Z, Zmora O, Kopel R, Richmond A (2001) An industrial-size flat plate glass reactor for mass production of *Nannochloropsis* sp. (Eustigmatophyceae). Aquaculture 195: 35-49.

2. Richmond A, Cheng-Wu Z (2001) Optimization of a flat plate glass reactor for mass production of *Nannochloropsis* sp. outdoors. J Biotechnol 85: 259-269.

3. Zittelli GC, Lavista F, Bastianini A, Rodolfi L, Vincenzini M, et al. (1999) Production of eicosapentaenoic acid by *Nannochloropsis* sp cultures in outdoor tubular photobioreactors. J Biotechnol 70: 299-312.

4. Zittelli GC, Rodolfi L, Tredici MR (2003) Mass cultivation of *Nannochloropsis* sp. in annular reactors. J Appl Phycol 15: 107–114.

5. Radakovits R, Jinkerson RE, Fuerstenberg SI, Tae H, Settlage RE, et al. (2012) Draft genome sequence and genetic transformation of the oleaginous alga *Nannochloropsis gaditana*. Nat Commun 3: 686.

6. Tamura K, Peterson D, Peterson N, Stecher G, Nei M, et al. (2011) MEGA5: molecular evolutionary genetics analysis using maximum likelihood, evolutionary distance, and maximum parsimony methods. Mol Biol Evol 28: 2731-2739.

7. Dong HP, Williams E, Wang DZ, Xie ZX, Hsia RC, et al. (2013) Responses of *Nannochloropsis oceanica* IMET1 to long-term nitrogen starvation and recovery. Plant Physiol 162: 1110-1126.

8. Folch J, Lees M, Sloane Stanley GH (1957) A simple method for the isolation and purification of total lipides from animal tissues. J Biol Chem 226: 497-509.

9. Trapnell C, Pachter L, Salzberg SL (2009) TopHat: discovering splice junctions with RNA-Seq. Bioinformatics 25: 1105-1111.

10. Trapnell C, Williams BA, Pertea G, Mortazavi A, Kwan G, et al. (2010) Transcript assembly and quantification by RNA-Seq reveals unannotated transcripts and isoform switching during cell differentiation. Nat Biotechnol 28: 511-515.

11. Nosenko T, Boese B, Bhattacharya D (2007) Pulsed-field gel electrophoresis analysis of genome size and structure in *Pavlova gyrans* and *Diacronema* sp (Haptophyta). J Phycol 43: 763-767.

12. Courties C, Perasso R, Chretiennot-Dinet MJ, Gouy M, Guillou L, et al. (1998) Phylogenetic analysis and genome size of *Ostreococcus tauri* (Chlorophyta, Prasinophyceae). J Phycol 34: 844-849.

13. Takahashi H, Takano H, Yokoyama A, Hara Y, Kawano S, et al. (1995) Isolation, characterization and chromosomal mapping of an actin gene from the primitive red alga *Cyanidioschyzon merolae*. Curr Genet 28: 484-490.

14. Dangl JL, Reinhardt JA, Baltrus DA, Nishimura MT, Jeck WR, et al. (2009) De novo assembly using low-coverage short read sequence data from the rice pathogen *Pseudomonas syringae pv. oryzae*. Genome Res 19: 294-305.

15. Gordon D, Desmarais C, Green P (2001) Automated finishing with Autofinish. Genome Res 11: 614-625.

16. Pruesse E, Quast C, Knittel K, Fuchs BM, Ludwig W, et al. (2007) SILVA: a comprehensive online resource for quality checked and aligned ribosomal RNA sequence data compatible with ARB. Nucleic Acids Res 35: 7188-7196.

17. Zerbino DR, Birney E (2008) Velvet: algorithms for de novo short read assembly using de Bruijn graphs. Genome Res 18: 821-829.

18. Stanke M, Morgenstern B (2005) AUGUSTUS: a web server for gene prediction in eukaryotes that allows user-defined constraints. Nucleic Acids Res 33: W465-467.

19. Merchant SS, Prochnik SE, Vallon O, Harris EH, Karpowicz SJ, et al. (2007) The *Chlamydomonas* genome reveals the evolution of key animal and plant functions. Science 318: 245-250.

20. Armbrust EV, Berges JA, Bowler C, Green BR, Martinez D, et al. (2004) The genome of the diatom *Thalassiosira pseudonana*: ecology, evolution, and metabolism. Science 306: 79-86.

21. Quevillon E, Silventoinen V, Pillai S, Harte N, Mulder N, et al. (2005) InterProScan: protein domains identifier. Nucleic Acids Res 33: W116-120.

22. Schattner P, Brooks AN, Lowe TM (2005) The tRNAscan-SE, snoscan and snoGPS web servers for the detection of tRNAs and snoRNAs. Nucleic Acids Res 33: W686-689.

23. Lagesen K, Hallin P, Rodland EA, Staerfeldt HH, Rognes T, et al. (2007) RNAmmer: consistent and rapid annotation of ribosomal RNA genes. Nucleic Acids Res 35: 3100-3108.

24. Ashburner M, Ball CA, Blake JA, Botstein D, Butler H, et al. (2000) Gene ontology: tool for the unification of biology. The Gene Ontology Consortium. Nat Genet 25: 25-29.

25. Emanuelsson O, Brunak S, von Heijne G, Nielsen H (2007) Locating proteins in the cell using TargetP, SignalP and related tools. Nat Protoc 2: 953-971.

26. Tardif M, Atteia A, Specht M, Cogne G, Rolland N, et al. (2012) PredAlgo: a new subcellular localization prediction tool dedicated to green algae. Mol Biol Evol 29: 3625-3639.

27. Gschloessl B, Guermeur Y, Cock JM (2008) HECTAR: A method to predict subcellular targeting in heterokonts. BMC Bioinformatics 9: 393.

28. Li L, Stoeckert CJ, Jr., Roos DS (2003) OrthoMCL: identification of ortholog groups for eukaryotic genomes. Genome Res 13: 2178-2189.

29. Stark A, Lin MF, Kheradpour P, Pedersen JS, Parts L, et al. (2007) Discovery of functional elements in 12 *Drosophila* genomes using evolutionary signatures. Nature 450: 219-232.

30. Edgar R (2004) MUSCLE: a multiple sequence alignment method with reduced time and space complexity. BMC Bioinformatics 5: 113.

31. Talavera G, Castresana J (2007) Improvement of phylogenies after removing divergent and ambiguously aligned blocks from protein sequence alignments. Syst Biol 56: 564-577.

32. Guindon S, Dufayard JF, Lefort V, Anisimova M, Hordijk W, et al. (2010) New algorithms and methods to estimate maximum-likelihood phylogenies: assessing the performance of PhyML 3.0. Syst Biol 59: 307-321.

33. Yang Z (2007) PAML4: phylogenetic analysis by maximum likelihood. Mol Biol Evol 24: 1586 - 1591.

34. Schonknecht G, Chen WH, Ternes CM, Barbier GG, Shrestha RP, et al. (2013) Gene transfer from bacteria and archaea facilitated evolution of an extremophilic eukaryote. Science 339: 1207-1210.

35. Ostlund G, Schmitt T, Forslund K, Kostler T, Messina DN, et al. (2010) InParanoid 7: new algorithms and tools for eukaryotic orthology analysis. Nucleic Acids Res 38: D196-203.

36. Abascal F, Zardoya R, Posada D (2005) ProtTest: selection of best-fit models of protein evolution. Bioinformatics 21: 2104-2105.

37. Chan CX, Reyes-Prieto A, Bhattacharya D (2011) Red and green algal origin of diatom membrane transporters: insights into environmental adaptation and cell evolution. PLoS One 6: e29138.

38. Matsuzaki M, Misumi O, Shin-I T, Maruyama S, Takahara M, et al. (2004) Genome sequence of the ultrasmall unicellular red alga *Cyanidioschyzon merolae* 10D. Nature 428: 653-657.

39. Bhattacharya D, Price DC, Chan CX, Qiu H, Rose N, et al. (2013) Genome of the red alga *Porphyridium purpureum*. Nat Commun 4: 1941.

40. Collen J, Porcel B, Carre W, Ball SG, Chaparro C, et al. (2013) Genome structure and metabolic features in the red seaweed *Chondrus crispus* shed light on evolution of the Archaeplastida. Proc Natl Acad Sci USA 110: 5247-5252.
