# Supplementary material for: Nannochloropsis Genomes Reveal Evolution of Microalgal Oleaginous Traits
Source: PLoS Genet. 2014 Jan 9;10(1):e1004094. doi: 10.1371/journal.pgen.1004094 (PMC3886936; doi:10.1371/journal.pgen.1004094)
Supplement: Text S2 — The selective pressure that drives genome diversity in Nannochloropsis spp. (DOC) [file pgen.1004094.s024.doc]

**SUPPORTING TEXT**

Using *Nannochloropsis* as a model, we examined possible selective pressures (as defined by K_a_/K_s_; [1]) driving microalgal genome divergence. To compare K_a_/K_s_ ratios between the core and accessory genes, we first identified all single-copy orthologous groups from five strains (excluding *N.gaditana*). Among these, groups harboring the previously defined core genes (i.e., the core from the six strains, as represented by IMET1 genes) and the remaining groups were subject to K_a_/K_s_ analysis to deduce selective pressure that driving the evolution of the core and accessory genes, respectively. A significant difference was found between core and accessory genes (*p*<0.001; **Figure S7A**); accessory genes tended to have higher K_a_/K_s_ ratios, especially those involved in catabolic process (GO:0009056), transmembrane transport (GO:0055085), cofactor metabolism (GO:0051186) and cellular amino acid metabolism (GO:0006520). These functional categories were the most constrained in the core genes but the least constrained in the accessory ones (**Figure S7B**). However, genes related to transport (GO:0006810) and vesicle-mediated transport (GO:0016192) were among the most constrained categories in both groups.

To test the link between evolution and function of the core, 1,085 single-copy ortholog groups (those with a single orthologous gene from each of the six strains) were identified (**Text S1**). Mapping the 1,085 sets of single-copy orthologs to the whole-genome phylogeny enabled tracing the selection pressure at every branch along the *Nannochloropsis* evolution. At the genus-level, 59 functional categories were found: 25 in biological process, 25 in molecular function and 9 in cellular component (**Figure S8**). The most constrained biological processes (defined by K_a_/K_s_) included vesicle-mediated transport, generation of precursor metabolites and energy, and photosynthesis, whereas the least constrained ones included signal transduction, cellular component assembly and tRNA metabolic process.

Furthermore, the K_a_/K_s_ ratio of a gene was negatively correlated with its transcriptional level (spearman *r_s_*=-0.453, *p*=0.00, **Figure S7C**). This observation was further confirmed by comparing the transcript abundance of core and accessory genes (**Figure S7D**). The transcriptional levels of the core genes were significantly higher than the accessory (*p* <0.001). Furthermore, in the 12 core genes with extraordinary high transcription [FPKM (Fragments Per Kilobase of exon per Million mapped reads) >2,000), 10 were under strongly purifying selective pressure, with K_a_/K_s_<0.03 (except for the two encoding glycolysis enzymes, which function in photosynthesis, mostly as light harvesting proteins). Thus, both biological function and expression level might have shaped the evolution of *Nannnochloropsis* genes [2,3].

**References**

1. Jordan IK, Rogozin IB, Wolf YI, Koonin EV (2002) Essential genes are more evolutionarily conserved than are nonessential genes in bacteria. Genome Res 12: 962-968.

2. Mata J, Bahler J (2003) Correlations between gene expression and gene conservation in fission yeast. Genome Res 13: 2686-2690.

3. Wall DP, Hirsh AE, Fraser HB, Kumm J, Giaever G, et al. (2005) Functional genomic analysis of the rates of protein evolution. Proc Natl Acad Sci USA 102: 5483-5488.
